# Nine New Pregnane Glycosides from the Cultivated Medicinal Plant *Marsdenia tenacissima*

**DOI:** 10.3390/molecules28062705

**Published:** 2023-03-16

**Authors:** Qian-Qian Meng, Shun-Yao Tong, Xing-Rong Peng, Yu-Qing Zhao, Zheng-Hui Li, He-Ping Chen, Ji-Kai Liu

**Affiliations:** 1 School of Pharmacy, Anhui University of Chinese Medicine, Hefei 230012, China; 2 School of Pharmaceutical Sciences, South-Central Minzu University, Wuhan 430074, China; 3 State Key Laboratory of Phytochemistry and Plant Resources in West China, Kunming Institute of Botany, Chinese Academy of Sciences, Kunming 650201, China

**Keywords:** *Marsdenia tenacissima* (Roxb.) moon, asclepiadaceae, pregnane glycosides, structural elucidation, nitric oxide production inhibition

## Abstract

The ethnobotanical plant *Marsdenia tenacissima* has been used for hundreds of years for Dai people in Yunnan Province, China. Previously, chemical investigations on this plant have revealed that pregnane glycosides were the main biological constituents. Nine new pregnane glycosides, marsdeosides A–I (**1**–**9**), were isolated from cultivated dried stems of the medicinal plant *Marsdenia tenacissima* in this study. The structures were analyzed by extensive spectroscopic analysis, including 1D, 2D NMR, HRESIMS, and IR spectroscopic analysis. The absolute configurations of the sugar moieties were identified by comparing the *R*_f_ values and specific optical rotations with those of the commercially available standard samples and the data reported in the literature. Marsdeosides A (**1**) featured an unusual 8,14-*seco*-pregnane skeleton. Compounds **1**, **8**, and **9** showed activity against nitric oxide production in lipopolysaccharide-activated macrophage RAW264.7, with IC_50_ values of 37.5, 38.8, and 42.8 μM (L-NMMA was used as a positive control, IC_50_ 39.3 μM), respectively. This study puts the knowledge of the chemical profile of the botanical plant *M. tenacissima* one step forward and, thereby, promotes the sustainable utilization of the resources of traditional folk medicinal plants.

## 1. Introduction

The plant *Marsdenia tenacissima* (Roxb.) Moon. (*M. tenacissima*) mainly grows in the Yunnan, Guizhou, Sichuan, and Guangxi Provinces in China and other tropical regions, such as India, Myanmar, Sri Lanka, Indonesia, Vietnam, Laos, and Cambodia in Southeastern Asia [1]. In China, it was named as “Tong Guan Teng” and was first recorded in *Dian Nan Ben Cao* (Herbal Medicine of Southern Yunnan), a medicinal classic published during the Ming dynasty in 1436 A.D. This plant, belonging to the Asclepiadaceae family, is a medicinal herb used in traditional Chinese Medicine, as recorded in the 1977, 2010, 2015, and 2020 editions of *Chinese Pharmacopoeia*, and is also widely used in ethnic Dai Medicine by Dai people living in Laos, Myanmar, and Yunnan Province in China as detoxification, swelling-decreasing, and pain-alleviating agents [2]. Nowadays, it is noteworthy that *M. tenacissima* is extensively applied to treat tracheitis, asthma, rheumatalgia, and cancer [3,4,5]. The water extract of the stems and roots of *M. tenacissima* is a commercially available drug in China under the trademark of “Xiao-Ai-Ping”, which is applied in prescribed combinational chemotherapy for treating different cancers, such as liver cancer, stomach cancer, colon cancer, and non-small cell lung cancer [6]. Furthermore, the water extract of *M. tenacissima* is also a main ingredient of many other Chinese prescriptions, such as Shi-Iiao-Cao-Ke-Chuan granule used for treating bronchitis, Lv-Ji-Ke-Chuan granule used for treating cough, Ya-Jie tablets used for relieving stomach illness, and Bai-Jie capsules used for curing swollen throat [7]. The heavy demand for the plant resources of *M. tenacissima* has spawned large-scale cultivation of this plant in the southwest areas of Yunnan Province.

**Figure 1:**
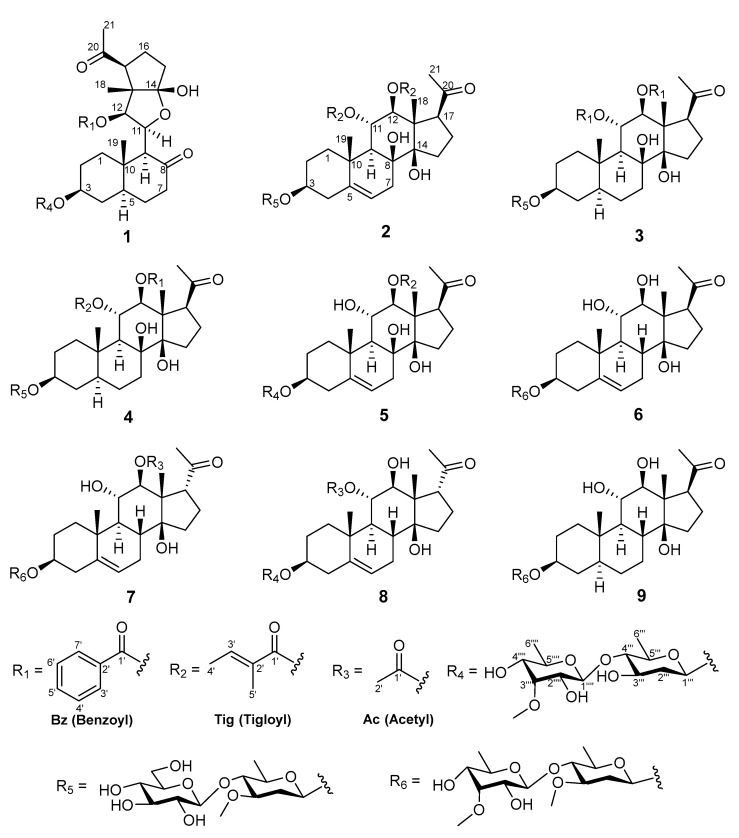
Structures of new compounds **1**–**9** from *Marsdenia tenacissima*.

*Marsdenia tenacissima* has been chemically investigated previously and is rich in polyoxygenated pregnane glycosides. So far, 166 polyoxygenated pregnane glycosides have been reported [7,8,9,10,11,12]. Many of them displayed a multidrug resistance reversal effect, antitumor effect, immunomodulatory activity, antiviral effect, and anti-angiogenic effect, and the components 11α-*O*-2-methylbutyryl-12β-*O*-2-tigloyltenacigenin B [9], 11α-*O*-2-methylbutyryl-12β-*O*-2-benzoyltenacigenin B [9], and 11α,12β-*O*,*O*-ditigloyl-17β-tenacigenin B [9] exhibited cytotoxicity against KB-VI cells, with ED_50_ values of 4.1, 2.5, and 3.4 μg/mL, respectively. In a continuation of efforts searching for biologically active natural products from traditional medicines, the ethyl acetate fraction of the ethanol extract of the stems of *M. tenacissima* was chemically investigated. As a result, nine new pregnane glycosides, marsdeosides A–I (**1**–**9**), have been isolated (Figure 1). Herein, the structure elucidation and inhibitory activities on nitric oxide production by lipopolysaccharide-activated macrophages (RAW264.7) of marsdeosides A–I (**1**–**9**) are described.

## 2. Results and Discussion

### 2.1. Structural Elucidation of Compounds ***1***–***9***

**Figure 2:**
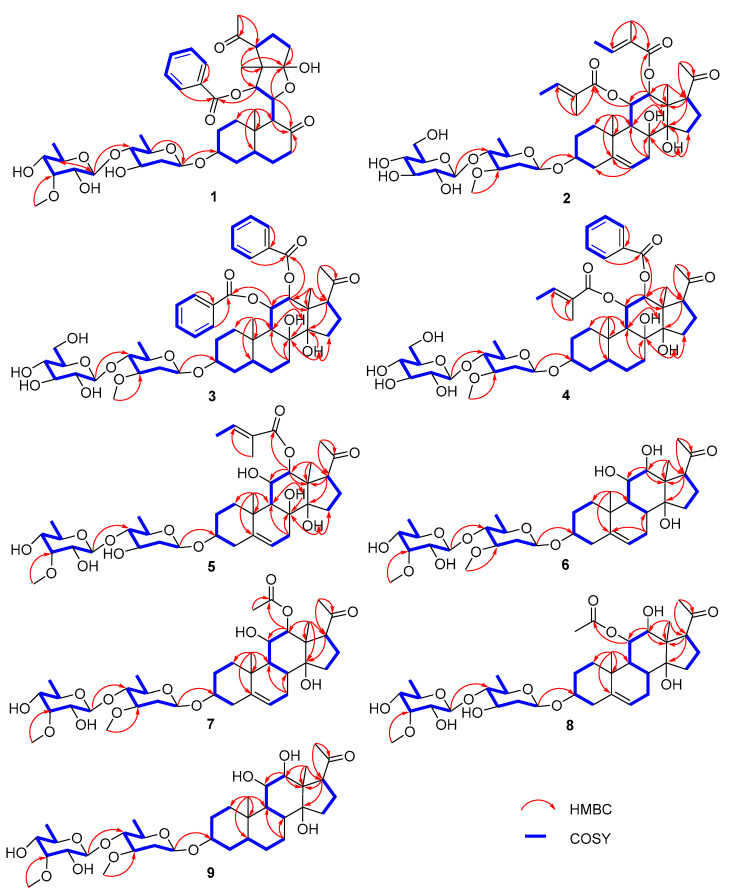
Selected HMBC and 1H–1H COSY correlations of compound **1**–**9**.

**Figure 3:**
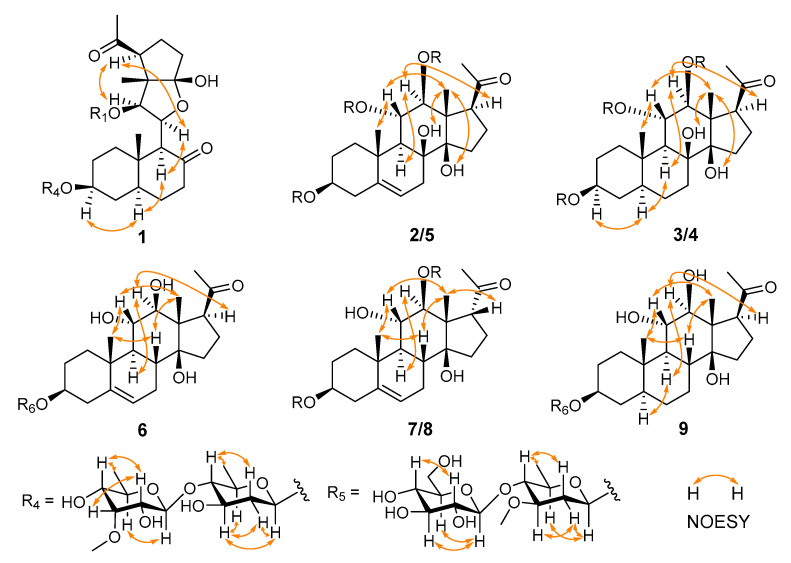
Selected NOESY correlations of compound **1**–**9**.

Compound **1**, obtained as white amorphous powders, had the molecular formula of C_41_H_58_O_14_, as determined by the sodium-adduct ion peak at *m*/*z* 797.37292 ([M + Na]^+^, calculated for C_41_H_58_O_14_Na, 797.37188) in HRESIMS analysis. The absorption bands for the hydroxy group (3411 cm^−1^), carbonyl group (1691 cm^−1^), and double-bond (1549 cm^−1^) groups were shown in the IR spectrum of **1**. The ^1^H NMR spectrum of **1** (Table 1 and Table 2; Figure S1, Supplementary data) showed 3 singlet methyls (*δ*_H_ 1.19, s, 3H; *δ*_H_ 0.89 s, 3H; *δ*_H_ 2.56 s, 3H), 2 doublet methyls (*δ*_H_ 1.67, d, *J* = 6.1 Hz; *δ*_H_ 1.49, d, *J* = 6.5 Hz), and 5 aromatic protons [*δ*_H_ 8.27 (2H, d, *J* = 8.4 Hz); 7.41 (2H, t, J = 8.4 Hz); 7.55 (1H, t, *J* = 8.4 Hz)]. The ^13^C NMR and DEPT spectroscopic data (Table 3; Figure S2, Supplementary data) of 1 displayed the signals for six methyls (one methoxy group), eight methylenes, twenty methines (including five olefinic/aromatic and thirteen oxygenated), and seven quaternary carbons (including two olefinic/aromatic and three carbonyls). The aforementioned data showed a similarity to those of periplocoside A [13], except for the presence of an additional carbonyl (*δ*_C_ 211.5), benzoyl group, and sp^3^ methyl group (*δ*_C_ 12.1); however, the absence of a dioxygenated quaternary carbon (*δ*_C_ 115.7) and the C-18 ester carbonyl in **1** indicated that **1** was a typical pregnane glycoside with a benzoyl group and 2 sugar moieties. The changes were confirmed by the key HMBC correlations (Figure 2) from H-6 (*δ*_H_ 1.46, m; 1.41, m), H-9 (*δ*_H_ 3.29, d, *J* = 10.0 Hz), and H-7 (*δ*_H_ 2.05; 2.22) to C-8 (*δ*_C_ 211.5); from H-18 (*δ*_H_ 1.19, s) to C-12 (*δ*_C_ 81.0), C-13 (*δ*_C_ 55.8), and the hemiketal carbon C-14 (*δ*_C_ 115.7); and from H-11 (*δ*_H_ 5.06) to C-14. These HMBC correlations also suggested that the bond between C-8 and C-14 was cleaved to form two carbonyls through oxidative cleavage of the C-8 and C-14 vicinal diol. The position of benzoxy residue was assigned at C-12 by the HMBC correlation from H-12 (*δ*_H_ 6.59) to the carbonyl group of Bz (C-1′, *δ*_C_ 165.9). The relative configuration of the aglycone part of **1** was established by the analysis of key correlations in the NOESY spectrum (Figure 3). The NOESY correlations of H-17/H-12, of H-9/H-5/H-3/H-1α, and of Me-19/H-1β suggested that H-3, H-9, H-12, and H-17 were in α-configuration, while Me-18 and Me-19 were in β-configuration.

Compound **1** had 2 sugar units, which was revealed by the existence of 2 anomeric protons at δ_H_ 4.87 (d, *J* = 9.3 Hz) and 5.18 (d, *J* = 8.3 Hz), which corresponded to the carbon resonances at *δ*_C_ 97.6 and 103.2, respectively, in the ^13^C NMR spectrum. The sugar chain was linked at C-3 according to the key HMBC correlation of H-3 (3.80 m) to the anomeric carbon (*δ*_C_ 97.6). In addition, the ^1^H NMR spectrum showed 2 methyl doublets at *δ*_H_ 1.67 (d, *J* = 6.1 Hz, 3H) and 1.49 (d, *J* = 6.5 Hz, 3H) and 1 methoxy group at *δ*_H_ 3.84, corresponding to the carbon signals at δ_C_ 18.3, 18.0, and 62.1, respectively. The ^1^H-^1^H COSY correlations of H-1′′′/H-2′′′/H-3′′′/H-4′′′/H-5′′′/Me-6′′′, together with the HMBC correlations of H-5′′′ (*δ*_H_ 3.68) to C-1′′′ (*δ*_C_ 97.6), enabled the assignment of the carbons of 1 sugar unit. The key NOESY correlations of H-1′′′/H-2′′′α (*δ*_H_ 2.57)/H-3′′′/H-5′′′ and of H-2′′′β (*δ*_H_ 2.03)/H-4 helped to assign the sugar as 3-*O*-demethyl-oleandropyranose. The ^1^H-^1^H COSY correlations of H-1′′′′/H-2′′′′/H-3′′′′/H-4′′′′/H-5′′′′/Me-6′′′′, along with the HMBC correlations from H-5′′′′ (*δ*_H_ 4.24) to C-1′′′′ (*δ*_C_ 103.2) and from the methoxy group (*δ*_H_ 3.84) to C-3′′′′, allowed the determination of the carbons of the other sugar unit, which was further determined as 6-deoxy-3-*O*-methyl-allopyranose by the diagnostic NOESY correlations of H-1′′′′/H-5′′′′,and of H-2′′′′/H-3′′′′/H-4′′′′/Me-6′′′′. The key HMBC correlations of H-1′′′′ to C-4′′′ revealed that the latter sugar unit was the terminal one, and the two sugar units were connected by the C-1′′′′ and C-4′′′. The absolute configurations of the sugar moieties were assigned by TLC analysis and comparing the optical rotation data of the hydrolysates with those of published data [14,15]. Two sugar units were finally identified as 6-deoxy-3-*O*-methyl-β-D-allopyranosyl-(1→4)-β-D-3-*O*-demethyl-oleandropyranose by comparison of the *R*_f_ data and optical rotations, respectively. Thus, the structure of compound **1** was defined to be marsdeoside A.

Compound **2** was isolated as a white amorphous powder. The absorption bands for the hydroxy group (3406 cm^−1^), carbonyl group (1689 cm^−1^), and double bond (1446 cm^−1^) were shown in the IR spectrum. It had the molecular formula of C_44_H_66_O_16_, as determined by the sodium-adduct ion peak at *m*/*z* 873.42303 ([M + Na]^+^, calculated for C_44_H_66_O_16_Na, 873.42431) in the HRESIMS analysis. The 1D NMR spectroscopic data of **2** (Table 1, Table 2 and Table 3; Figures S8 and S9, Supplementary data) were similar to iloneoside (3-*O*-[6-deoxy-3-*O*-methyl-β-dallopyranosyl-(1→14)-β-D-oleandropyranosyl]-11,12-di-*O*-tigloyl-17β-marsdenin) [16], except for the species of the terminal sugar unit. By analysis of the 2D NMR data of 2, the presence of a glucose moiety was confirmed by the key ^1^H-^1^H COSY correlations (Figure 2) of H-1′′′′/H-2′′′′/H-3′′′′/H-4′′′′/H-5′′′′/H-6′′′′ and NOESY correlations (Figure 3) of H-5′′′′/H-1′′′′/H-3′′′′, and H-2′′′′/H-4′′′′. The key HMBC (Figure 2) correlation from H-1′′′′ to C-4′′′ of the oleandropyranosyl suggested a (1→4) glycosidic linkage. The absolute configurations of the sugars were determined as follows. Compound **2** was subjected to acidic hydrolysis, and the side chain sugars were identified as D-oleandropyranose (Ole) and D-glucose (Glc) by comparing the *R*_f_ values and optical rotations to the literature data [8,17]. Thus, the structure of compound **2** was defined to be marsdeoside B.

The molecular formula of compound **3** was determined to be C_48_H_64_O_16_ by HRESIMS *m*/*z* 919.40680 ([M + Na]^+^, calculated for C_48_H_64_O_16_Na, 919.40866). The absorption bands for the hydroxy group (3402 cm^−1^), carbonyl group (1720 cm^−1^), and double bond (1450 cm^−1^) were shown in the IR spectrum. The 1D NMR spectroscopic data (Table 1, Table 2 and Table 3; Figures S15 and S16, Supplementary data) showed that **3** was also a pregnane glycoside with a bi-sugar moiety. With the careful comparison of NMR chemical shifts with compound **2** and the literature data, it was found that the bi-sugar moiety was the same as compound **2**, while the aglycone moiety, including the absolute configuration, was identical with that of the compound 3-*O*-[6-deoxy-3-*O*-methyl-β-allopyanosyl-(1→4)-β-digitoxopyranoside]-11α,12β-di-*O*-benzoyl-17β-marsdenin-5,6-dihydrogen (Compound 3 in [18]). Further elucidation of the 2D NMR spectra confirmed the above assignments. By comparison of the *R*_f_ values and optical rotations of the acidic-hydrolyzed sugars of compound **3**, the sugars were identified as D-oleandrose and D-glucose [8,13,17]. Thus, the structure of compound **3** was defined to be marsdeoside C.

Compound **4** had a molecular formula determined to be C_48_H_66_O_16_ by HRESIMS *m*/*z* 897.42218 ([M + Na]^+^, calculated for C_46_H_66_O_16_Na, 897.42431). The IR spectrum displayed absorption bands for the hydroxy group (3413 cm^−1^), carbonyl group (1691 cm^−1^), and double bonds (1450 cm^−1^). The 1D NMR spectroscopic data (Table 1, Table 2 and Table 3; Figures S22 and S23, Supplementary data) showed that **4** was also a pregnane glycoside with a bi-sugar moiety, which was indicated by the anomeric protons at *δ*_H_ 5.16 (d, *J* = 7.8 Hz) and 4.87 (d, *J* = 9.5 Hz), corresponding to the carbon resonances at *δ*_C_ 97.4 and 104.4 in the 1D NMR spectrum. Comparing the chemical shifts with those of compound **3**, it was found that the composition and connection of bi-sugar moieties were the same as **3**. However, the modifying moieties of the aglycone part were different between compounds **3** and **4**. In compound **4**, the 12-O was attached to a benzoyl group, instead of being attached to a tigolyl group as in compound **3**. This change was corroborated by the key HMBC correlation from H-12 (δ_H_ 5.62) to C-1′′ (*δ*_C_ 166.8) and the ^1^H-^1^H COSY correlations of H-3′′/H-4′′/H-5′′/H-6′′/H-7′′ (Figure 2). By comparison of the Rf and optical rotations of the sugars that hydrolyzed from **4** in an acidic condition, the sugars were identified as D-oleandrose and D-glucose [8,13,17]. Therefore, the structure of compound **4** was defined to be marsdeoside D.

The molecular formula of compound **5** was determined to be C_39_H_60_O_14_ by HRESIMS *m*/*z* 775.38599 ([M + Na]^+^, calculated for C_39_H_60_O_14_Na, 775.38753). The IR spectrum displayed absorption bands for the hydroxy group (3410 cm^−1^), carbonyl group (1685 cm^−1^), and double bonds (1446 cm^−1^). The 1D NMR spectroscopic data (Table 1, Table 2 and Table 3; Figures S29 and S30, Supplementary data) showed that **5** was a pregnane glycoside with a bi-sugar moiety. Comparing the NMR data with those of compounds **1** and **2**, it was found that compound **5** also harbored a 6-deoxy-3-*O*-methyl-β-D-allopyranosyl-(1→4)-β-D-3-*O*-demethyl-oleandropyranosyl sugar moiety, which was identical with that of **1**. The aglycone part of **5** also exhibits a high similarity with its counterpart in compound **2**, except for the absence of a tigolyl group of the 11-OH. The presence of exchangeable protons at *δ*_H_ 6.49 (d, *J* = 6.4 Hz) and the diagnostic HMBC correlations (Figure 2) from this exchangeable proton to C-9 (*δ*_C_ 50.4), C-11 (*δ*_C_ 68.5) and C-12 (*δ*_C_ 81.1) suggested that the 11-OH was free in **5** instead of substituted by a tigolyl group. The configurations of the aglycone and sugars were determined to be consistent with the corresponding parts of compounds **1** and **2**. Thus, compound **5** was defined to be marsdeoside E.

Compound **6** had a molecular formula of C_35_H_56_O_12_ that was determined by HRESIMS *m*/*z* 691.36763 ([M + Na]^+^, calculated for C_35_H_56_O_12_Na, 691.36640). The IR spectrum displayed absorption bands for the hydroxy group (3446 cm^−1^), carbonyl group (1693 cm^−1^), and double bond (1548 cm^−1^). The 1D NMR spectroscopic data (Table 1, Table 2 and Table 3; Figures S36 and S37, Supplementary data) showed that **6** was also a pregnane glycoside with a bi-sugar unit. The key 1H-1H COSY correlations (Figure 2) of H-1′′′/H-2′′′/H-3′′′/H-4′′′/H-5′′′/Me-6′′′ and H-1′′′′/H-2′′′′/H-3′′′′/H-4′′′′/H-5′′′′/Me-6′′′′, together with the HMBC correlations (Figure 2) from one methoxy to C-3′′′, from the other methoxy to C-3′′′′, and from H-1′′′′ to C-4′′′, suggested the presence of an oleandrose and a terminal 6-deoxy-3-*O*-methylallose, which was connected by a (1→4) glycosidic linkage.

Furthermore, the aglycone was further elucidated by analysis of the HSQC, HMBC, and ^1^H-^1^H COSY correlations and comparison of the NMR data with those of compounds **2**–**5**. Except for the carbons that were already assigned to the bi-sugar moiety, only 21 carbons remained, which suggested the nonexistence of any modification groups of the aglycone part. Furthermore, a comparison of the chemical shifts of the remaining 21 carbons with those of the core steroidal skeleton of **5** revealed that the only difference was the oxygenated status of C-8. The key ^1^H-^1^H COSY correlations of H-7/H-8/H-9 demonstrated that C-8 was a methine instead of an oxygenated quaternary carbon as in **5**. Analysis of the NOESY spectrum suggested the β orientation of H-8 by the key NOESY correlations of H-8/H-11/Me-18. The configurations of the other chiral carbons of 6 were identical with those of compound **5**. The sugars were determined to be 6-deoxy-3-*O*-methyl-β-D-allopyranose and β-D-oleandropyranose by comparing the physical data with the authenticate samples. Thus, the structure of compound 6 was identified to be marsdeoside F.

Compound **7** was obtained as a white amorphous powder. It had a molecular formula of C_37_H_58_O_13_, as determined by *m*/*z* 733.37496 ([M + Na]^+^, calculated for C_37_H_58_O_13_Na, 733.37696). The 1D NMR spectra (Table 1, Table 2 and Table 3; Figures S43 and S44, Supplementary data) of **7** exhibited a high similarity to that of **6**, except for the existence of a carbonyl (*δ*_C_ 170.4) and a methyl singlet (*δ*_H_ 1.98, s; *δ*_C_ 21.3) in **7**, corresponding to an acetyl group. Analysis of the 2D NMR spectra enabled the assignment of the acetyl group at the 12-O position by the key HMBC correlation (Figure 2) of H-12 (*δ*_H_ 5.32) to C-1′ (*δ*_C_ 170.4). The pivotal NOESY correlations between Me-18 (*δ*_H_ 1.74)/H-17 (*δ*_H_ 3.57) indicated that the H-17 was in β orientation, which was different from compounds **1**–**6**. Therefore, compound **7** was assigned to be marsdeoside G.

Compound **8** had a molecular formula of C_36_H_56_O_13_ by HRESIMS analysis. The 1D NMR data (Table 1, Table 2 and Table 3; Figures S50 and S51, Supplementary data) of **8** were highly similar to those of compound **7**. The differences between these two compounds were the sugar species and the location of the acetyl group. Elucidation of the 2D NMR spectra suggested that the sugar part of **8** was consistent with that of compounds 1 and 5. However, the key HMBC correlation (Figure 2) from H-11 (*δ*_H_ 5.68) to the acetyl carbonyl group at *δ*_C_ 170.9, C-9 (*δ*_C_ 48.5), C-10 (*δ*_C_ 39.7), and C-12 (*δ*_C_ 72.4) revealed that the acetoxy group was located at C-11 in 8. The key NOESY correlations between Me-18 (*δ*_H_ 1.79)/H-17 (*δ*_H_ 3.49) indicated that the H-17 was in β orientation. Therefore, compound **8** was identified as marsdeoside H.

Compound **9** had a molecular formula of C_35_H_58_O_12_, as determined by HRESIMS with the sodium-adduct ion peak at m/z 693.38004 ([M + Na]^+^, calculated for C_35_H_58_O_12_Na, 693.38205). The 1D NMR spectra (Table 1, Table 2 and Table 3; Figures S57 and S58, Supplementary data) of **9** were highly similar to those of **6**, except for the absence of double-bond data in **9**. The key ^1^H-^1^H COSY correlations (Figure 2) between H-4/H-5/H-6/H-7 suggested that C-5 was a methine and C-6 was a methylene in compound **9**. The key NOESY correlations of H-12/H-9/H-5/H-3 suggested the α orientation of H-5. Further analysis of the 2D NMR data suggested that the configurations of the other chiral carbons were the same as those of compound **6**. Thus, compound **9** was assigned to be marsdeoside I.

### 2.2. Inhibitory Activities of Compounds on NO Production by Lipopolysaccharide-Activated Macrophage (RAW264.7)

Based on the clinical applications and previous biological studies on the secondary metabolites of this plant, the isolates in this study were subjected to anti-inflammatory activity assays. Nine compounds were evaluated for their inhibitory activities on nitric oxide production by a lipopolysaccharide-activated macrophage (RAW264.7). The *N*^G^-monomethyl-L-arginine (L-NMMA) was used as a positive control. As shown in Table 4, compounds **1**, **8**, and **9** showed anti-inflammatory activities, with IC_50_ values of 37.5, 38.8, and 42.8 μM, respectively, which were comparable to the positive control.

## 3. Experimental Section

### 3.1. General Experimental Procedures

High-resolution electrospray ionization mass spectra (HRESIMS) were measured with a Q Exactive Orbitrap mass spectrometer (Thermo Fisher Scientific, Waltham, MA, USA). The instrumental parameters for HRESIMS analysis were as follows: capillary temperature 325 °C, spray voltage 3.80 kV, sheath gas flow rate 40 mL/min, auxiliary gas flow rate 20 mL/min. Medium pressure liquid chromatography (MPLC) was performed on an Interchim system equipped with a column packed with Chromatorex C_18_ gel (40–75 μm, Fuji Silysia Chemical Ltd., Kasugai, Japan). UV spectra were recorded on a Hitachi UH5300 spectrophotometer (Hitachi, Tokyo, Japan). The NMR spectra (^1^H, ^13^C, and 2D NMR) were measured on Bruker Avance III NMR instruments at 600 MHz for ^1^H and 150 MHz for ^13^C NMR. IR spectra were obtained in a Tenor 27 spectrophotometer (Bio-Rad, Richmond, CA, USA) using KBr pellets. Column chromatography (CC) was executed on silica gel (80–100 mesh or 200−300 mesh, Qingdao Haiyang Chemical Co., Ltd., Qingdao, China), Sephadex LH20 (Pharmacia Fine Chemical Co., Ltd., Uppsala, Sweden), and Reverse-phase silica gel (20−45 μm, Fuji Silysia Chemical Ltd.). Medium-pressure liquid chromatography (MPLC) was applied to Biotage SP2 equipment, and columns were packed with reverse-phase silica gel (C_18_). Preparative high-performance liquid chromatography (prep-HPLC) was performed on an Agilent 1260 liquid chromatography system equipped with YMC-Pack ODS-A columns (S-5 μm, 250 × 10 mm) and a DAD detector (Agilent Technologies, Santa Clara, CA, USA). All fractions were monitored by thin-layer chromatography (TLC) (Qingdao Haiyang Chemical Co., Ltd., Qingdao, China). Preparative TLC (silica gel 60, Merck KGaA, 64271 Darmstadt) was used to purify the acid-hydrolyzed sugars. The spots were visualized by heating silica gel plates soaked with a vanillin–sulfuric acid color component solvent.

### 3.2. Plant Material

The stems of *Marsdenia tenacissima* were collected before flowering from Baoshan County, Yunnan Province, People’s Republic of China, in May 2021. The plant was identified by Dr. Hong-Lian Ai (Associate Professor of South-Central Minzu University, Wuhan, Hubei 430074, China). A voucher specimen (2021103FD) was deposited in the School of Pharmaceutical Sciences, South-Central Minzu University. The stems were dried and then stored at 4 °C until extraction.

### 3.3. Extraction and Isolation

The dried stems of *Marsdenia tenacissima* (1.3 kg) were mechanically crushed and percolated with EtOH/H_2_O (95:5) at room temperature for cell rupture by water absorption. After filtration, the samples were extracted exhaustively with dichloromethane/methanol (1:1, *v*/*v*; 5 L × 4) at room temperature. The solvent was evaporated in vacuo to give a dark gum (89 g), which was dissolved in water (1 L) and then extracted with ethyl acetate (2 L × 4) to give ethyl acetate extract parts (36 g). The ethyl acetate extract parts were dissolved in dichloromethane and then placed on a silica gel column eluted with dichloromethane containing increasing amounts of methanol. Five fractions (BE-1/BE-2/BE-3/BE-4/BE-5) were collected. Among them, the last fraction was eluted with methanol. Fraction BE-3 (20.0 g) was subjected to ODS silica gel CC and eluted with MeOH−H_2_O (10:90→100:1, *v*/*v*) to yield 11 fractions (Frs. 3-3-1→3-3-11). Fr. 3-3-2 was purified by preparative HPLC with CH_3_CN−H_2_O (20:80→40:60, *v*/*v*) to give **1** (5.0 mg, t_R_ = 6.5 min, 0.14 ‰ of yield) and **5** (20.0 mg, t_R_ = 21 min, 0.56 ‰ of yield). Fr. 3-3-3 was purified by semi-preparative HPLC with CH_3_CN−H_2_O (35:65→53:47, *v*/*v*) to give **2** (18 mg, 0.5 ‰ of yield), **4** (22 mg, 0.61 ‰ of yield), and **3** (16 mg, 0.44 ‰ of yield) at 22 min, 27 min, and 29 min, respectively. Fr. 3-3-10 was also treated by preparative HPLC (CH_3_CN−H_2_O, 20:50→50:50, *v*/*v*) and yielded **6** (4.3 mg, 0.12 ‰ of yield) and **7** (3.0 mg, 0.08 ‰ of yield) at 20 min and 17 min, respectively. Fr. 3-3-11 was purified by preparative HPLC with CH_3_CN−H_2_O (20:80→28:72, *v*/*v*) to give **8** (4.0 mg, 0.11 ‰ of yield) and **9** (6.5 mg, 0.18 ‰ of yield) at 10.5 min and 15 min, respectively.

#### 3.3.1. Marsdeoside A (**1**)

White amorphous powder; ${\left[\alpha \right]}_{D}^{26}$ −96.6 (*c* = 0.09, MeOH); UV (MeOH) λ_max_ (log ε): 250 (3.10) nm; IR (KBr) ν_max_ 3411, 2935, 1691, 1549, 1441, 1305, 1164, and 1031 cm^−1^; HRESIMS *m*/*z* 797.37292 ([M + Na]^+^, calculated for C_41_H_58_O_14_Na, 797.37188). ^1^H and ^13^C NMR data displayed in Table 1 and Table 2.

#### 3.3.2. Marsdeoside B (**2**)

White amorphous powder; ${\left[\alpha \right]}_{D}^{26}$ −56.8 (*c* = 0.09, MeOH); UV (MeOH) λ_max_ (log ε): 250 (3.08) nm; IR (KBr) ν_max_ 3406, 2935, 1689, 1446, 1377, 1220, 1104, 1049, and 1037 cm^−1^; HRESIMS *m*/*z* 873.42303 ([M + Na]^+^, calculated for C_44_H_66_O_16_Na, 873.42431). ^1^H and ^13^C NMR data displayed in Table 1 and Table 2.

#### 3.3.3. Marsdeoside C (**3**)

White amorphous powder; ${\left[\alpha \right]}_{D}^{22}$ −27.0 (*c* = 0.12, MeOH); UV (MeOH) λ_max_ (log ε): 250 (3.27) nm; IR (KBr) ν_max_ 3402, 2935, 1720, 1450, 1387, 1276, 1095, and 1068 cm^−1^; HRESIMS C_48_H_64_O_16_ by HRESIMS *m*/*z* 919.40680 ([M + Na]^+^, calculated for C_48_H_64_O_16_Na, 919.40866). ^1^H and ^13^C NMR data displayed in Table 1 and Table 2.

#### 3.3.4. Marsdeoside D (**4**)

White amorphous powder; ${\left[\alpha \right]}_{D}^{20}$ +98.3 (*c* = 0.11, MeOH); UV (MeOH) λ_max_ (log ε): 250 (3.23) nm; IR (KBr) ν_max_ 3413, 2931, 1691, 1450, 1373, 1164, 1130, and 1082 cm^−1^; HRESIMS *m*/*z* 897.42218 ([M + Na]^+^, calculated for C_46_H_66_O_16_Na, 897.42431). ^1^H and ^13^C NMR data displayed in Table 1 and Table 2.

#### 3.3.5. Marsdeoside E (**5**)

White amorphous powder; ${\left[\alpha \right]}_{D}^{26}$ +61.1 (*c* = 0.09, MeOH); UV (MeOH) λ_max_ (log ε): 250 (3.08) nm; IR (KBr) ν_max_ 3410, 2935, 1685, 1446, 1377, 1165, 1128, and 1064 cm^−1^; HRESIMS *m*/*z* 775.38599 ([M + Na]^+^, calculated for C_39_H_60_O_14_Na, 775.38753). ^1^H and ^13^C NMR data displayed in Table 1 and Table 2.

#### 3.3.6. Marsdeoside F (**6**)

White amorphous powder; ${\left[\alpha \right]}_{D}^{26}$ −35.6 (*c* = 0.11, MeOH); UV (MeOH) λ_max_ (log ε): 250 (0.74) nm; IR (KBr) ν_max_ 3446, 2935, 1693, 1548, 1377, 1165, 1126, and 1064 cm^−1^; HRESIMS *m*/*z* 691.36763 ([M + Na]^+^, calculated for C_35_H_56_O_12_Na, 691.36640). ^1^H NMR and ^13^C NMR data displayed in Table 1 and Table 2.

#### 3.3.7. Marsdeoside G (**7**)

White amorphous powder; ${\left[\alpha \right]}_{D}^{26}$ +23.4 (*c* = 0.10, MeOH); UV (MeOH) λ_max_ (log ε): 250 (3.07) nm; IR (KBr) ν_max_ 3410, 2935, 1689, 1446, 1377, 1165, 1125, and 1067 cm^−1^; HRESIMS *m*/*z* 733.37496 ([M + Na]^+^, calculated for C_37_H_58_O_13_Na, 733.37696). ^1^H and ^13^C NMR data displayed in Table 1 and Table 2.

#### 3.3.8. Marsdeoside H (**8**)

White amorphous powder; ${\left[\alpha \right]}_{D}^{27}$ −88.5 (*c* = 0.11, MeOH); UV (MeOH) λ_max_ (log ε): 250 (3.07) nm; IR (KBr) ν_max_ 3421, 2947, 1651, 1492, 1377, 1165, and 1024 cm^−1^; HRESIMS *m*/*z* 719.35931 ([M + Na]^+^, calculated for C_36_H_56_O_13_Na, 719.36131). ^1^H and ^13^C NMR data displayed in Table 1 and Table 2.

#### 3.3.9. Marsdeoside I (**9**)

White amorphous powder; ${\left[\alpha \right]}_{D}^{27}$ −96.6 (*c* = 0.11, MeOH); UV (MeOH) λ_max_ (log ε): 250 (0.615) nm; IR (KBr) ν_max_ 3421, 2930, 1651, 1548, 1492, 1374, 1304, and 1163 cm^−1^; HRESIMS *m*/*z* 693.38004 ([M + Na]^+^, calculated for C_35_H_58_O_12_Na, 693.38205). ^1^H and ^13^C NMR data displayed in Table 1 and Table 2.

### 3.4. Acidic Hydrolysis of Compounds ***1***–***9***

Compounds **1**–**9** (each 2.0 mg) were dissolved in 2 M HCl (1,4-dioxane/H_2_O, 1:1 *v*/*v*, 1 mL). The solution was kept at 60 °C for 2 h and then was attenuated with H_2_O (3 mL). The hydrolyzed mixture was extracted with CH_2_Cl_2_ (4 mL × 3). The sugars were detected by TLC and compared to the standard compounds. The four sugars were confirmed as glucose, oleandrose, 3-*O*-demethyl-oleandrose, and 6-deoxy-3-*O*-methyl-allose, respectively, on the basis of the R_f_ values. The sugars were purified by preparative TLC and were subjected to measurement of the specific optical rotation values. Moreover, as a result, 3-*O*-demethyl-oleandrose was detected in the glycosides **1**, **5**, and **8**; 6-deoxy-3-*O*-methyl-allose was detected from **1**, **5**–**9**; glucose was detected from **2**–**4**; and oleandrose was detected in **2**–**4**, **6**, **7**, and **9**. In addition, monosaccharides of compounds **1**–**9** were all considered to be D-form by comparing their optical rotation (OR) with those reported in the literature [8,14,17].

### 3.5. Anti-Inflammatory Activity Assays

The murine mononuclear macrophages RAW264.7 were seeded into 96-well plates and stimulated with 1 μg/mL lipopolysaccharide (LPS). At the same time, the compounds with different concentrations were added. The drug-free group and the L-NMMA-positive drug group were set approximately equal as a comparison. After the cells were cultured overnight, the medium was taken to detect the production of nitric oxide (NO), and the absorbance was measured at 570 nm. MTS was added to the remaining medium for cell viability assays to exclude the toxic effects of compounds on cells. The assays were carried out as a triplicate batch of experiments. The NO production inhibition rate (%) = [(OD_570 nm_ of non-drug treatment group − OD_570 nm_ of sample group)/OD_570 nm_ of non-drug treatment group] × 100%. IC_50_ (50% concentration of inhibition) was calculated by the Reed & Muench method [19,20].

### 3.6. Determination of the Absolute Configuration of the Monosaccharides

The optical rotations of 6-deoxy-3-*O*-methyl-D-allose, D-oleandrose, 3-*O*-demethyl-D-oleandrose, and D-glucose in the literature were ${\left[\alpha \right]}_{D}^{20}$ +10.0, ${\left[\alpha \right]}_{D}^{20}$ −12.0, ${\left[a\right]}_{D}^{20}$ +43.0, and ${\left[a\right]}_{D}^{20}$ +48.0, respectively [8,14,17]. The optical rotations of 6-deoxy-3-*O*-methyl-allose and 3-*O*-demethyl-D-oleandrose were ${\left[\alpha \right]}_{D}^{20}$ +10.2 (*c* = 0.17, H_2_O) and ${\left[a\right]}_{D}^{20}$ +43.5 (*c* = 0.20, H_2_O), respectively, in **1**; the optical rotation of oleandrose ${\left[\alpha \right]}_{D}^{20}$ −12.3 and glucose ${\left[a\right]}_{D}^{20}$ +49.3 (*c* = 0.20, H_2_O) in **2**; the optical rotation of oleandrose ${\left[\alpha \right]}_{D}^{20}$ −12.3 (*c* = 0.21, H_2_O) and glucose ${\left[a\right]}_{D}^{20}$ +48.3 (*c* = 0.20, H_2_O) in **3**; the optical rotation of oleandrose ${\left[\alpha \right]}_{D}^{20}$ −12.1(*c* = 0.19, H_2_O) and glucose ${\left[a\right]}_{D}^{20}$ +49.0 (*c* = 0.20, H_2_O) in **4**; the optical rotation of 6-deoxy-3-*O*-methyl-allose ${\left[\alpha \right]}_{D}^{20}$ +10.2 (c = 0.17, H_2_O) and 3-*O*-demethyl-D-oleandrose ${\left[a\right]}_{D}^{20}$ +49.0 (*c* = 0.20, H_2_O) in **5**; the optical rotation of 6-deoxy-3-*O*-methyl-allose ${\left[\alpha \right]}_{D}^{20}$ +10.2 (*c* = 0.17, H_2_O) and oleandrose ${\left[\alpha \right]}_{D}^{20}$ −12.6 (*c* = 0.22, H_2_O) in **6**; the optical rotation of 6-deoxy-3-*O*-methyl-allose ${\left[\alpha \right]}_{D}^{20}$ +10.3 (c = 0.18, H_2_O) and oleandrose ${\left[\alpha \right]}_{D}^{20}$ −12.2 (*c* = 0.22, H_2_O) in **7**; the optical rotation of 6-deoxy-3-*O*-methyl-allose ${\left[\alpha \right]}_{D}^{20}$ +10.3 (*c* = 0.18, H_2_O) and 3-*O*-demethyl-D-oleandrose ${\left[a\right]}_{D}^{20}$ +43.6 (*c* = 0.19, H_2_O) in **8**; and the optical rotation of 6-deoxy-3-*O*-methyl-allose ${\left[\alpha \right]}_{D}^{20}$ +10.2 (*c* = 0.17, H_2_O) and oleandrose ${\left[\alpha \right]}_{D}^{20}$ −12.6 (*c* = 0.22, H_2_O) in **9**. Thus, monosaccharides of compounds **1**–**9** were all considered to be D-form by comparing their specific optical rotations with those reported in the literature [8,14,17].

## 4. Conclusions

This phytochemical study of the cultivated medicinal plant *Marsdenia tenacissima* led to the isolation and structural elucidation of nine new pregnane glycosides (Figure 1), including one new 8,14-seco-pregnane glycoside (**1**) harboring a new hemiketal formed at the C-11 and C-8 positions. All of the undescribed pregnane glycosides were evaluated for inhibitory activity against nitric oxide production by a lipopolysaccharide-stimulated macrophage (RAW264.7), and three compounds showed comparable inhibitory activity to the positive control in vitro (Table 4). The structures and in vitro anti-inflammatory activity of these nine new pregnane glycosides were reported for the first time. This study broadens the horizon of the structural diversity of preganane glycosides of *Marsdenia tenacissima*, but also provides new evidence for the clinical applications of the botanical drug of this plant. Folk and ethnic medicines are of great importance and are valuable reservoirs for lead compounds in the field of drug research and development. The deeper the understanding of the chemistry of a medicinal plant, the better the way the sustainable utilization of the plant resources will be adopted.

## Figures and Tables

**Table 1:** ^1^H NMR Spectroscopic Data of the Aglycones of Compounds **1**–**9** in Pyridine-*d*_5_ (600 MHz, *J* in Hz).

No.	1	2	3	4	5	6	7	8	9
1	2.51, overlapped 1.69, overlapped	2.47, dt (13.5, 3.5) 1.43, td (13.5, 3.5)	2.27, dt (13.5, 3.8) 1.36, td (13.5, 3.8)	2.24, dt (13.2, 4.0) 1.29, td (13.2, 4.3)	2.62, overlapped 2.63, overlapped	2.60, br. d (12.0) 2.46, overlapped	2.61, br. d (12.0) 2.47, overlapped	2.58, overlapped 2.40, br. t (12.0)	3.14, br. d (14.0) 1.46, br. t (14.0)
2	1.99, m 1.58, m	2.12, overlapped 1.92, overlapped	1.86, overlapped 1.65, ddd (13.5, 13.5, 13.5)	2.03, m 1.77, overlapped	2.13, m 1.99, overlapped	2.11, m 1.86, m	2.14, m 1.85, overlapped	2.17, overlapped 1.83, overlapped	2.10, overlapped 1.74, overlapped
3	3.80, m	3.89, m	3.91, m	3.94, m	3.93, m	3.86, m	3.87, m	3.83, m	3.91, m
4	1.73, m 1.22, br. t (12.2)	2.63, dd (13.0, 4.5) 2.56, t (13.0)	1.84, overlapped 1.53, overlapped	1.83, br. d (12.0) 1.53, overlapped	3.35, br. d (13.6) 1.44, td (13.6, 3.8)	3.25, br. d (14.3) 1.43, td (14.8, 3.0)	3.26, br. d (13.9) 1.48, td (14.3, 3.6)	2.16, overlapped 1.41, td (12.6, 3.6)	1.77, overlapped 1.39, overlapped
5	1.57, m		1.23, m	1.19, m					1.11, m
6	1.46, m 1.41, m	5.44, d (5.5)	1.96, ddd (13.0, 13.0, 13.0) 1.19, m	1.93, m 1.16, overlapped	5.41, d (5.1)	5.55, overlapped	5.57, d (5.6)	5.55, d (5.4)	2.50, br .d (12.1) 1.16, overlapped
7	2.22, m 2.05, br. d (12.0)	2.66, dd (18.0, 5.5) 2.39, br. d (18.0)	2.41, overlapped 1.56, overlapped	2.37, m 1.52, overlapped	2.62, m 2.29, m	2.67, br. d (17.0) 1.97, m	2.66, overlapped 1.98, overlapped	2.70, overlapped 2.07, overlapped	1.28, overlapped 1.21, overlapped
8						2.03, overlapped	2.14, overlapped	2.21, td (11.7, 4.7)	1.92, overlapped
9	3.29, d (10.0)	2.27, d (10.5)	2.11, d (11.0)	1.97, d (11.0)	1.99, overlapped	1.61, t (11.0)	1.73, t (9.7)	1.79, overlapped	1.31, overlapped
11	5.06, overlapped	6.47, br. t (10.5)	6.82, t (10.6)	6.63, t (10.7)	4.89, td (10.7, 6.4)	4.10, overlapped	4.19, overlapped	5.68, t (9.9)	3.96, t (9.7)
12	6.59, d (4.7)	5.50, d (10.5)	5.76, d (10.6)	5.62, d (10.0)	5.49, d (9.7)	3.56, overlapped	5.32, d (9.7)	3.90, d (9.6)	3.48, d (9.5)
15	2.21, m, 2H	2.29, overlapped2.09, m	2.47, overlapped2.17, m	2.40, overlapped 2.13, m	2.19, m 2.04, m	2.06, overlapped 1.94, overlapped	2.09, overlapped 1.86, overlapped	2.11, overlapped 1.95, overlapped	2.07, overlapped 1.96, overlapped
16	2.47, m 1.80, m	2.33, m 2.07, m	2.33, m 2.17, m	2.31, m 2.14, m	2.28, m 2.05, m	2.22, m 1.86, overlapped	2.68, overlapped 1.87, overlapped	2.71, overlapped 1.99, overlapped	2.26, m 1.86, m
17	3.25, br. t (8.0)	3.22, dd (9.0, 5.0)	3.37, dd (9.8, 5.7)	3.36, dd (9.3, 5.5)	2.21, overlapped	3.88, overlapped	3.57, overlapped	3.49 br. t (8.8)	3.83, dd (8.8, 5.2)
18	1.19, s, 3H	1.68, s, 3H	1.79, s, 3H	1.74, s, 3H	1.64, s, 3H	1.35, s	1.74, s, 3H	1.79, s, 3H	1.36, s, 3H
19	0.89, s, 3H	1.72, s, 3H	1.58, s, 3H	1.55, s, 3H	1.82, s, 3H	1.39, s	1.33, s, 3H	1.33, s, 3H	1.01, s, 3H
21	2.56, s, 3H	2.20, s, 3H	2.07, s, 3H	2.06, s, 3H	2.21, s, 3H	2.30, s	2.28, s, 3H	2.37, s, 3H	2.31, s, 3H
8-OH		5.30, s		4.90, s	4.77, s				
11-OH					6.49, d (6.4)				
14-OH	8.16, s	5.82, s	6.00, s	5.96, s	5.60, s				

**Table 2:** ^1^H NMR Spectroscopic Data of the Decorating Groups of Compounds **1**–**9** in Pyridine-*d*_5_ (600 MHz, *J* in Hz).

	1	2	3	4	5	6	7	8	9
**Substituents**	**11-Benzoyl**	**11-Tigloyl**	**11-Benzoyl**	**11-Tigloyl**				**11-Acetyl**	
1′									
2′								1.96, s, 3H	
3′	8.27, d (8.4)	7.01, q (7.2)	8.05, d (8.4)	6.84, q (7.1)					
4′	7.41, t (8.4)	1.61, d (7.2), 3H	7.23, t (8.4)	1.39, d (7.1)					
5′	7.55, t (8.4)	1.84, s, 3H	7.31, t (8.4)	1.55, s, 3H					
6′	7.41, t (8.4)		7.23, t (8.4)						
7′	8.27, d (8.4)		8.05, d (8.4)						
		**12-Tigloyl**	**12-Benzoyl**	**12-Benzoyl**	**12-Tigloyl**		**12-Acetyl**		
1′′									
2′′							1.98, s, 3H		
3′′		7.10, q (7.1)	8.10, d (8.4)	8.28, d (8.4)	7.03, q (7.1)				
4′′		1.64, d (7.1), 3H	7.25, t (8.4)	7.42, t (8.4)	1.56, d (7.1)				
5′′		1.91, s, 3H	7.36 t (8.4)	7.52, t (8.4)	1.85, s, 3H				
6′′			7.25, t (8.4)	7.42, t (8.4)					
7′′			8.10, d (8.4)	8.28, d (84)					
**Sugar moieties**	**3-*O*-deMe-D-Ole**	**D-Ole**	**D-Ole**	**D-Ole**	**3-*O*-deMe-D-Ole**	**D-Ole**	**D-Ole**	**3-*O*-deMe-D-Ole**	**D-Ole**
1′′′	4.87, br. d (9.3)	4.81, br. d (9.9)	4.84, d (9.6)	4.87, d (9.5)	4.93, d (9.7)	4.81, d (9.5)	4.80, d (9.7)	4.90, d (9.6)	4.84, d (9.8)
2′′′	2.57, overlapped 2.03, overlapped	2.42, dd (12.8, 5.4) 1.75, overlapped	2.42, overlapped 1.75, overlapped	2.45, dd (12.0, 4.8) 1.77, overlapped	2.55, dd (12.2, 5.3) 2.02, overlapped	2.46, overlapped 1.79, overlapped	2.45, overlapped 1.80, overlapped	2.58, overlapped 2.04, overlapped	2.48, br. d (12.5) 1.82, overlapped
3′′′	4.07, m	3.65, m	3.67, m	3.68, m	4.03, ddd (13.6, 8.6, 5.3)	3.64, overlapped	3.64, overlapped	4.04, ddd (13.4, 8.5, 5.2)	3.66, overlapped
4′′′	3.42, t (9.0)	3.74, t (9.0)	3.74, t (9.0)	3.76, t (8.9)	3.39, t (9.6)	3.63, overlapped	3.65, overlapped	3.40, t (9.0)	3.64, overlapped
5′′′	3.68, dq (9.0, 6.5)	3.68, dq (15.0, 6.0)	3.73, overlapped	3.75, m	3.64, overlapped	3.61, overlapped	3.58, overlapped	3.66, overlapped	3.66, overlapped
6′′′	1.67, d (6.1), 3H	1.73, d (6.5), 3H	1.75, d (6.0), 3H	1.78, d (5.9), 3H	1.60, d (6.0), 3H	1.66, d (5.8), 3H	1.66, d (6.0), 3H	1.61, d (6.3), 3H	1.69, d (4.3), 3H
-OMe		3.52, s, 3H	3.51, s, 3H	3.52, s, 3H		3.53, s, 3H	3.54, s, 3H		3.56, s, 3H
**Sugar moieties**	**6-deoxy-3-*O*-deMe-D-Allo**	**D-Glc**	**D-Glc**	**D-Glc**	**6-deoxy-3-*O*-deMe-D-Allo**	**6-deoxy-3-*O*-deMe-D-Allo**	**6-deoxy-3-*O*-deMe-D-Allo**	**6-deoxy-3-*O*-deMe-D-Allo**	**6-deoxy-3-*O*-deMe-D-Allo**
1′′′′	5.18, d (8.3)	5.15, d (7.8)	5.15, d (7.8)	5.16, d (7.8)	5.15, d (8.0)	5.31, d (8.2)	5.33, d (8.1)	5.15, d (8.0)	5.34, d (8.2)
2′′′′	3.98, dd (8.3, 3.0)	4.03, br. t (7.0)	4.03, br. t (8.0)	4.04, br. t (7.7)	3.96, overlapped	3.89, m	3.91, m	3.97, dd (8.1, 3.0)	3.93, overlapped
3′′′′	4.10, t (3.0)	4.24, overlapped	4.24, overlapped	4.25, overlapped	4.09, t (3.0)	4.10, overlapped	4.10, t (2.7)	4.09, t (3.0)	4.10, m
4′′′′	3.64, m	4.24, overlapped	4.24, overlapped	4.24, overlapped	3.63, overlapped	3.63, overlapped	3.65, overlapped	3.64, overlapped	3.65, overlapped
5′′′′	4.24, dq (10.0, 6.5)	3.96, m	3.95, m	3.96, m	4.23, dq (9.7, 6.3)	4.17, m	4.19, overlapped	4.23, m	4.18, overlapped
6′′′′	1.49, d (6.5), 3H	4.54, br. d (11.0) 4.38, dd (11.0, 6.0)	4.54, d (11.7) 4.37, dd (11.4, 5.4)	4.54, d (11.4) 4.38, dd (11.4, 5.5)	1.49, d (6.0), 3H	1.55, d (6.2), 3H	1.56, d (6.2), 3H	1.49, d (6.0), 3H	1.56, d (6.3), 3H
-OMe	3.84, s	3.52, s, 3H			3.84, s, 3H	3.84, s, 3H	3.85, s, 3H	3.84, s, 3H	3.85, s, 3H

**Table 3:** ^13^C NMR Data of Compounds **1**−**9** (150 MHz, *δ*_H_ in ppm, *J* in Hz, pyridine-*d*_5_).

No.	1	2	3	4	5	6	7	8	9
1	38.5, CH_2_	40.3, CH_2_	39.6, CH_2_	39.4, CH_2_	39.7, CH_2_	40.1, CH_2_	40.2, CH_2_	40.2, CH_2_	40.0, CH_2_
2	29.4, CH_2_	30.0, CH_2_	29.7, CH_2_	29.8, CH_2_	30.0, CH_2_	30.8, CH_2_	30.8, CH_2_	30.9, CH_2_	30.8, CH_2_
3	76.2, CH	77.7, CH	76.0, CH	76.2, CH	78.1, CH	78.1, CH_2_	78.1, CH_2_	77.8, CH	77.0, CH
4	34.1, CH_2_	39.7, CH_2_	35.0, CH_2_	35.0, CH_2_	40.8, CH_2_	40.0, CH_2_	40.1, CH_2_	39.1, CH_2_	35.9, CH_2_
5	43.7, CH	139.6, C	45.6, CH	45.6, CH	140.5, C	140.8, C	141.2, C	140.5, C	45.6, CH
6	30.6, CH_2_	118.8, CH	25.3, CH_2_	25.3, CH_2_	118.5, CH	122.6, CH	122.5, CH	122.9, CH	28.9, CH_2_
7	42.5, CH_2_	35.5, CH_2_	35.6, CH_2_	35.6, CH_2_	35.6, CH_2_	28.5, CH_2_	27.9, CH_2_	28.2, CH_2_	30.1, CH_2_
8	211.5, C	76.0, C	78.3, C	78.3, C	75.9, C	37.6, CH	38.2, CH	38.0, CH	40.4, CH
9	62.5, CH	49.2, CH	51.4, CH	51.3, CH	50.4, CH	49.9, CH	50.4, CH	48.5, CH	52.5, CH
10	42.7, C	39.2, C	38.4, C	38.4, C	39.5, C	39.6, C	40.0, C	39.7, C	38.3, C
11	76.5, CH	71.5, CH	72.0, CH	71.2, CH	68.5, CH	72.0, CH	71.0, CH	75.5, CH	72.3, CH
12	81.0, CH	78.3, CH	79.6, CH	79.7, CH	81.1, CH	78.5, CH	76.2, CH	72.4, CH	79.4, CH
13	55.8, C	55.5, C	55.7, C	55.6, C	55.8, C	55.9, C	55.3, C	57.2, C	56.0, C
14	115.7, C	85.5, C	85.5, C	85.5, C	85.6, C	85.1, C	85.6, C	85.5, C	84.9 C
15	36.8, CH_2_	36.6, CH_2_	36.2, CH_2_	36.1, CH_2_	36.6, CH_2_	35.3, CH_2_	31.9, CH_2_	32.4, CH_2_	34.8, CH_2_
16	23.3, CH_2_	24.2, CH_2_	24.7, CH_2_	24.7, CH_2_	24.3, CH_2_	24.6, CH_2_	21.2, CH_2_	21.5, CH_2_	24.9, CH_2_
17	58.1, CH	59.2, CH	59.4, CH	59.4, CH	59.6, CH	58.9, CH	61.4, CH	61.9, CH	59.1, CH
18	12.1, CH_3_	13.5, CH_3_	14.1, CH_3_	14.0, CH_3_	13.8, CH_3_	11.2, CH_3_	15.8, CH_3_	4.9, CH_3_	11.4, CH_3_
19	12.5, CH_3_	18.1, CH_3_	13.4, CH_3_	13.3, CH_3_	17.4, CH_3_	19.2, CH_3_	19.2, CH_3_	19.8, CH_3_	12.7, CH_3_
20	207.7, C	213.1, C	213.6, C	213.7, C	213.9, C	217.0, C	208.9 C	209.9, C	216.7, C
21	31.1, CH_3_	31.2, CH_3_	31.7, CH_3_	31.6, CH_3_	31.4, CH_3_	32.8, CH_3_	31.7, CH_3_	32.0, CH_3_	32.8, CH_3_
	**11-Bz**	**11-Tig**	**11-Bz**	**11-Tig**				**11-Ac**	
1′	165.9, C	166.8, C	165.9, C	166.9, C				170.9, C	
2′	130.2, C	129.1, C	130.5, C	128.9, C				22.4, CH_3_	
3′(-7′)	129.9, CH	138.3, CH	129.8, CH	138.5, CH					
4′(-6′)	128.9, CH	14.2, CH_3_	128.4, CH	14.0, CH_3_					
5′	133.5, CH	12.0, CH_3_	133.0, CH	11.6, CH_3_					
		**12-Tig**	**12-Bz**	**12-Bz**	**12-Tig**		**12-Ac**		
1′′		167.9, C	166.8, C	166.8, C	168.3, C		170.4, C		
2′′		128.4, C	129.9, C	130.3, C	129.2, C		21.3, CH_3_		
3′′(-7′′)		138.5, CH	129.8, CH	130.0, CH	137.2, CH				
4′′(-6′′)		14.2, CH_3_	128.6, CH	128.8, CH	14.0, CH_3_				
5′′		12.0, CH_3_	133.3, CH	133.5, CH	12.2, CH_3_				
	**3-*O*-deMe-D-Ole**	**D-Ole**	**D-Ole**	**D-Ole**	**3-*O*-deMe-D-Ole**	**D-Ole**	**D-Ole**	**3-*O*-deMe-D-Ole**	**D-Ole**
1′′′	97.6, CH	97.9, CH	97.3, CH	97.4, CH	97.8, CH	98.1, CH	98.2, CH	98.5, CH	97.8, CH
2′′′	40.0, CH_2_	37.5, CH_2_	37.5, CH_2_	37.5, CH2	40.1, CH_2_	38.1, CH_2_	38.2, CH_2_	40.5, CH_2_	38.2, CH_2_
3′′′	70.1, CH	79.5, CH	79.5, CH	79.5, CH	70.1, CH	79.9, CH	79.9, CH	70.6, CH	80.0, CH
4′′′	88.6, CH	83.4, CH	83.4, CH	83.4, CH	88.6, CH	83.6, CH	83.6, CH	89.0, CH	83.7, CH
5′′′	71.2, CH	71.9, CH	71.9, CH	71.9, CH	71.1, CH	72.2, CH	72.3, CH	71.6, CH	72.4, CH
6′′′	18.3, CH_3_	18.9, CH_3_	18.9, CH_3_	18.9, CH	18.2, CH_3_	19.4, CH_3_	19.4, CH_3_	18.7, CH_3_	19.5,CH_3_
-OMe		57.0, CH_3_	56.9, CH_3_	57.0, CH_3_		57.5, CH_3_	57.5, CH_3_		57.5, CH_3_
	**6-deoxy-3-*O*-deMe-D-Allo**	**D-Glc**	**D-Glc**	**D-Glc**	**6-deoxy-3-*O*-deMe-D-Allo**	**6-deoxy-3-*O*-deMe-D-Allo**	**6-deoxy-3-*O*-deMe-D-Allo**	**6-deoxy-3-*O*-deMe-D-Allo**	**6-deoxy-3-*O*-deMe-D-Allo**
1′′′′	103.2, CH	104.4, CH	104.4, CH	104.4, CH	103.2, CH	102.4, CH	102.5, CH	103.7, CH	102.4, CH
2′′′′	72.5, CH	75.6, CH	75.6, CH	75.6, CH	72.5, CH	73.5, CH	73.6, CH	73.0, CH	73.6, CH
3′′′′	83.8, CH	78.6, CH	78.6, CH	78.6, CH	83.8, CH	84.3, CH	84.4, CH	84.3, CH	84.4, CH
4′′′′	74.1, CH	71.8, CH	71.8, CH	71.8, CH	74.0, CH	74.9, CH	74.9, CH	74.5, CH	74.9, CH
5′′′′	71.1, CH	78.0, CH	78.0, CH	78.0, CH	71.1, CH	71.2, CH	71.3, CH	71.5, CH	71.3, CH
6′′′′	18.0, CH_3_	62.9, CH_2_	62.9, CH_2_	62.9, CH_2_	18.0, CH_3_	18.9, CH_3_	19.0, CH_3_	18.5, CH_3_	19.0, CH_3_
-OMe	62.1, CH_3_				62.0, CH_3_	62.4, CH_3_	62.4, CH_3_	62.5, CH_3_	62.4, CH_3_

**Table 4:** Inhibitory activities of compounds **1**, **8**, and **9** on NO production by lipopolysaccharide-activated macrophage (RAW264.7).

Compounds	IC_50_ (μM)
L-NMMA	39.30 ± 1.23
**1**	37.46 ± 1.91
**8**	38.80 ± 0.76
**9**	42.78 ± 1.43

L-NMMA (N^G^-Monomethyl-L-Arginine, monoacetate salt) was used as positive control.

## Data Availability

All the data in this research were presented in the manuscript and Supplementary Material.

## Supplementary Materials

The following supporting information can be downloaded at https://www.mdpi.com/article/10.3390/molecules28062705/s1; Figure S1–S6 NMR spectra of Marsdeoside A (**1**), Figure S7 HRESIMS spectrum of Marsdeoside A (**1**), Figure S8–S13 NMR spectra of Marsdeoside B (**2**), Figure S14 HRESIMS spectrum of Marsdeoside B (**2**), Figure S15–S20 NMR spectra of Marsdeoside C (**3**), Figure S21 HRESIMS spectrum of Marsdeoside C (**4**), Figure S22–27 NMR spectra of Marsdeoside D (**4**), Figure S28 HRESIMS spectrum of Marsdeoside D (**4**), Figure S29–34 NMR spectra of Marsdeoside E (**5**), Figure S35 HRESIMS spectrum of Marsdeoside E (**5**), Figure S36–41 NMR spectra of Marsdeoside F (**6**), Figure S42 HRESIMS spectrum of Marsdeoside F (**6**), Figure S43–48 NMR spectra of Marsdeoside G (**7**), Figure S49 HRESIMS spectrum of Marsdeoside G (**7**), Figure S50–55 NMR spectra of Marsdeoside H (**8**), Figure S56 HRESIMS spectrum of Marsdeoside H (**8**), Figure S57–62 NMR spectra of Marsdeoside H (**9**), Figure S63 HRESIMS spectrum of Marsdeoside H (**9**).

## Sample Availability

Samples of the compounds are available from the authors upon reasonable request.
