# Supplementary material for: Nine New Pregnane Glycosides from the Cultivated Medicinal Plant Marsdenia tenacissima
Source: Molecules. 2023 Mar 16;28(6):2705. doi: 10.3390/molecules28062705 (PMC10058037; doi:10.3390/molecules28062705)
Supplement: Supplementary file 1 [file molecules-28-02705-s001.zip › molecules-2263044-supplementary.pdf]

Supplementary material

Nine New Pregnane Glycosides from the Cultivated Medicinal Plant *Marsdenia tenacissima*

Qian-Qian Meng [1,2], Shun-Yao Tong [2], Xing-Rong Peng [3], Yu-Qing Zhao [1], Zheng-Hui Li [2], He-Ping Chen [2,*] and Ji-Kai Liu [1,2,*]

[1] School of Pharmacy, Anhui University of Chinese Medicine, Hefei 230012, China
[2] School of Pharmaceutical Sciences, South-Central Minzu University, Wuhan 430074, China
[3] State Key Laboratory of Phytochemistry and Plant Resources in West China, Kunming Institute of Botany, Chinese Academy of Sciences, Kunming 650201, China
* Correspondence: chenhp@mail.scuec.edu.cn (H.-P.C.); liujikai@mail.scuec.edu.cn (J.-K.L.); Tel.: +86-27-67841275 (J.-K.L.); Fax: +86-27-67841275 (J.-K.L.)

Contents of Supporting Information

- **Detailed Experimental Procedure**
- **Figure S1-S6 NMR spectra of Marsdeoside A (1)**
- **Figure S7 HRESIMS spectrum of Marsdeoside A (1)**
- **Figure S8-S13 NMR spectra of Marsdeoside B (2)**
- **Figure S14 HRESIMS spectrum of Marsdeoside B (2)**
- **Figure S15-S20 NMR spectra of Marsdeoside C (3)**
- **Figure S21 HRESIMS spectrum of Marsdeoside C (4)**
- **Figure S22-27 NMR spectra of Marsdeoside D (4)**
- **Figure S28 HRESIMS spectrum of Marsdeoside D (4)**
- **Figure S29-34 NMR spectra of Marsdeoside E (5)**
- **Figure S35 HRESIMS spectrum of Marsdeoside E (5)**
- **Figure S36-41 NMR spectra of Marsdeoside F (6)**
- **Figure S42 HRESIMS spectrum of Marsdeoside F (6)**
- **Figure S43-48NMR spectra of Marsdeoside G (7)**
- **Figure S49 HRESIMS spectrum of Marsdeoside G (7)**
- **Figure S50-55 NMR spectra of Marsdeoside H (8)**
- **Figure S56 HRESIMS spectrum of Marsdeoside H (8)**
- **Figure S57-62 NMR spectra of Marsdeoside H (9)**
- **Figure S63 HRESIMS spectrum of Marsdeoside H (9)**

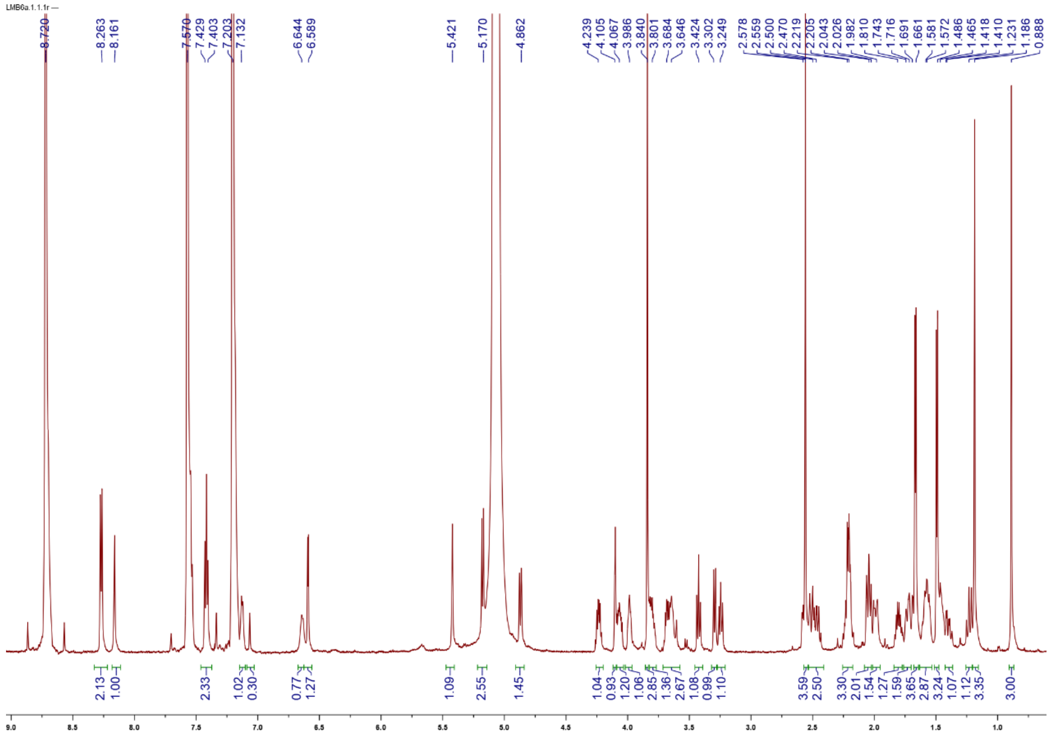

Figure S1. ^{1}H NMR (600 MHz, Pyridine-d$_5$) spectrum of Marsdeoside A (**1**).

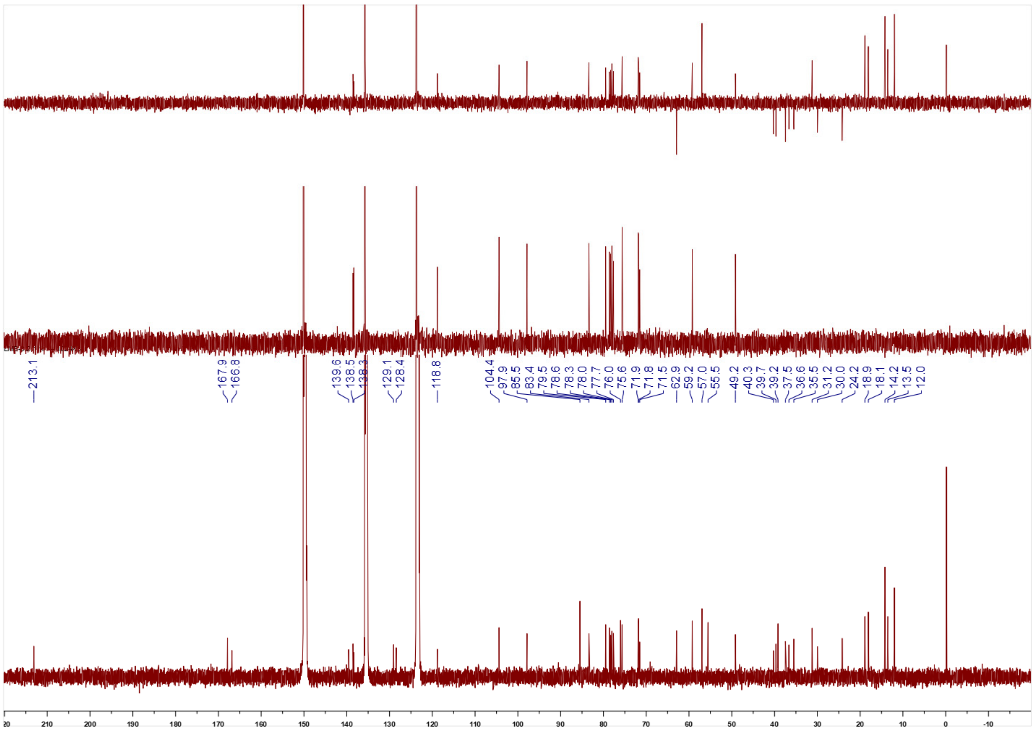

Figure S2. ^{13}C NMR (150 MHz, Pyridine-d_5) spectrum of Marsdeoside A (**1**).

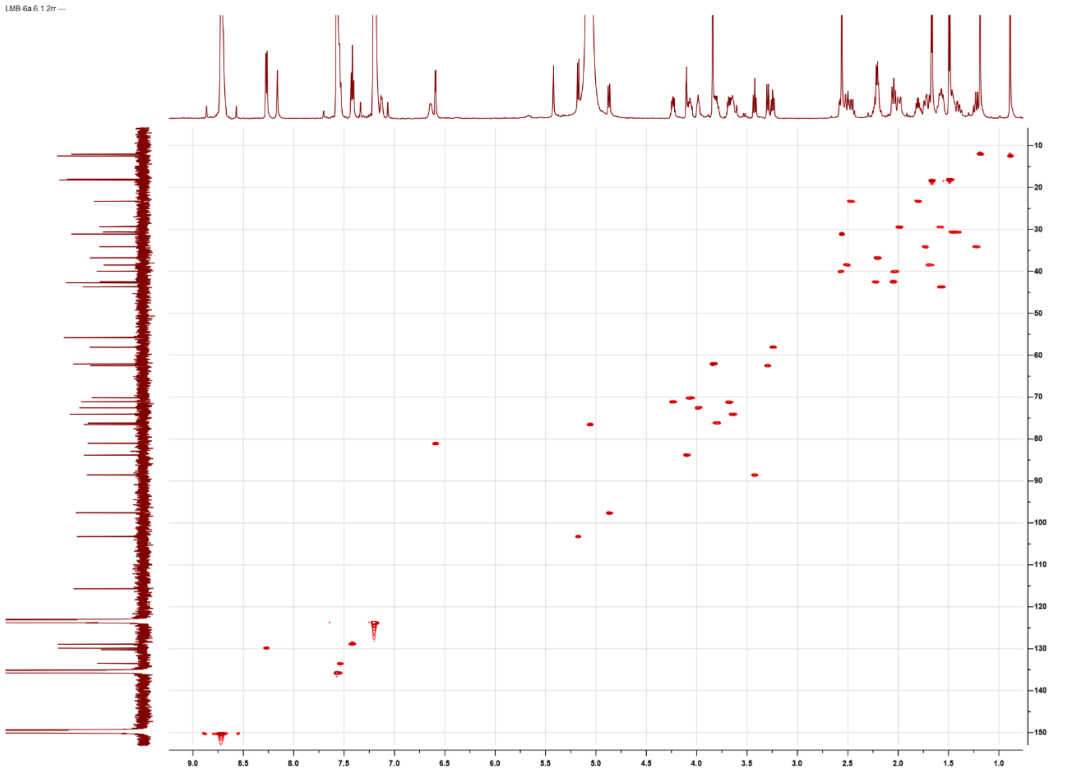

Figure S3. HSQC (600/150 MHz, Pyridine-d_5) spectrum of Marsdeoside A (**1**).

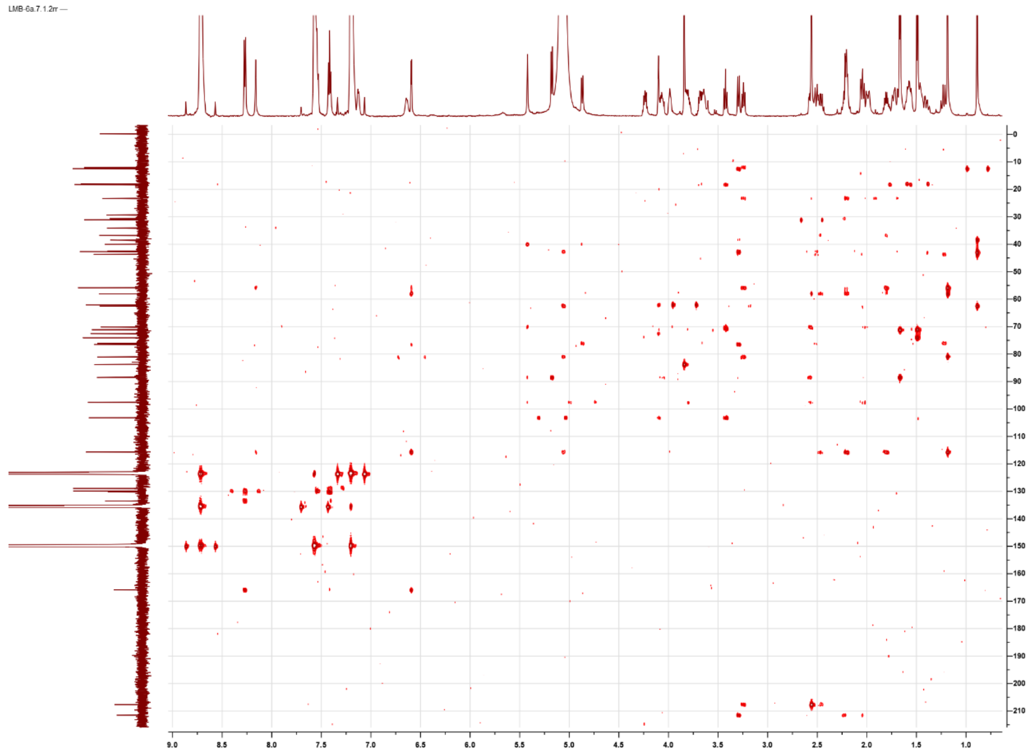

Figure S4. HMBC (600/150 MHz, Pyridine-d5) spectrum of Marsdeoside A (**1**).

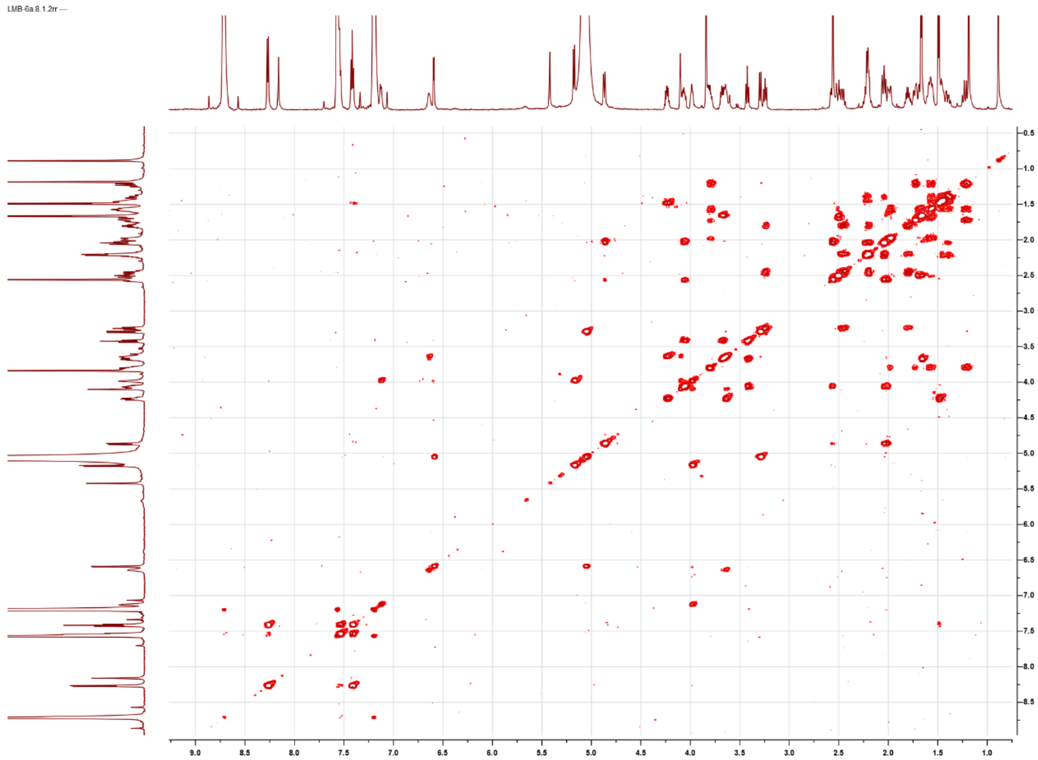

Figure S5. ^{1}H-^{1}H COSY (600/150 MHz, Pyridine-d5) spectrum of Marsdeoside A (**1**).

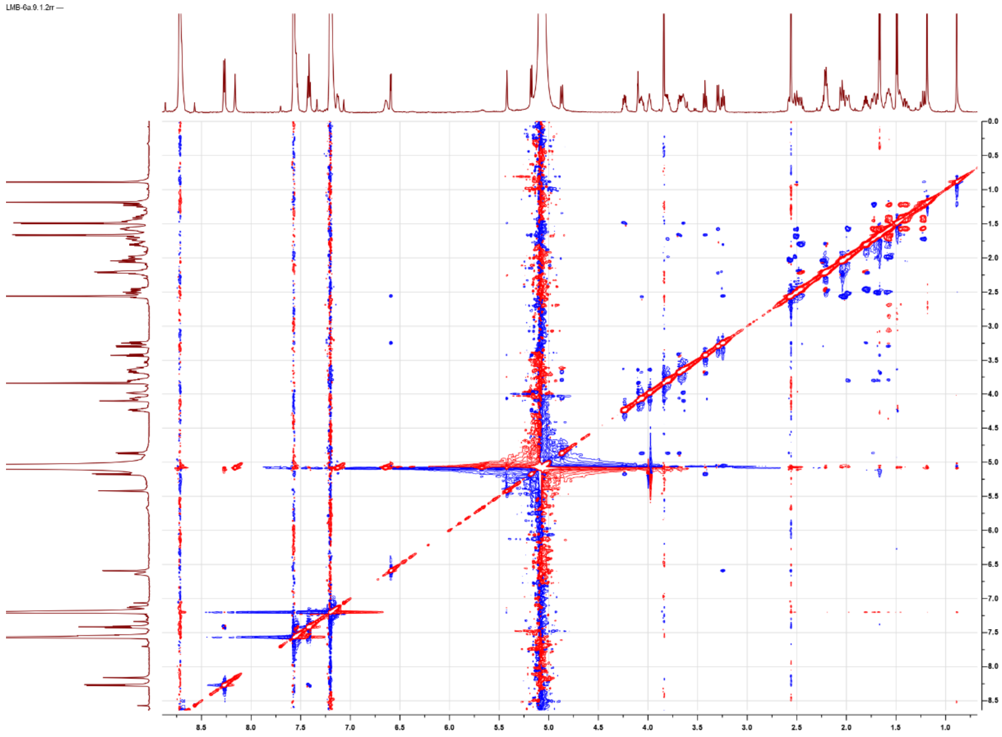

Figure S6. NOESY (600/150 MHz, Pyridine-d_5) spectrum of Marsdeoside A (**1**).

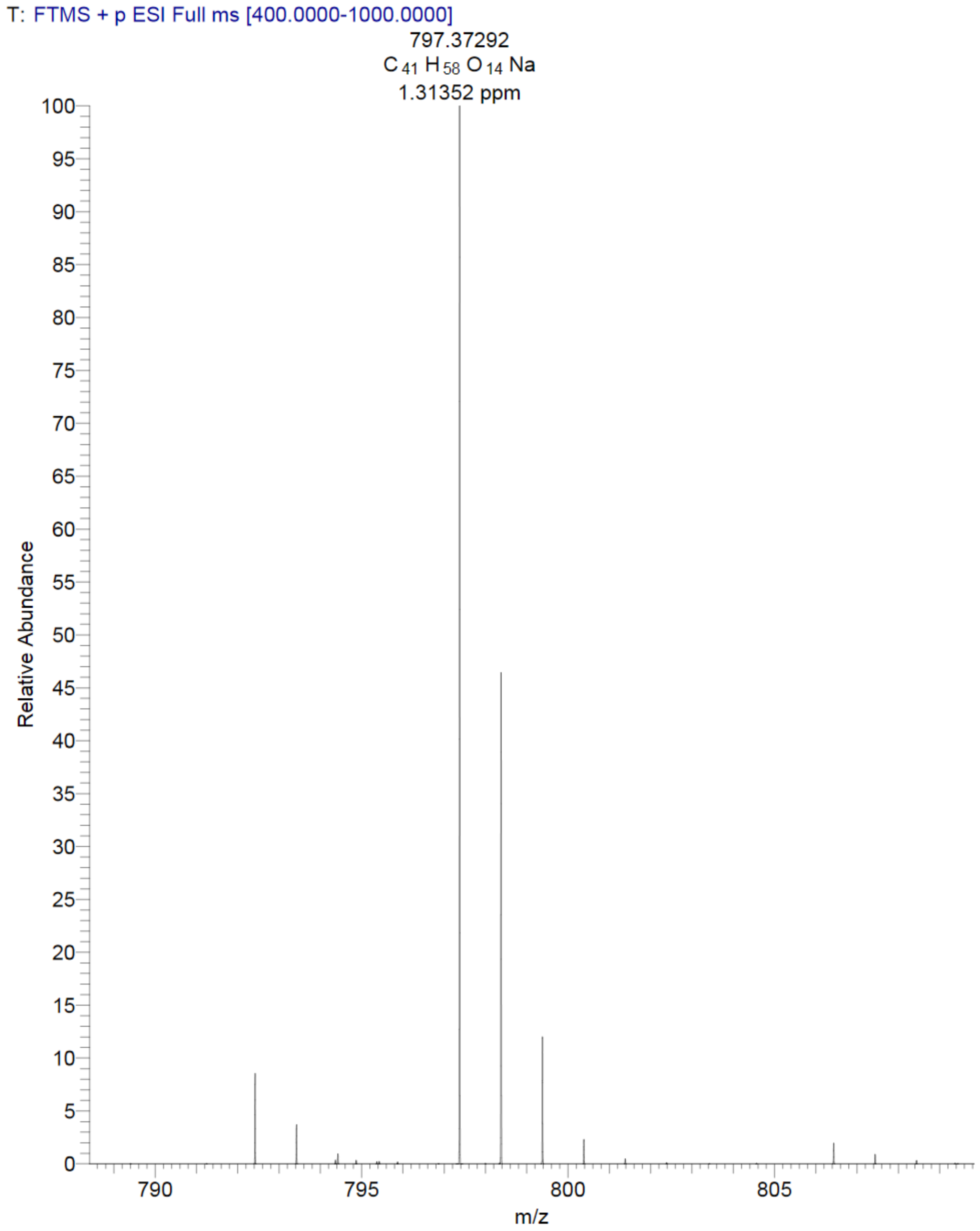

Figure S7. HRESIMS spectrum of Marsdeoside A (**1**).

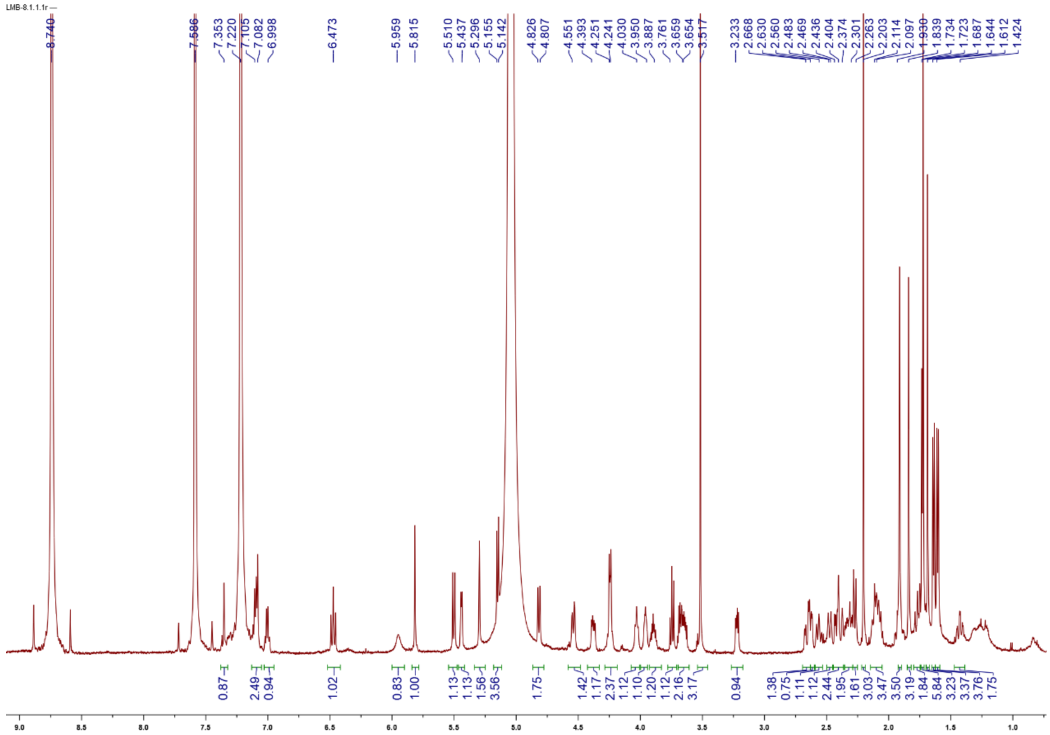

Figure S8. ^{1}H NMR (600 MHz, Pyridine-d_5) spectrum of Marsdeoside B (**2**).

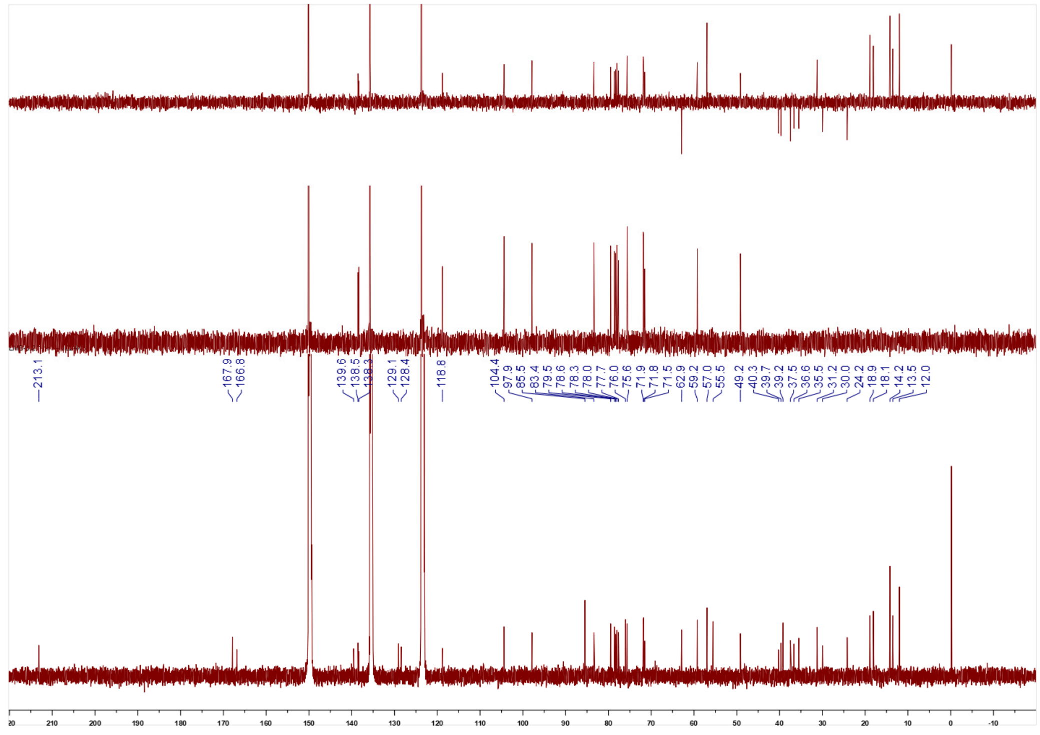

Figure S9. ^{13}C NMR (150 MHz, Pyridine-d_5) spectrum of Marsdeoside B (**2**).

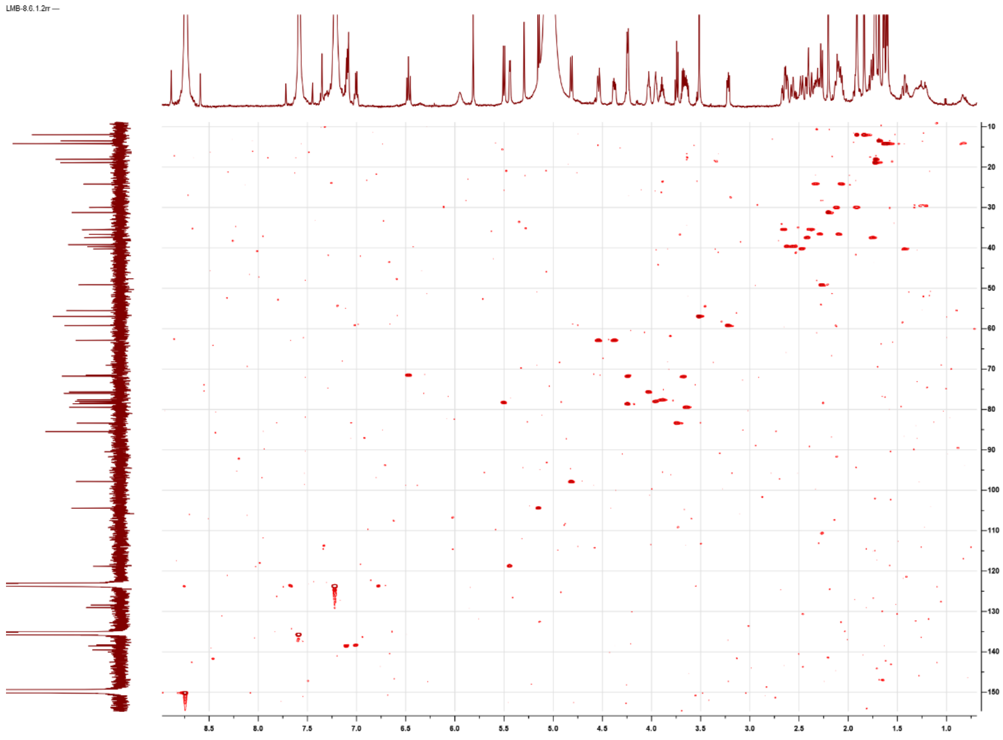

Figure S10. HSQC (600/150 MHz, Pyridine-d5) spectrum of Marsdeoside B (**2**).

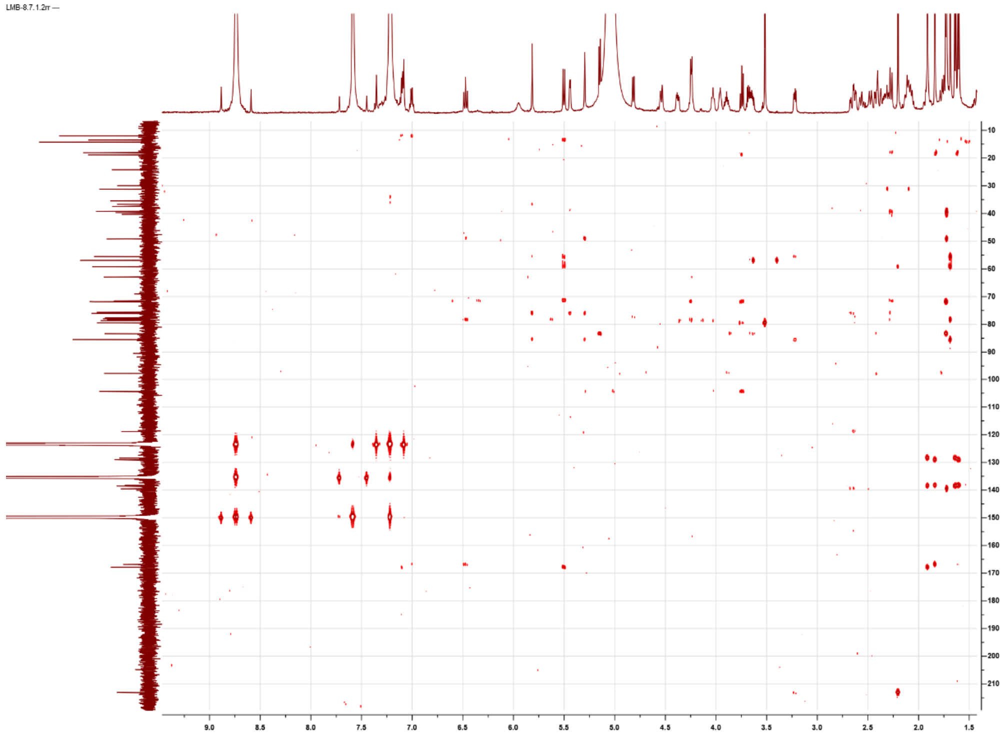

Figure S11. HMBC (600/150 MHz, Pyridine-d5) spectrum of Marsdeoside B (**2**).

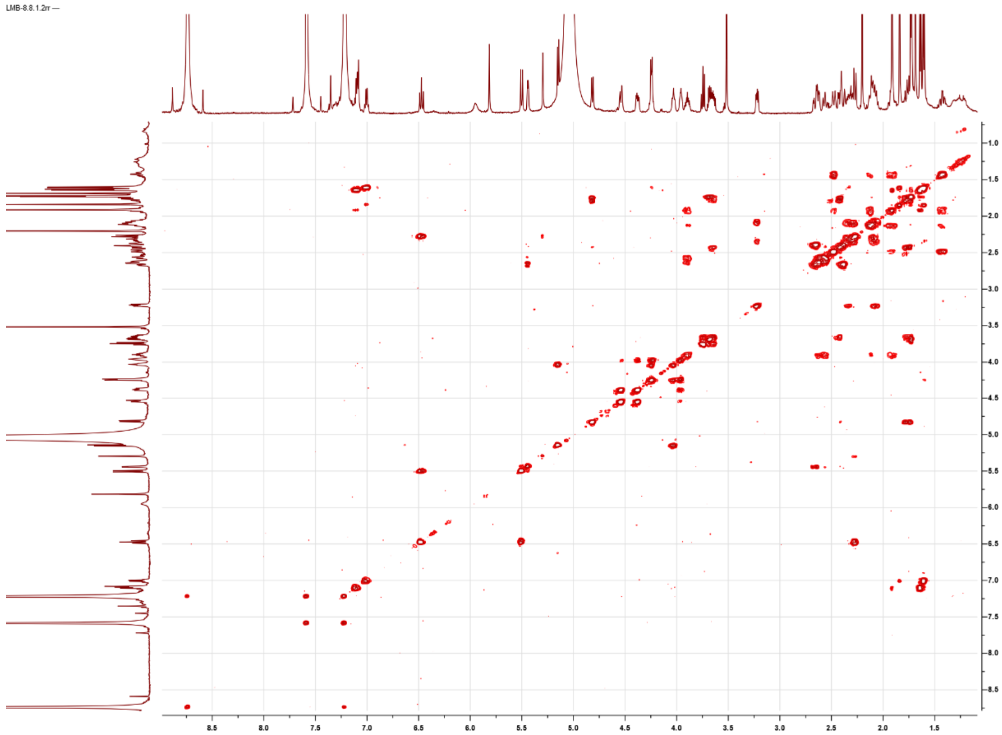

Figure S12. ^1H-^1H COSY (600/150 MHz, Pyridine-d_5) spectrum of Marsdeoside B (**2**).

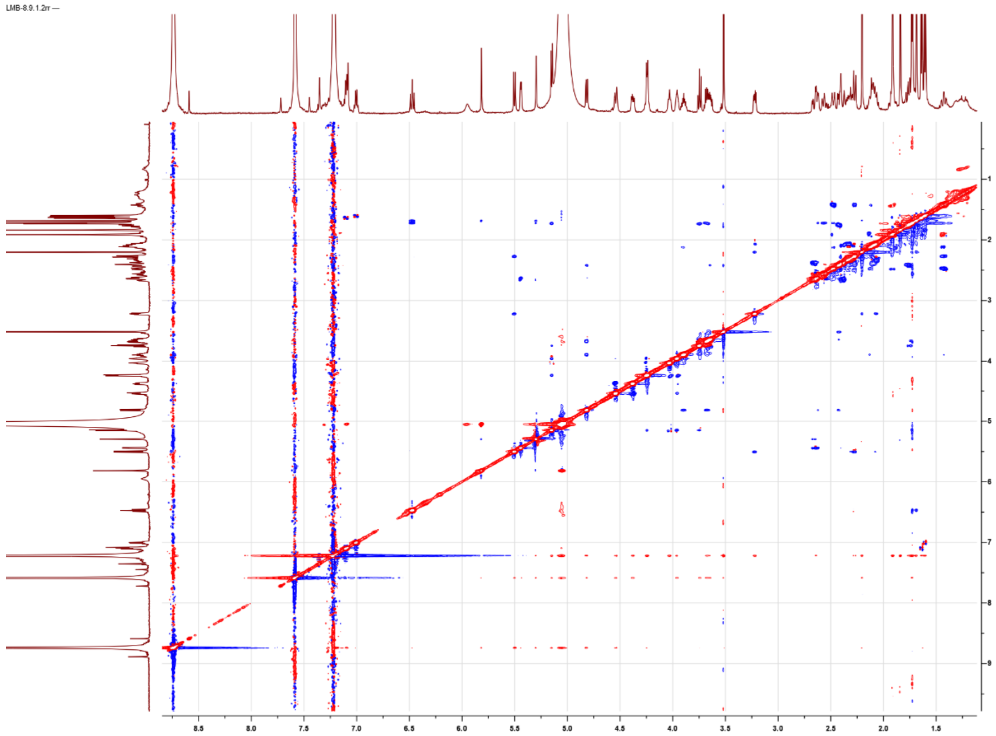

Figure S13. NOESY (600/150 MHz, Pyridine-d_5) spectrum of Marsdeoside B (**2**).

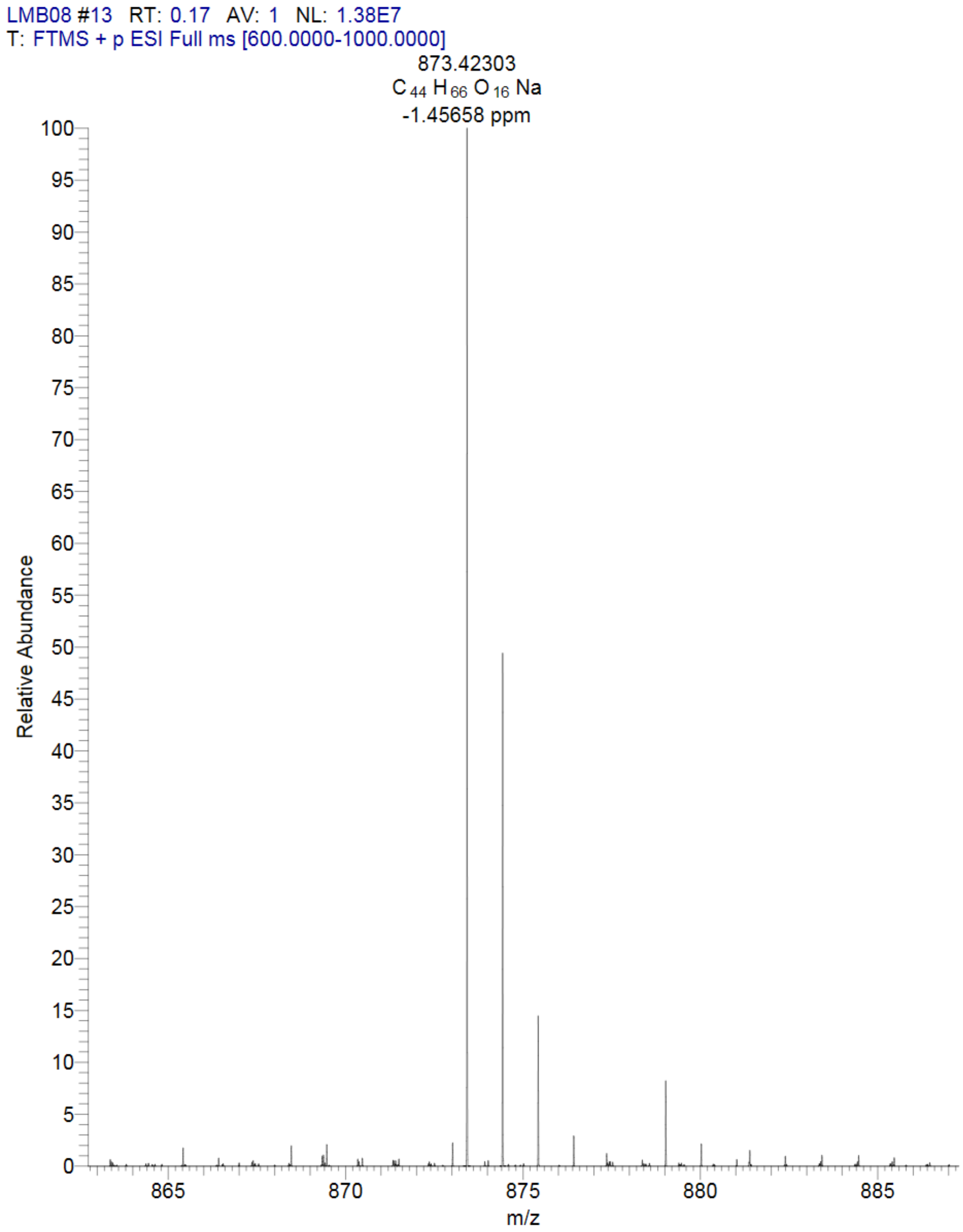

Figure S14. HRESIMS spectrum of Marsdeoside B (**2**).

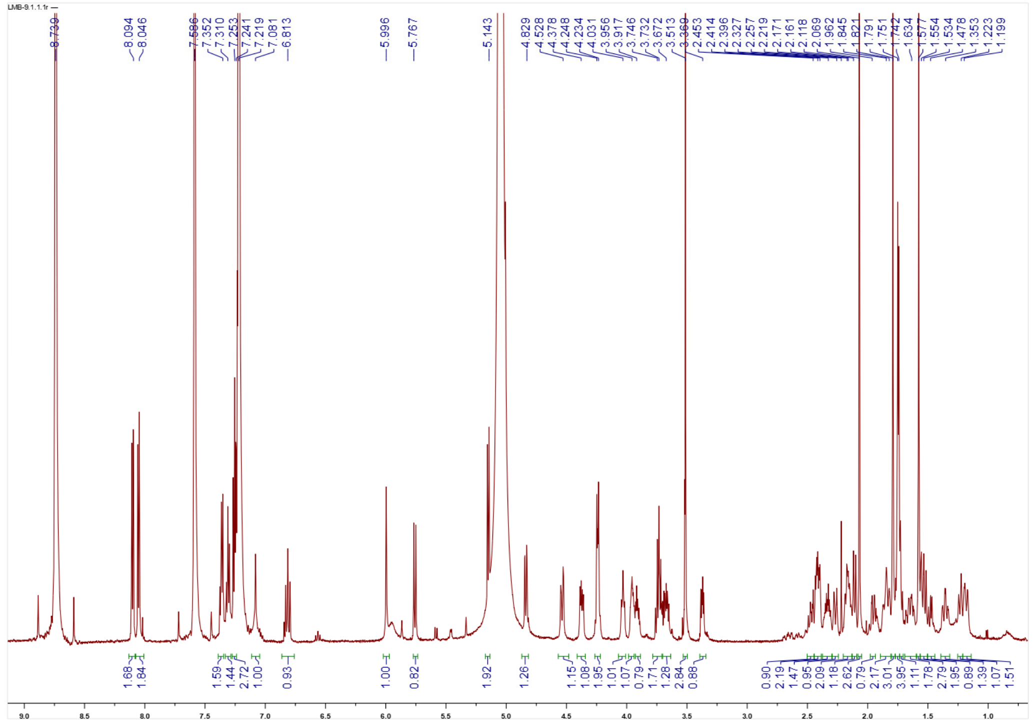

Figure S15. ^{1}H NMR (600 MHz, Pyridine-d_5) spectrum of Marsdeoside C (**3**).

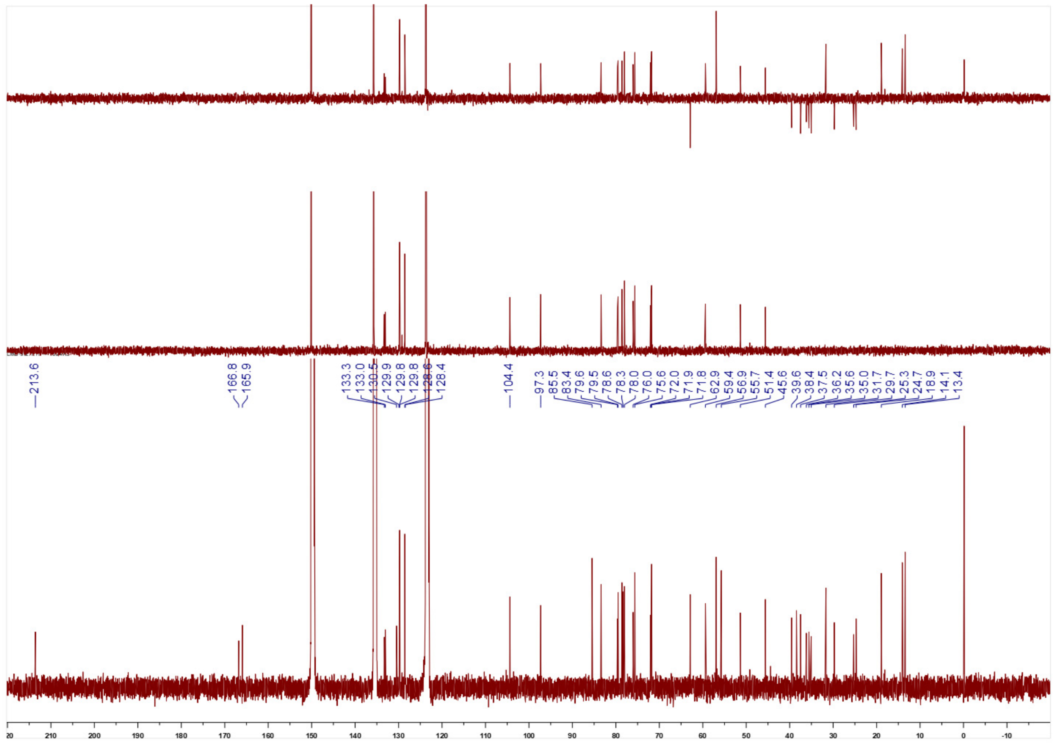

Figure S16. ^{13}C NMR (150 MHz, Pyridine-d_5) spectrum of Marsdeoside C (**3**).

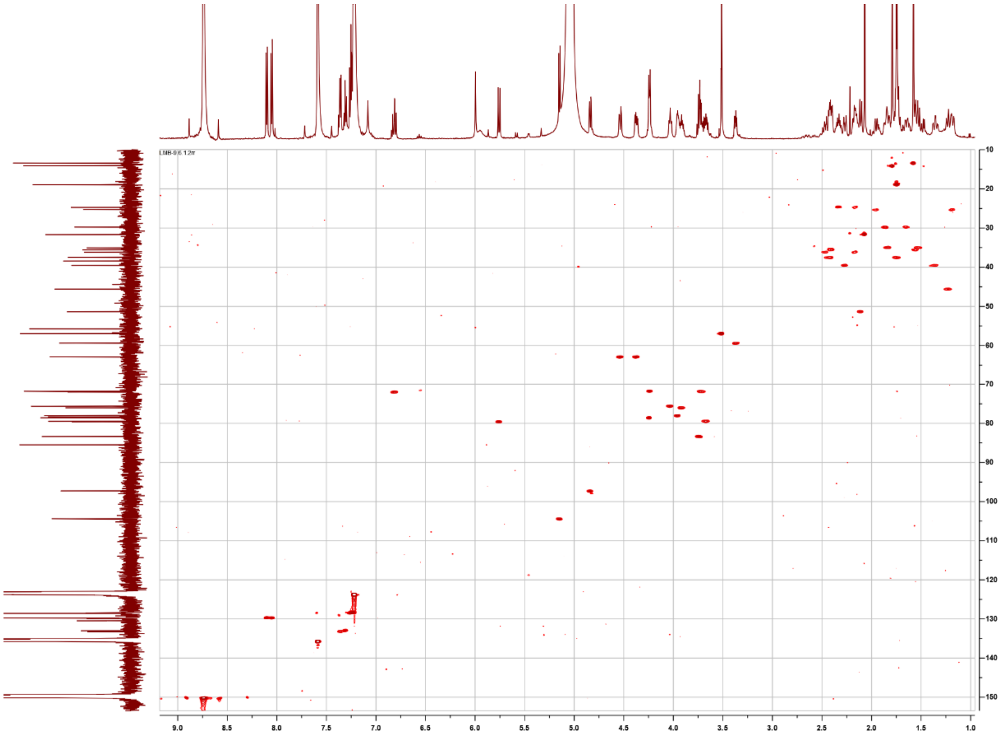

Figure S17. HSQC (600/150 MHz, Pyridine-d5) spectrum of Marsdeoside C (**3**).

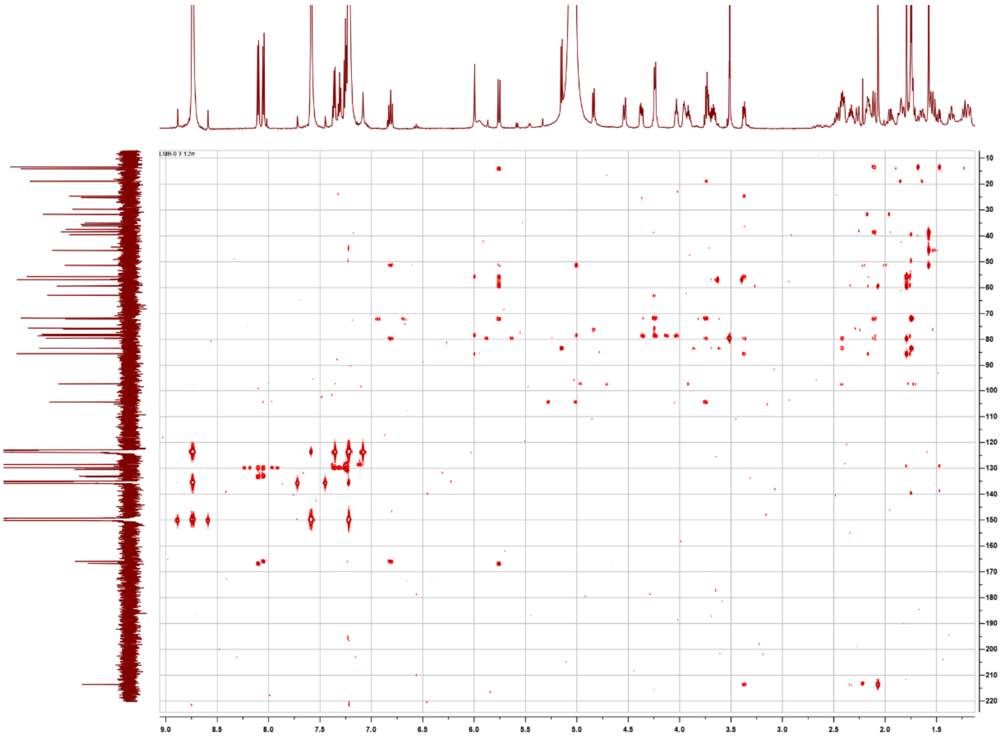

Figure S18. HMBC (600/150 MHz, Pyridine-d5) spectrum of Marsdeoside C (**3**).

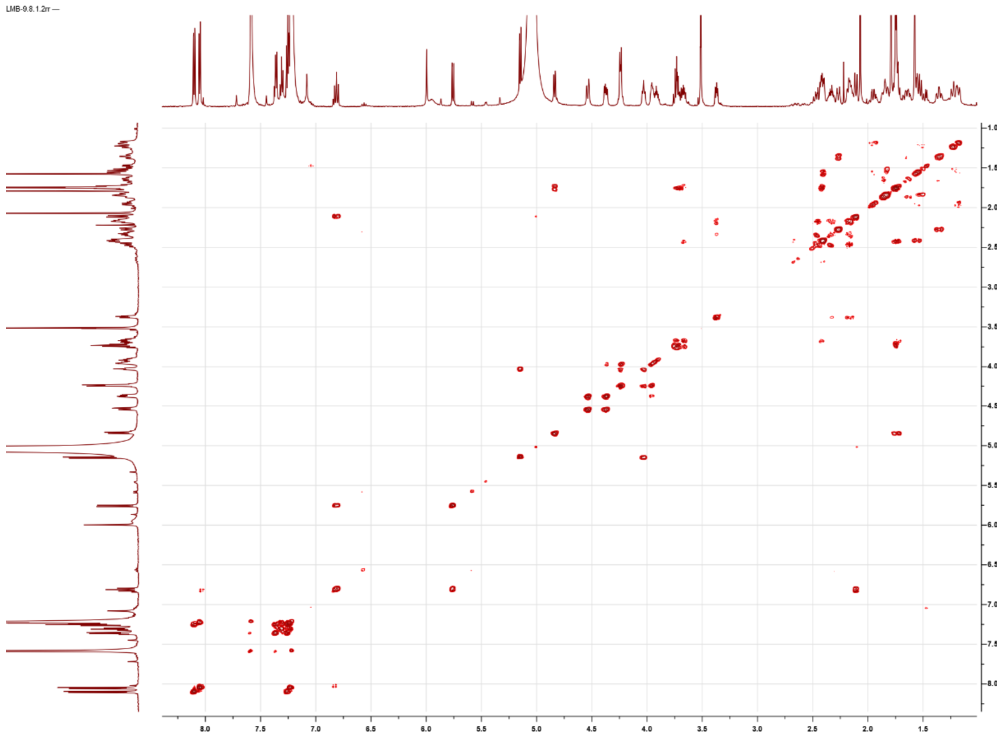

Figure S19. ^1H-^1H COSY (600/150 MHz, Pyridine-d_5) spectrum of Marsdeoside C (**3**).

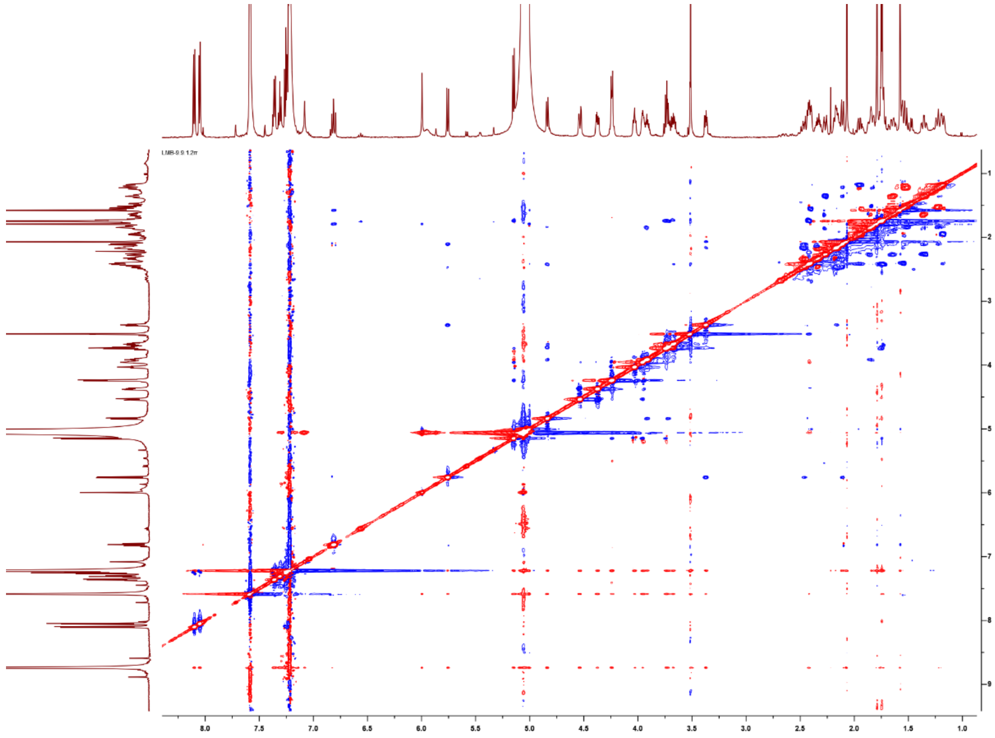

Figure S20. NOESY (600/150 MHz, Pyridine-d_5) spectrum of Marsdeoside C (**3**).

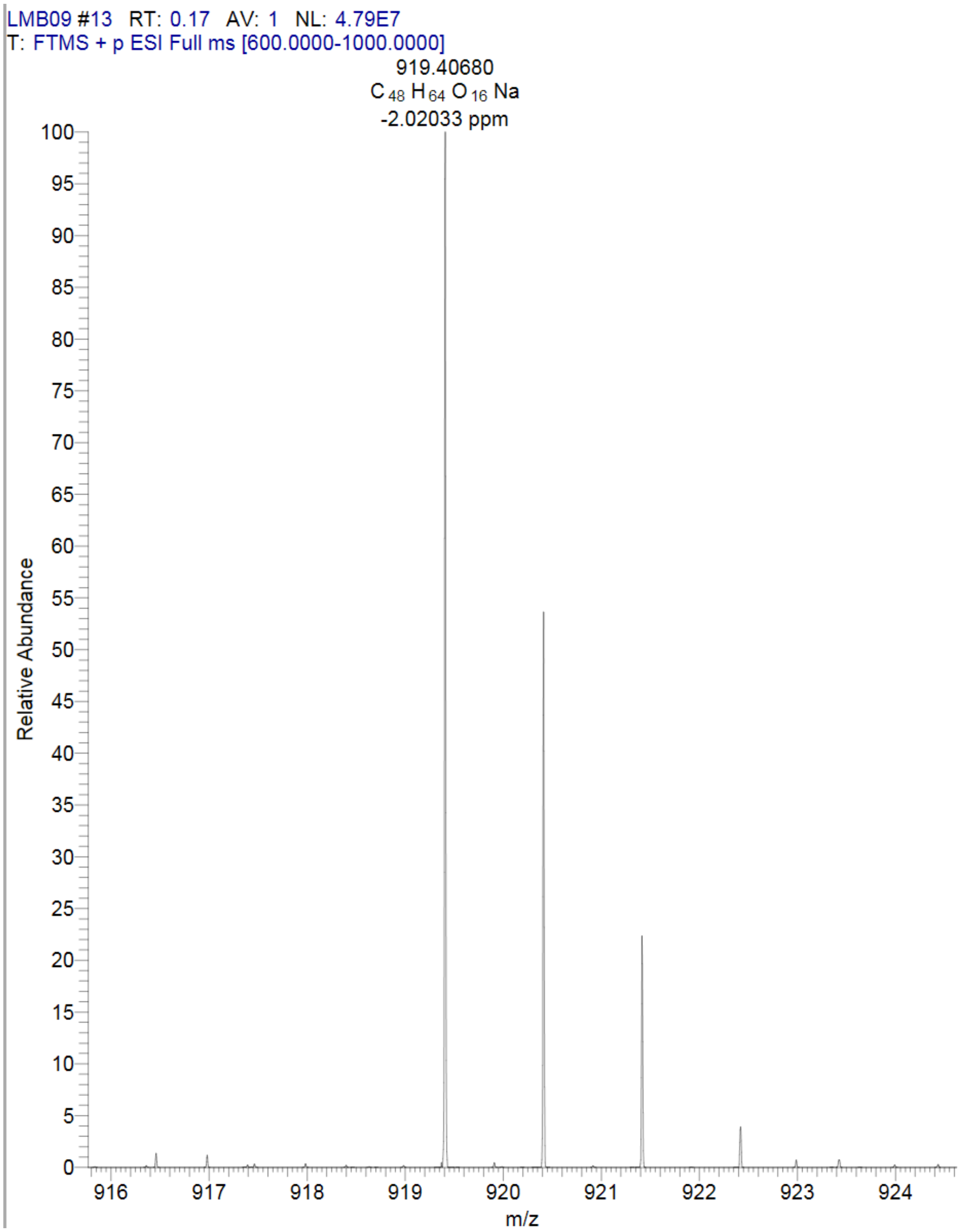

Figure S21. HRESIMS spectrum of Marsdeoside C (**3**).

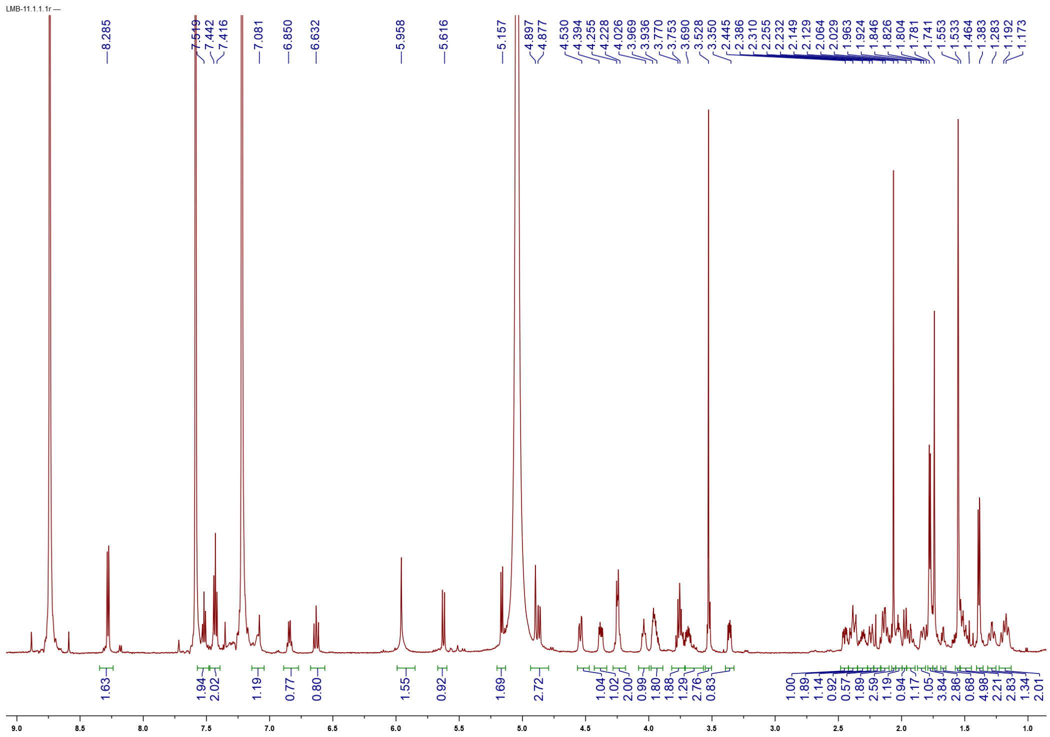

Figure S22. ^{1}H NMR (600 MHz, Pyridine-d_5) spectrum of Marsdeoside D (**4**).

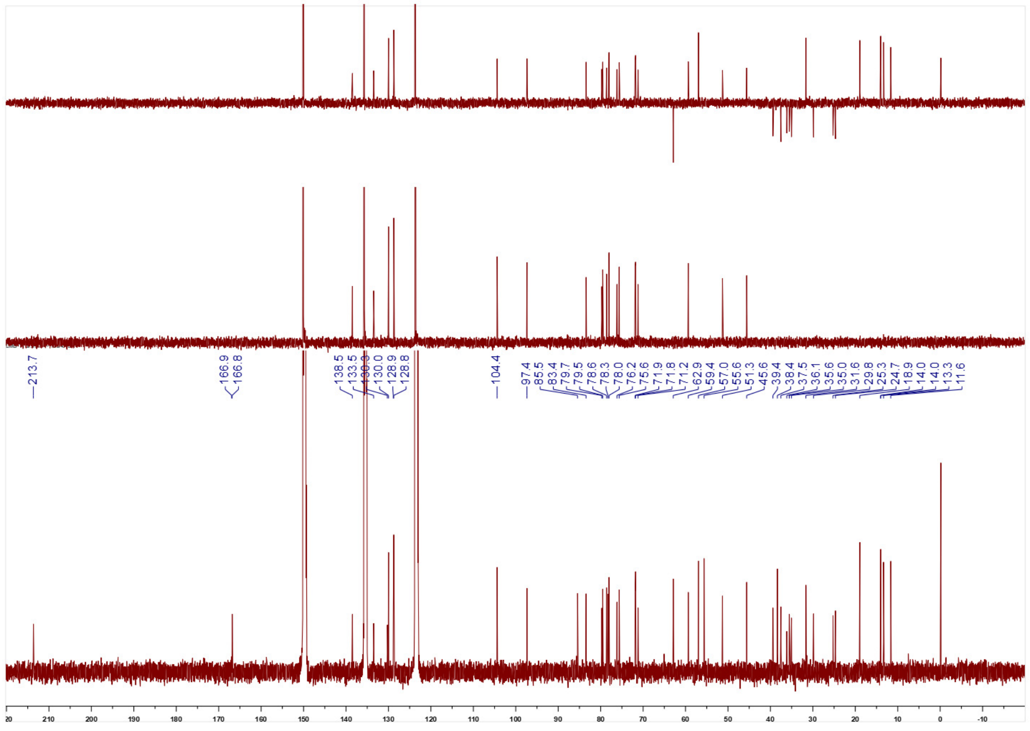

Figure S23. ^{13}H NMR (150 MHz, Pyridine-d_5) spectrum of Marsdeoside D (**4**).

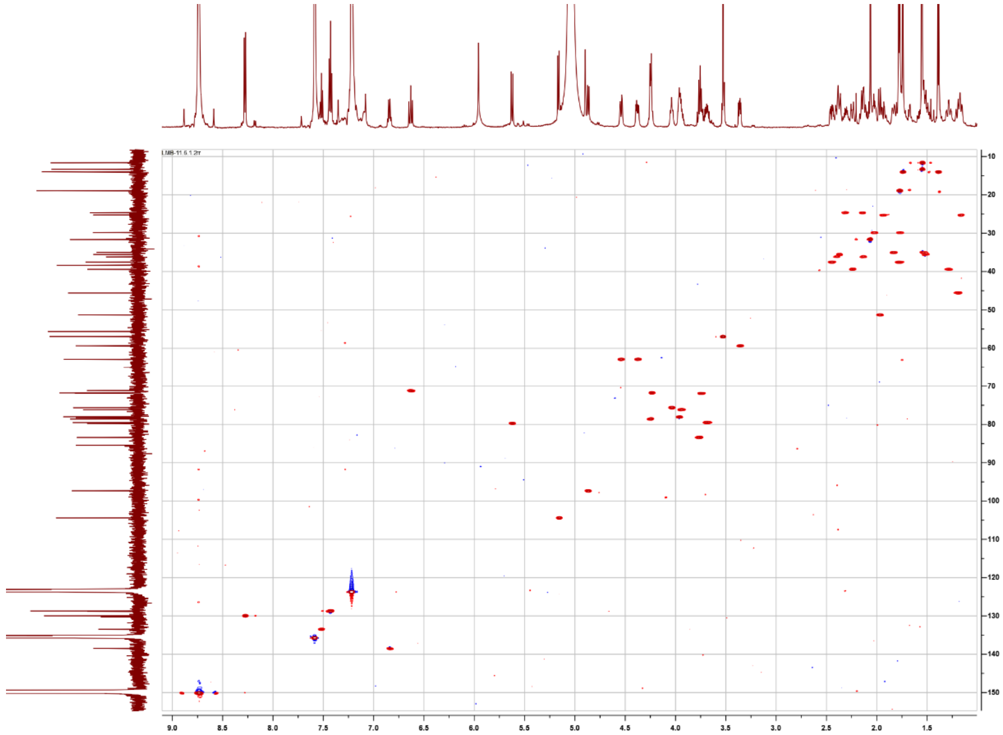

Figure S24. HSQC (600/150 MHz, Pyridine-d_5) spectrum of Marsdeoside D (**4**).

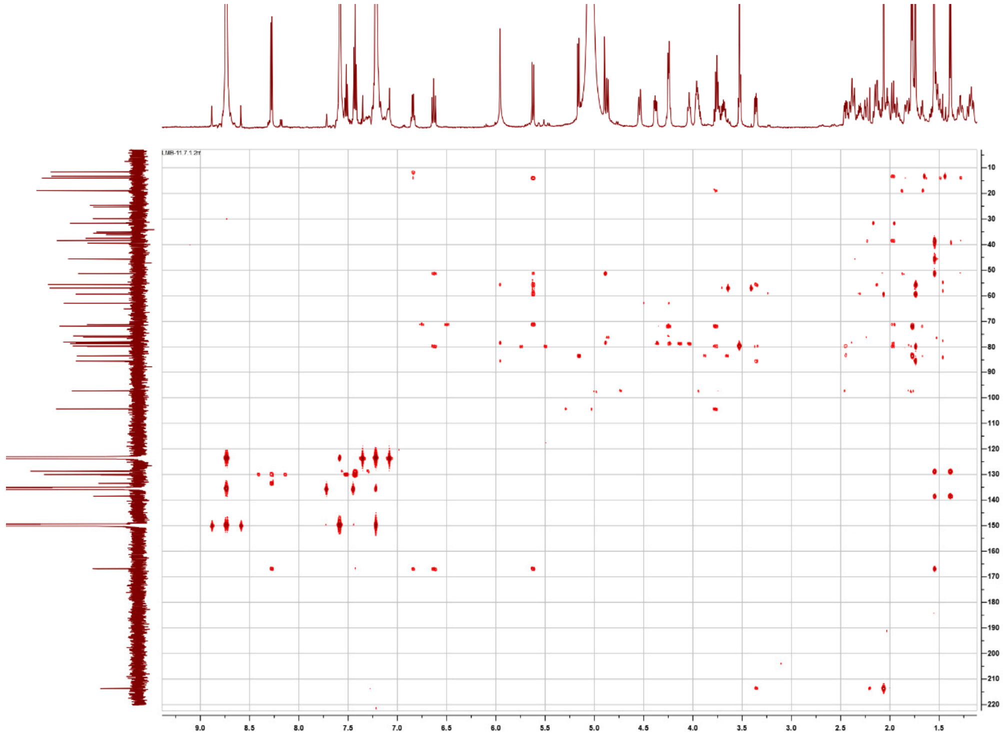

Figure S25. HMBC (600/150 MHz, Pyridine-d_5) spectrum of Marsdeoside D (**4**).

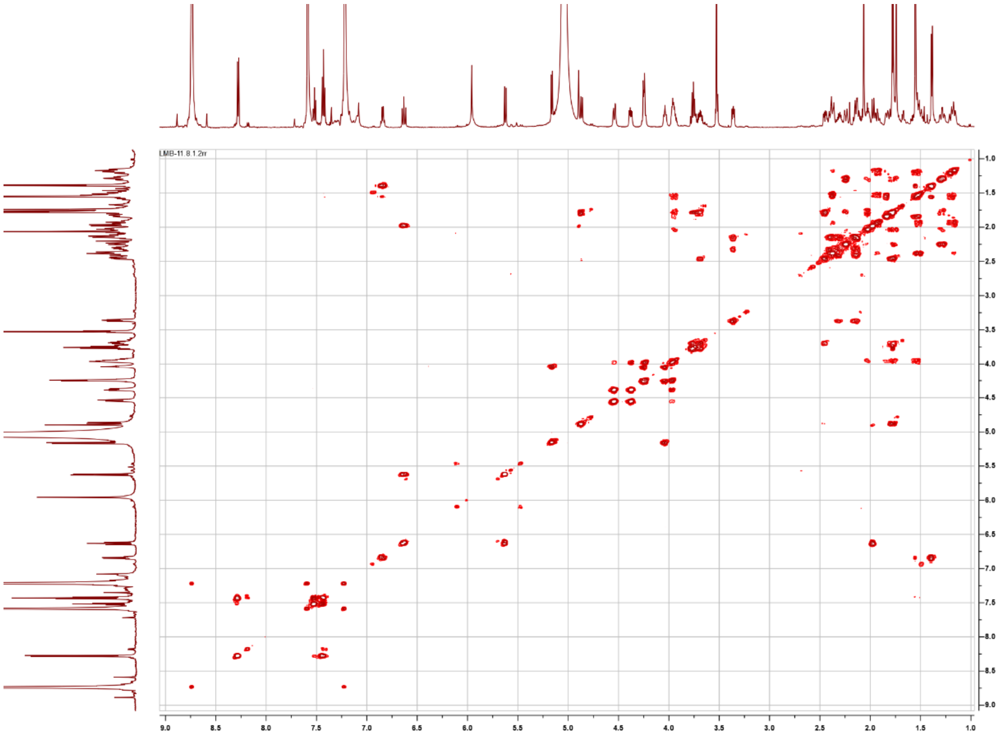

Figure S26. ^{1}H-^{1}H COSY (600/150 MHz, Pyridine-d_5) spectrum of Marsdeoside D (**4**).

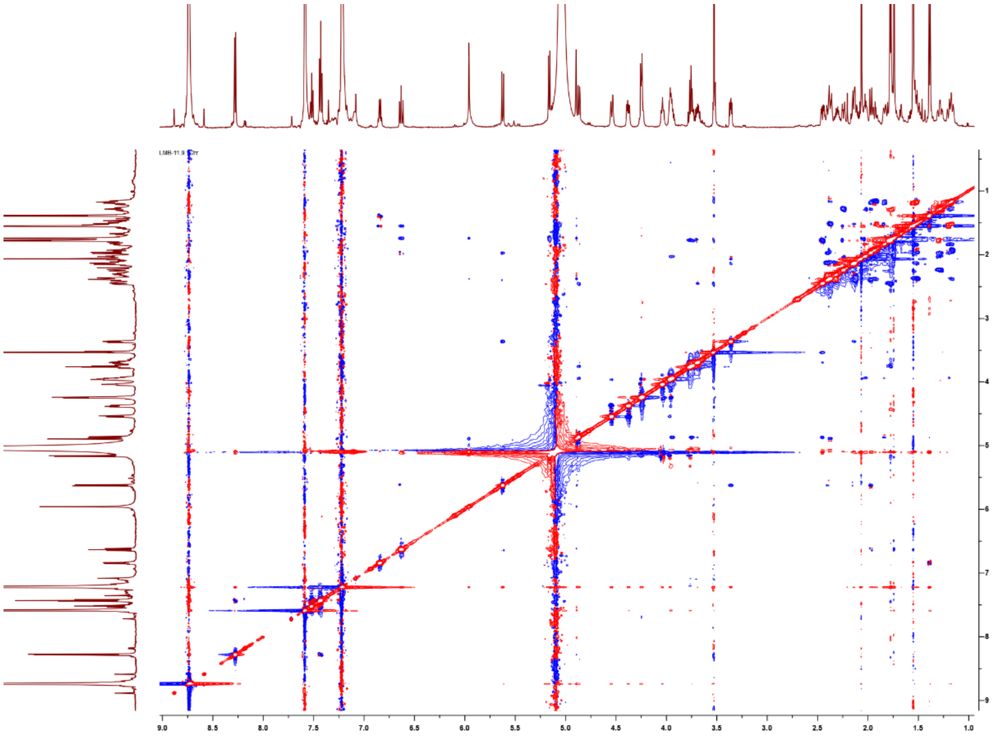

Figure S27. ^{1}H-^{1}H COSY (600/150 MHz, Pyridine-d_5) spectrum of Marsdeoside D (**4**).

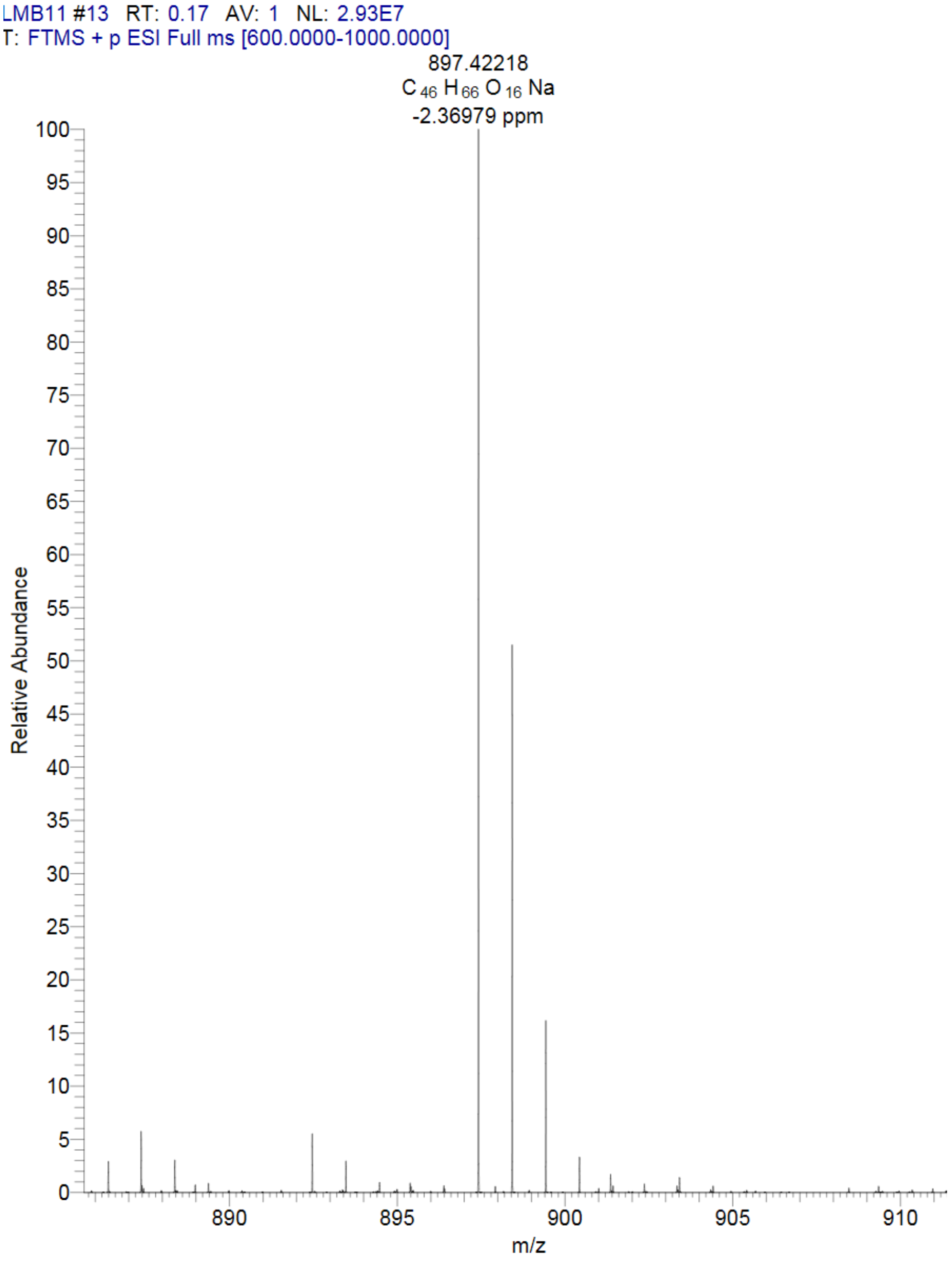

Figure S28. HRESIMS spectrum of Marsdeoside D (**4**).

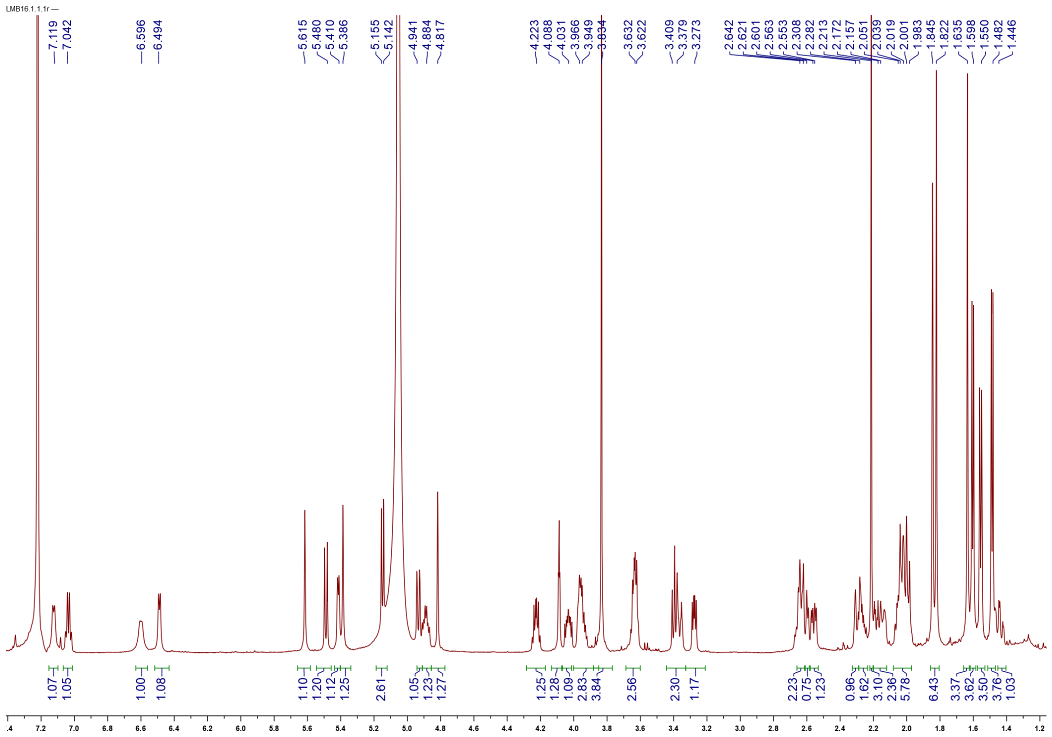

Figure S29. ^{1}H NMR (600 MHz, Pyridine-d$_5$) spectrum of Marsdeoside E (**5**).

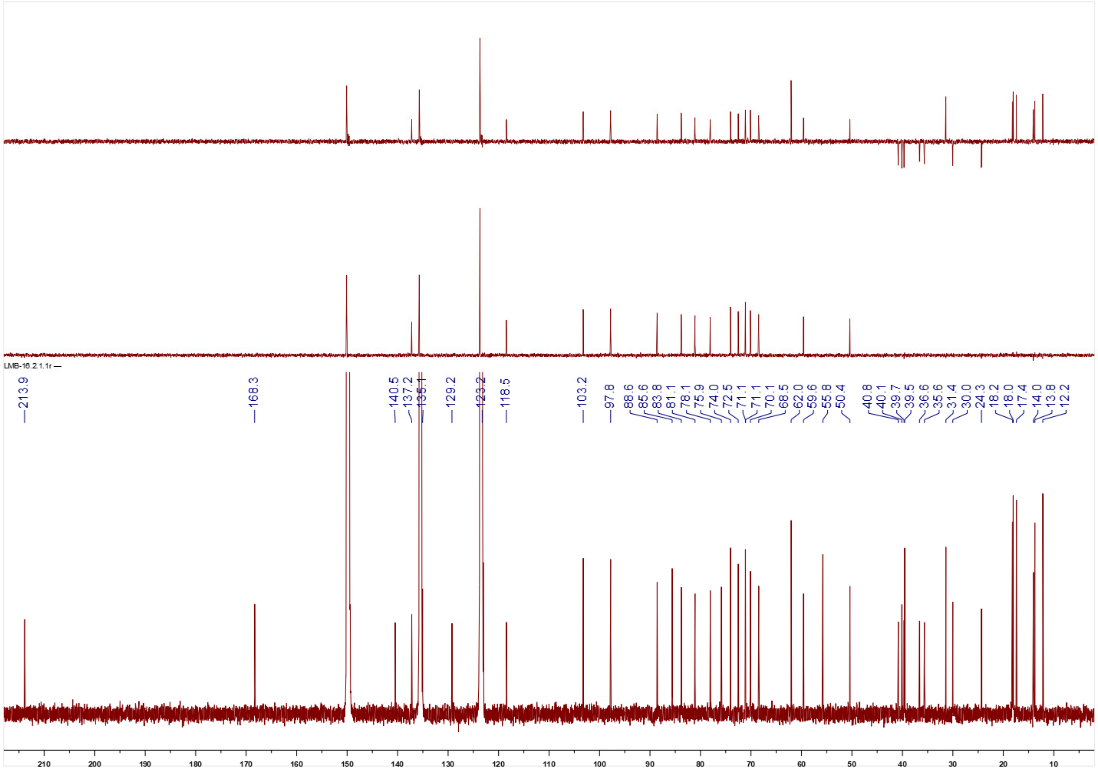

Figure S30. ^{13}C NMR (150 MHz, Pyridine-d$_5$) spectrum of Marsdeoside E (**5**).

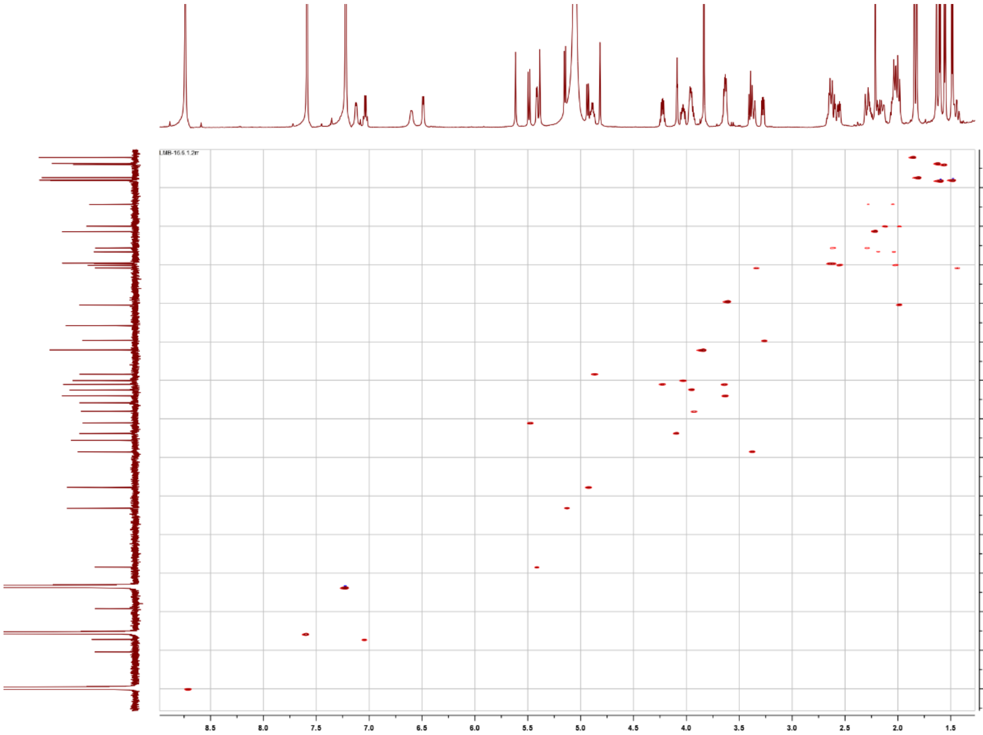

Figure S31. HSQC (600/150 MHz, Pyridine-d_5) spectrum of Marsdeoside E (**5**).

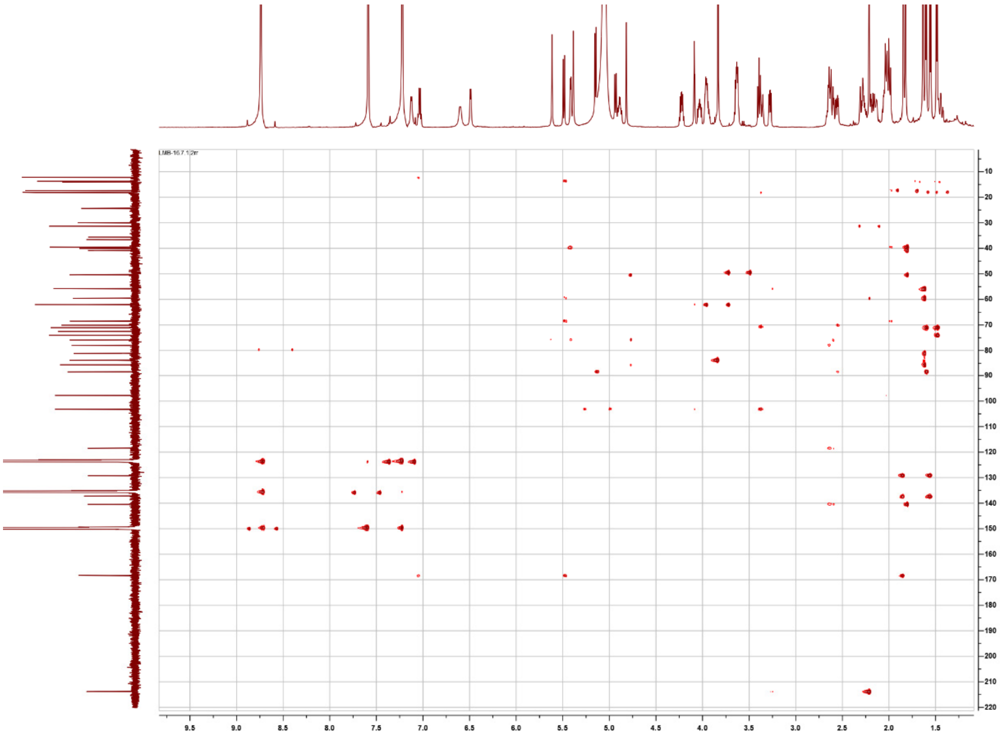

Figure S32. HMBC (600/150 MHz, Pyridine-d_5) spectrum of Marsdeoside E (**5**).

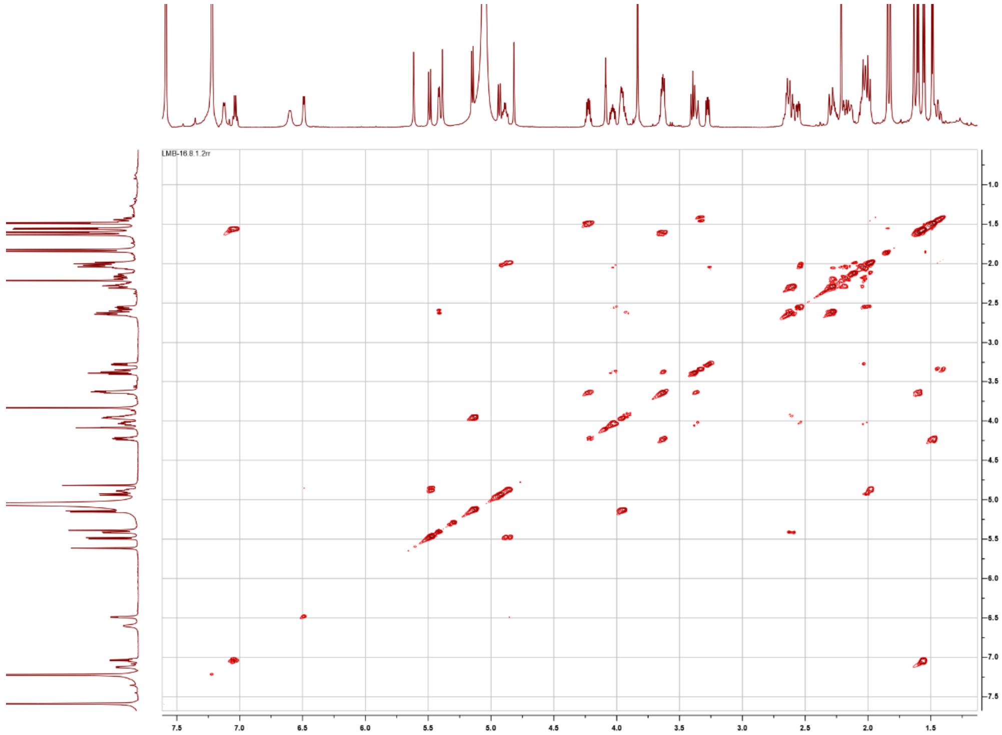

Figure S33. ^{1}H-^{1}H COSY (600/150 MHz, Pyridine-d$_5$) spectrum of Marsdeoside E (**5**).

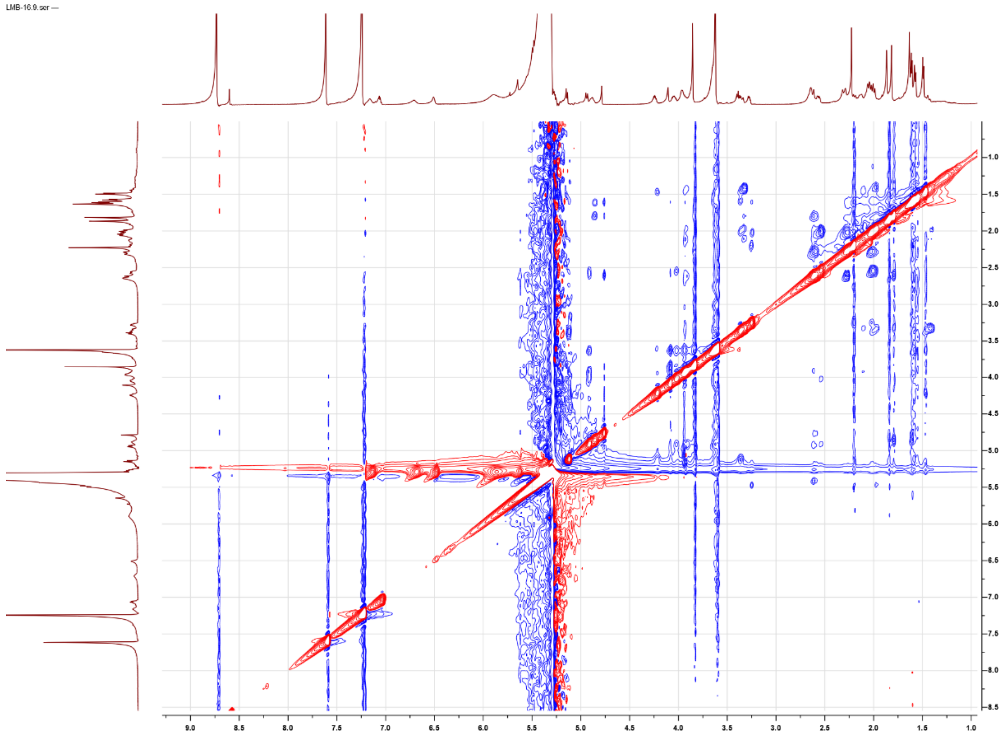

Figure S34. NOESY (600/150 MHz, Pyridine-d$_5$) spectrum of Marsdeoside E (**5**).

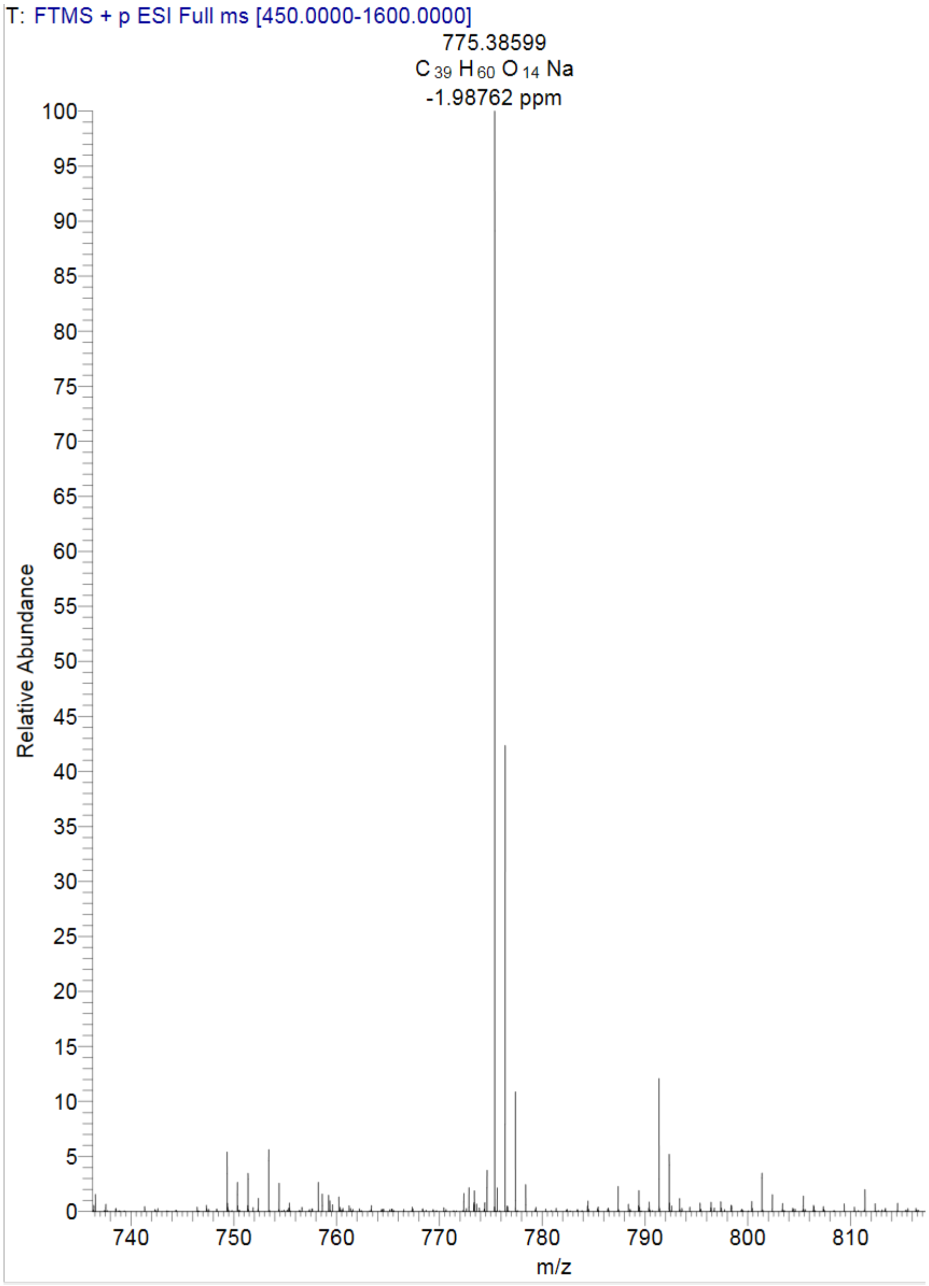

Figure S35. HRESIMS spectrum of Marsdeoside E (**5**).

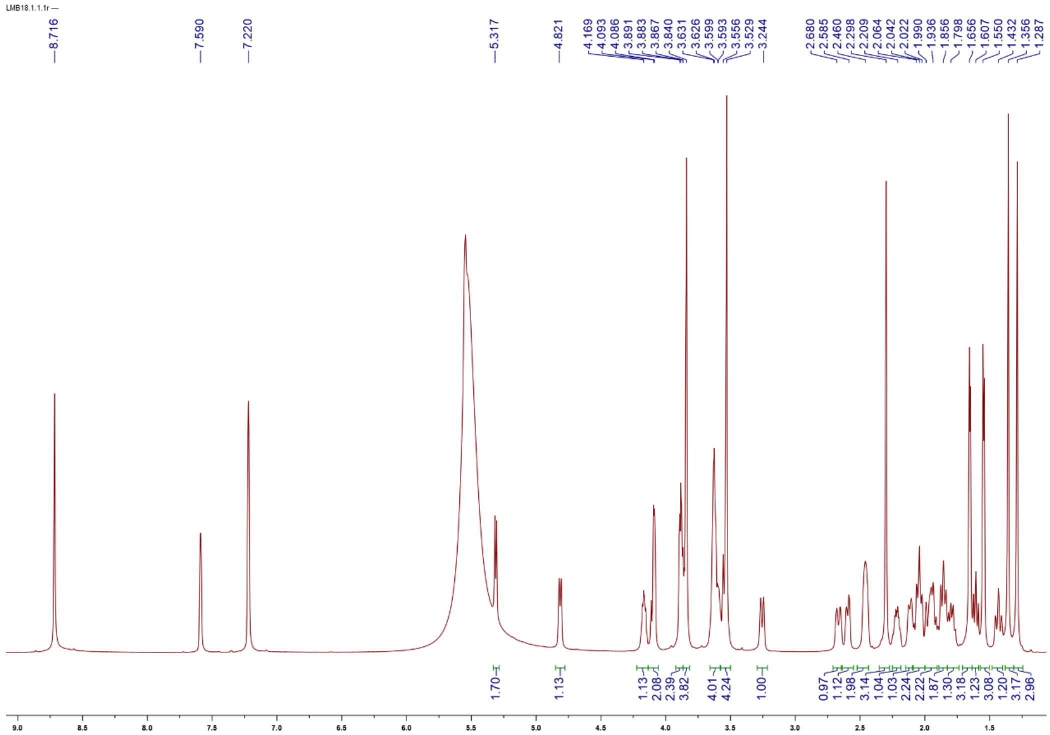

Figure S36. ^{1}H NMR (600 MHz, Pyridine-d_5) spectrum of Marsdeoside F (**6**).

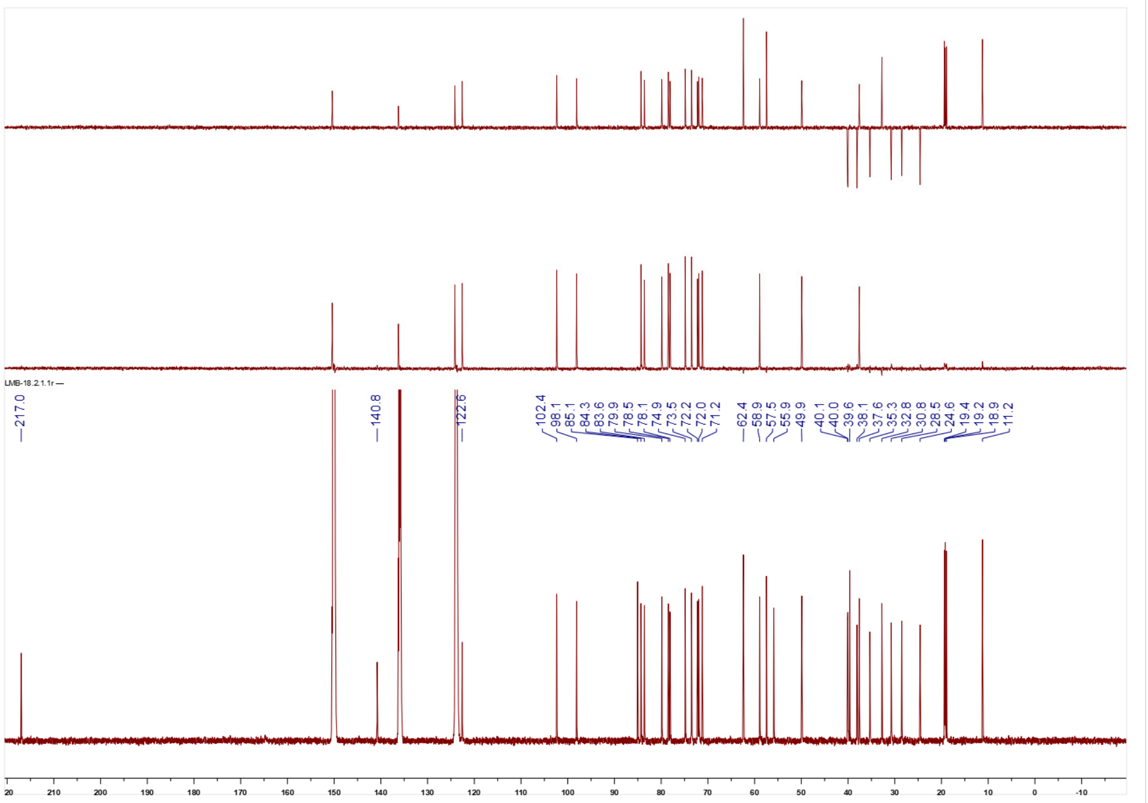

Figure S37. ^{13}C NMR (150 MHz, Pyridine-d_5) spectrum of Marsdeoside F (**6**).

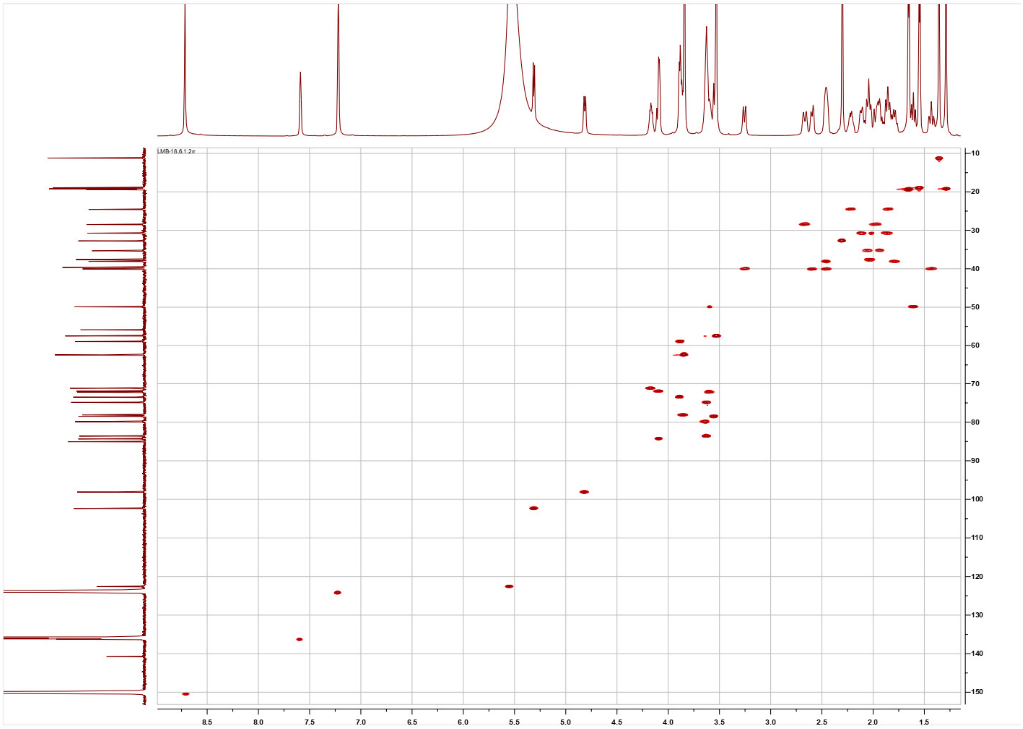

Figure S38. HSQC (600/150 MHz, Pyridine-d5) spectrum of Marsdeoside F (**6**).

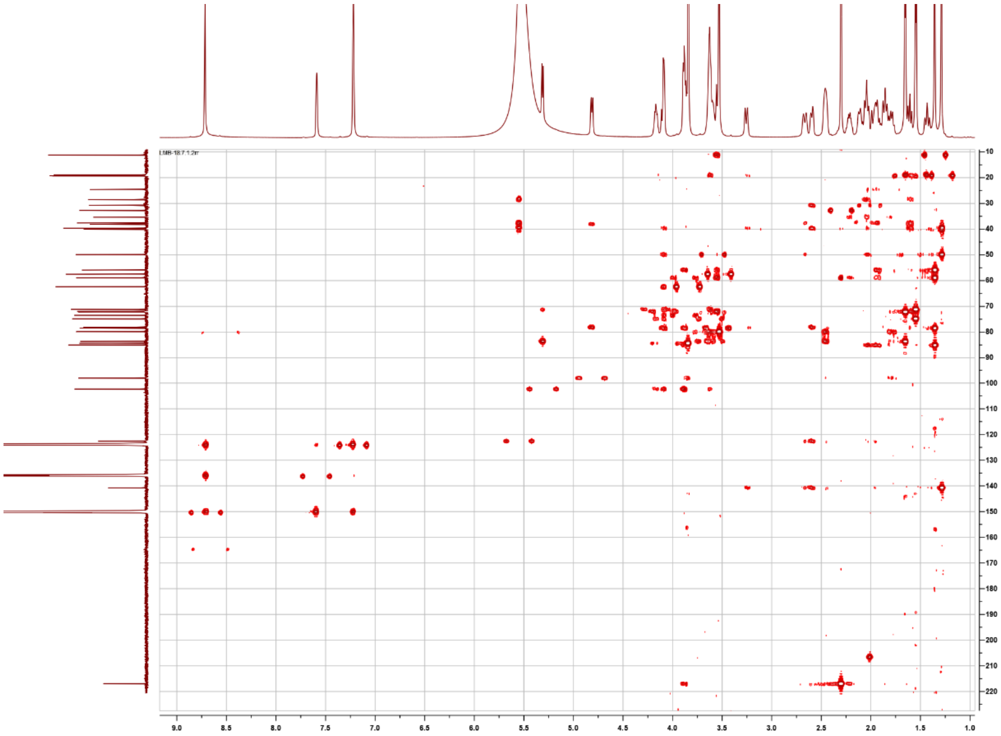

Figure S39. HMBC (600/150 MHz, Pyridine-d5) spectrum of Marsdeoside F (**6**).

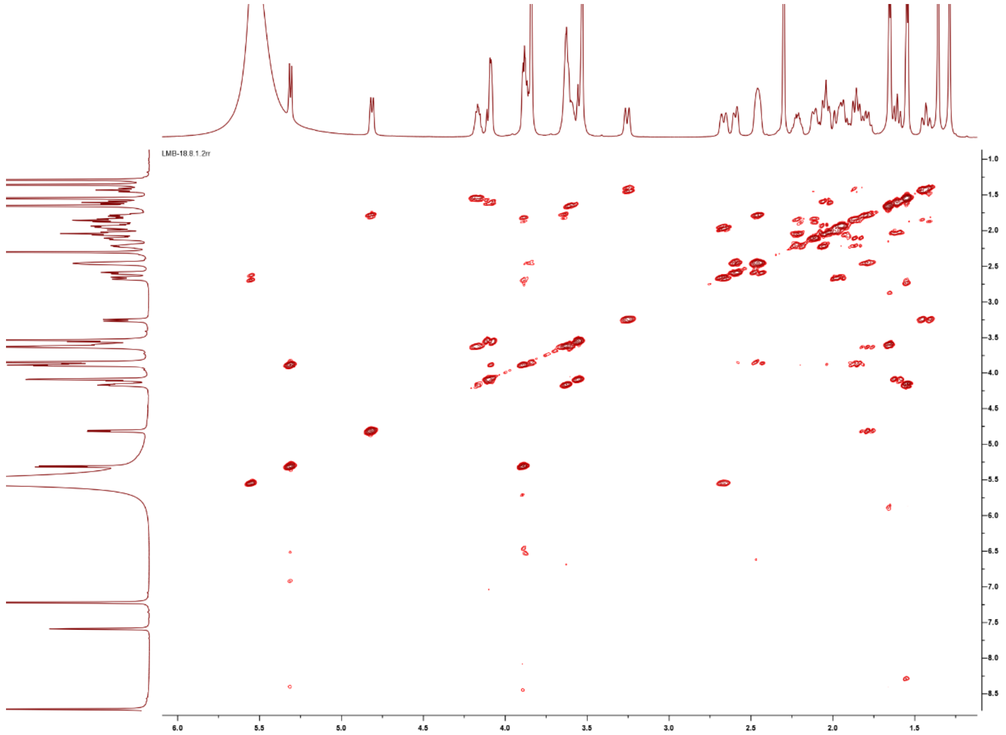

Figure S40. ^{1}H-^{1}H COSY (600/150 MHz, Pyridine-d_5) spectrum of Marsdeoside F (**6**).

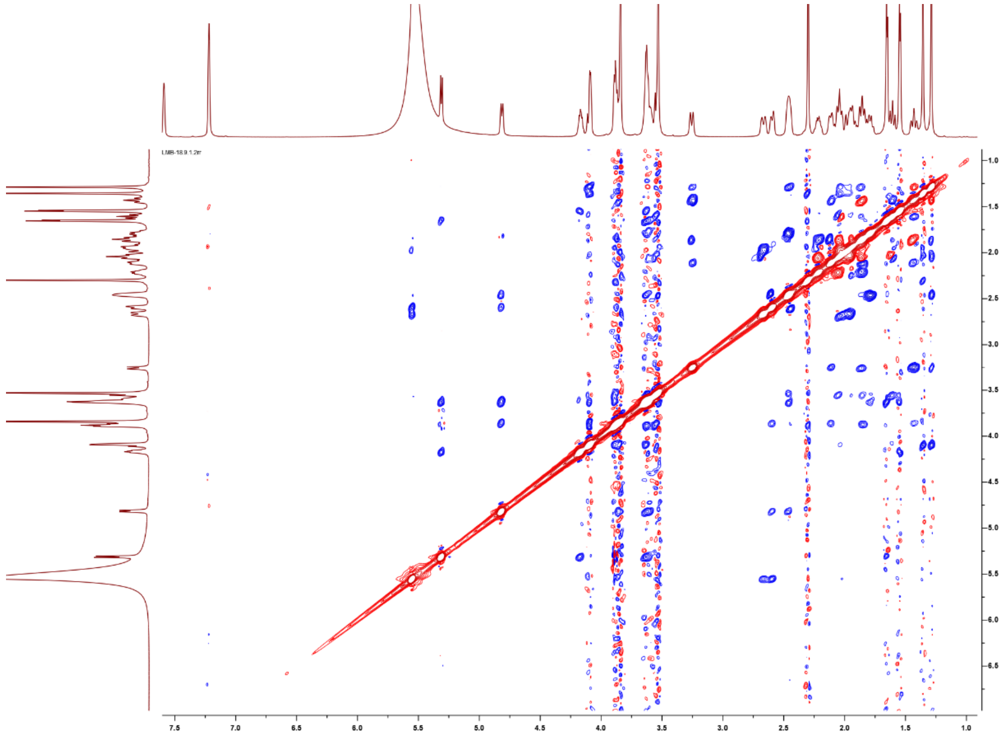

Figure S41. NOESY (600/150 MHz, Pyridine-d_5) spectrum of Marsdeoside F (**6**).

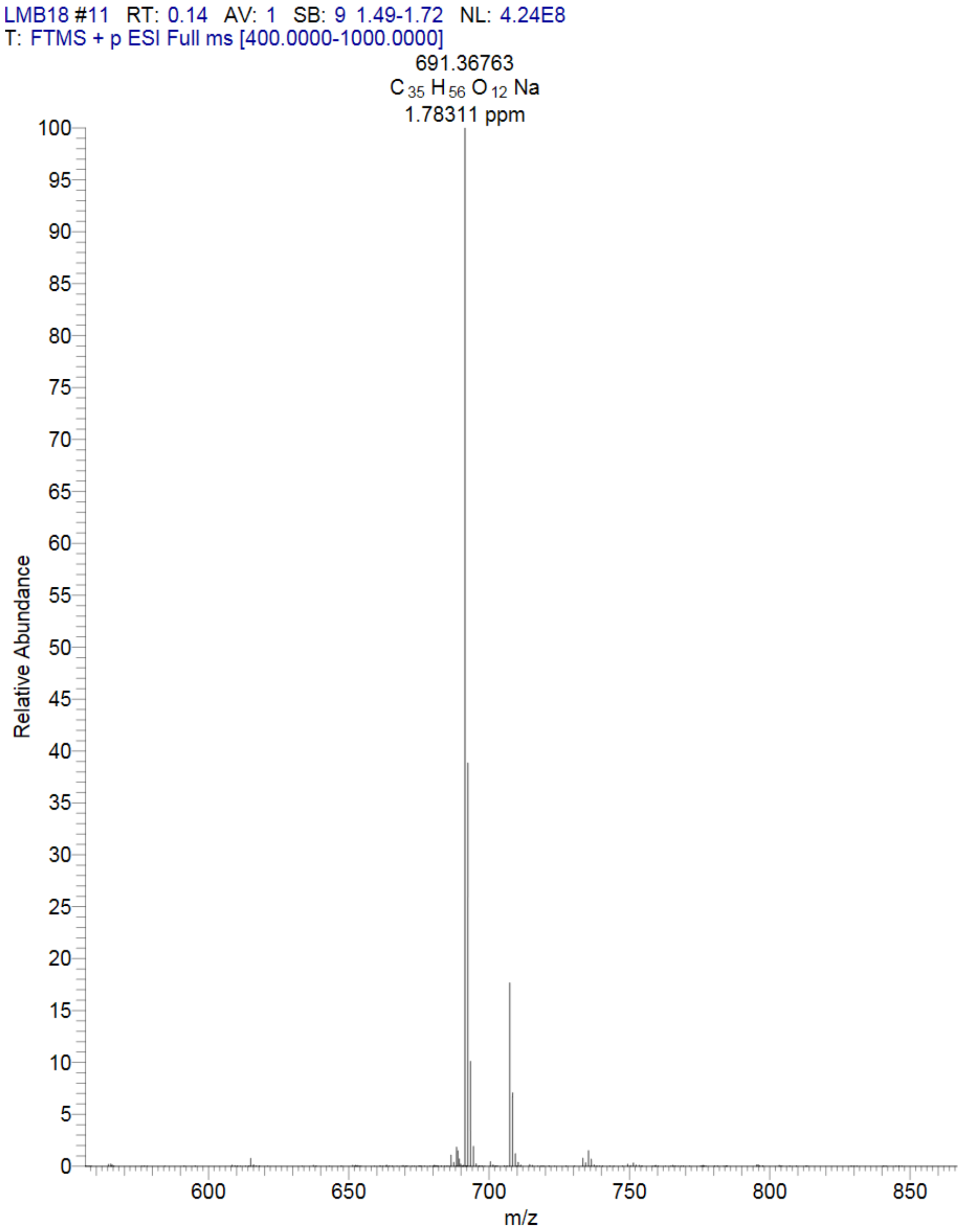

Figure S42. HRESIMS spectrum of Marsdeoside F (**6**).

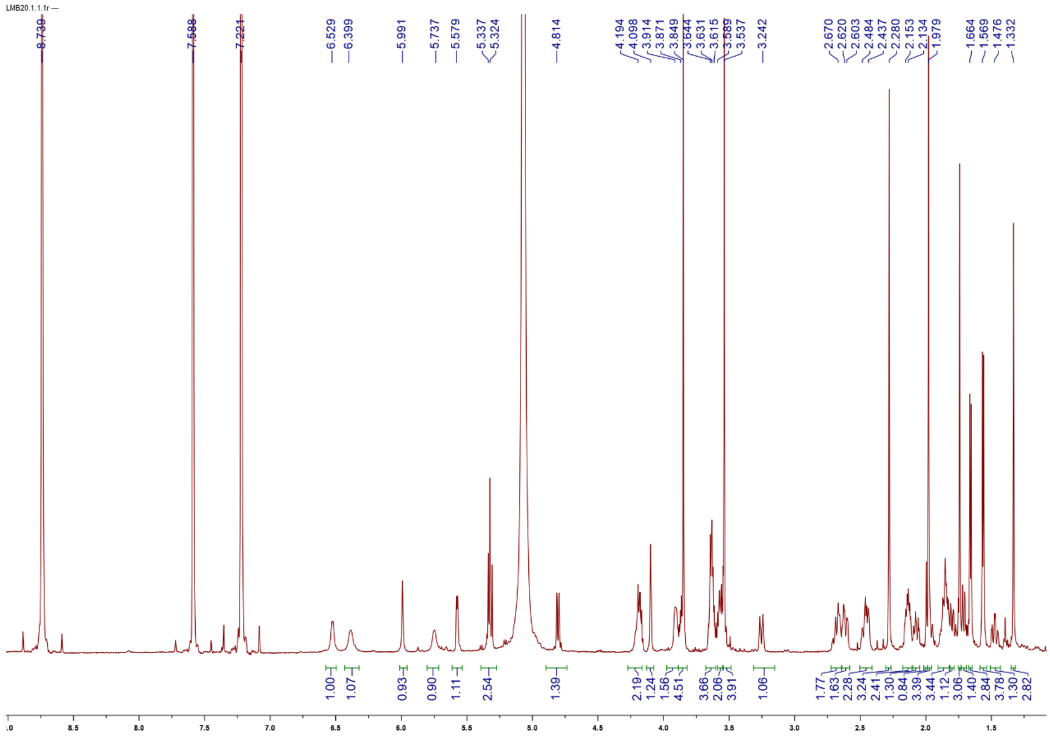

Figure S43. ^{1}H NMR (600 MHz, Pyridine-d$_5$) spectrum of Marsdeoside G (**7**).

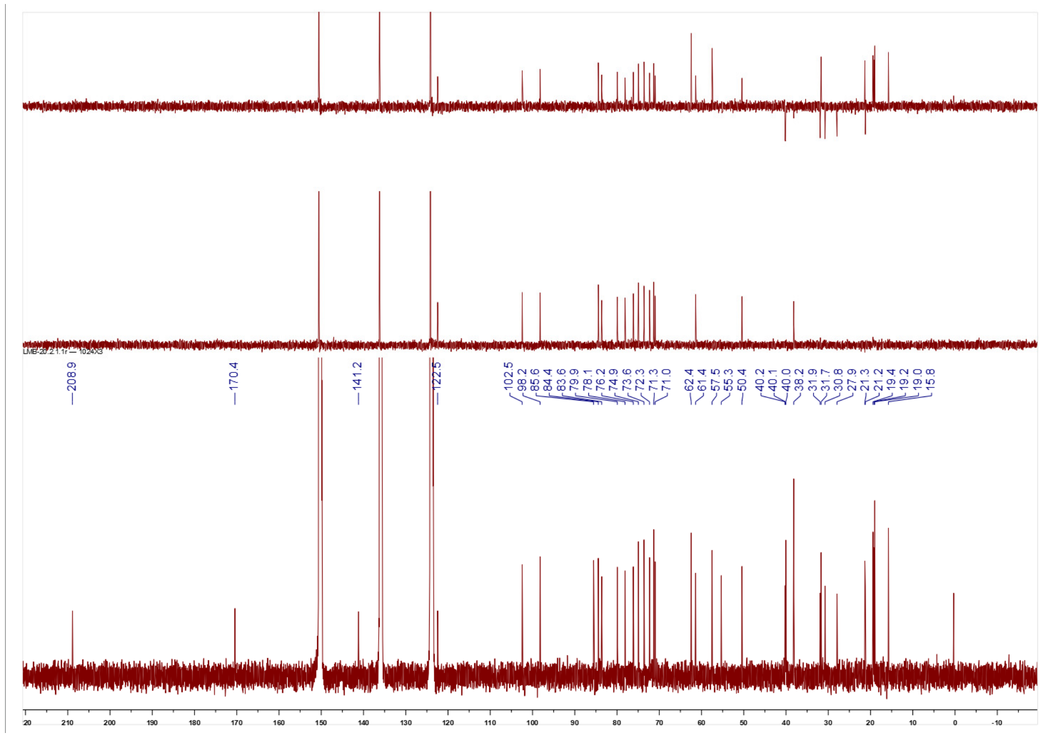

Figure S44. ^{13}C NMR (150 MHz, Pyridine-d$_5$) spectrum of Marsdeoside G (**7**).

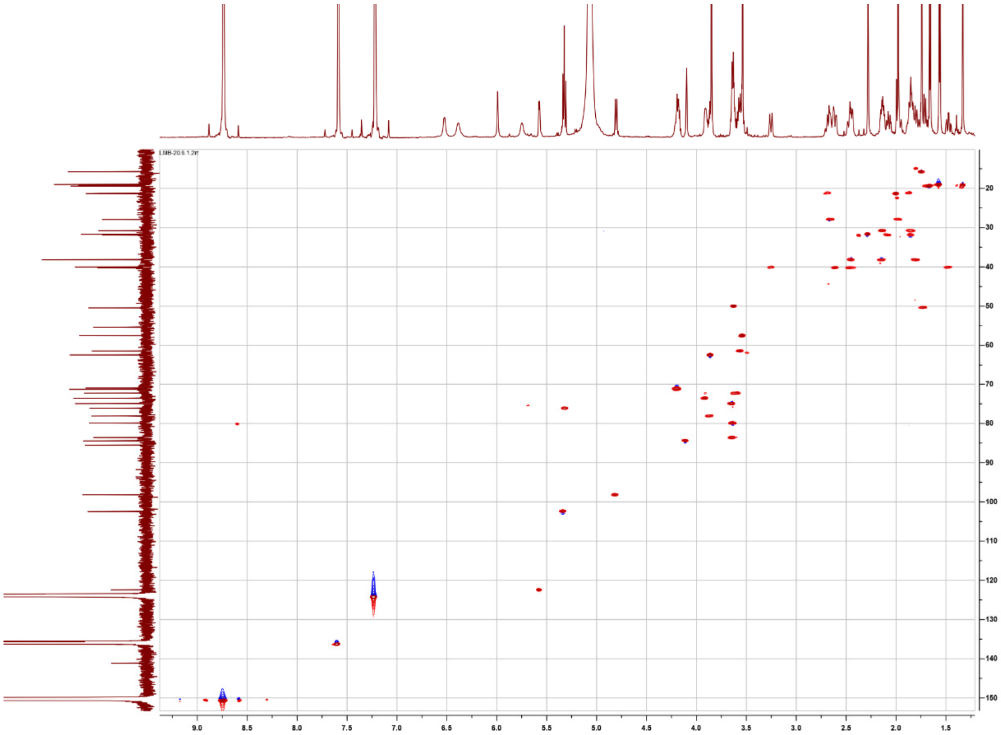

Figure S45. HSQC (600/150 MHz, Pyridine-d5) spectrum of Marsdeoside G (**7**).

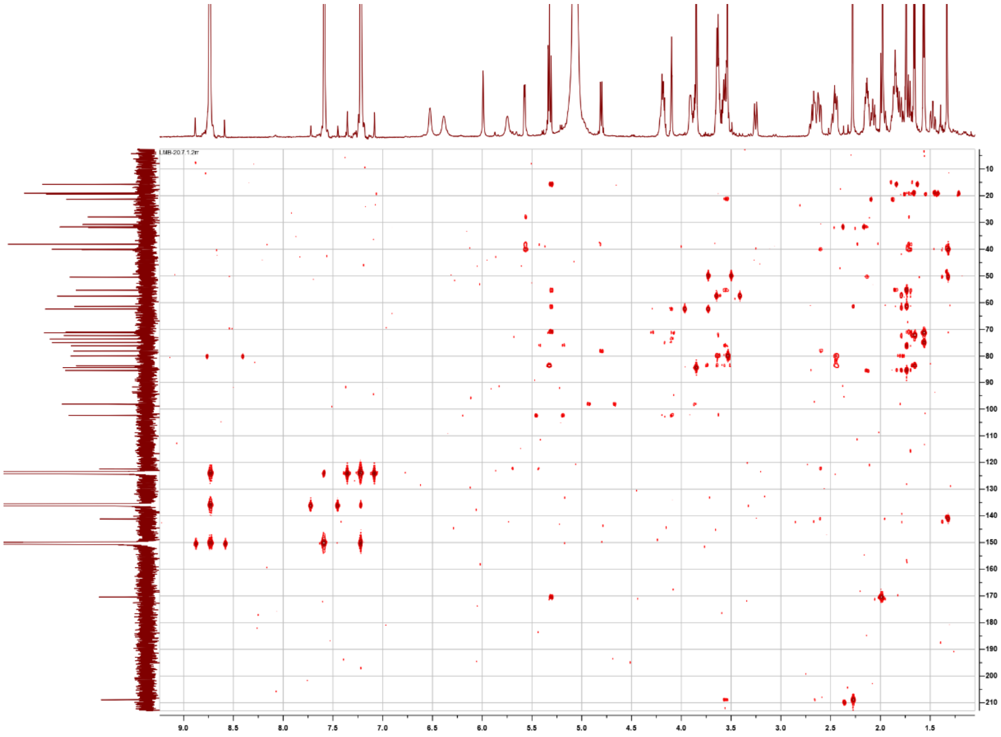

Figure S46. HMBC (600/150 MHz, Pyridine-d5) spectrum of Marsdeoside G (**7**).

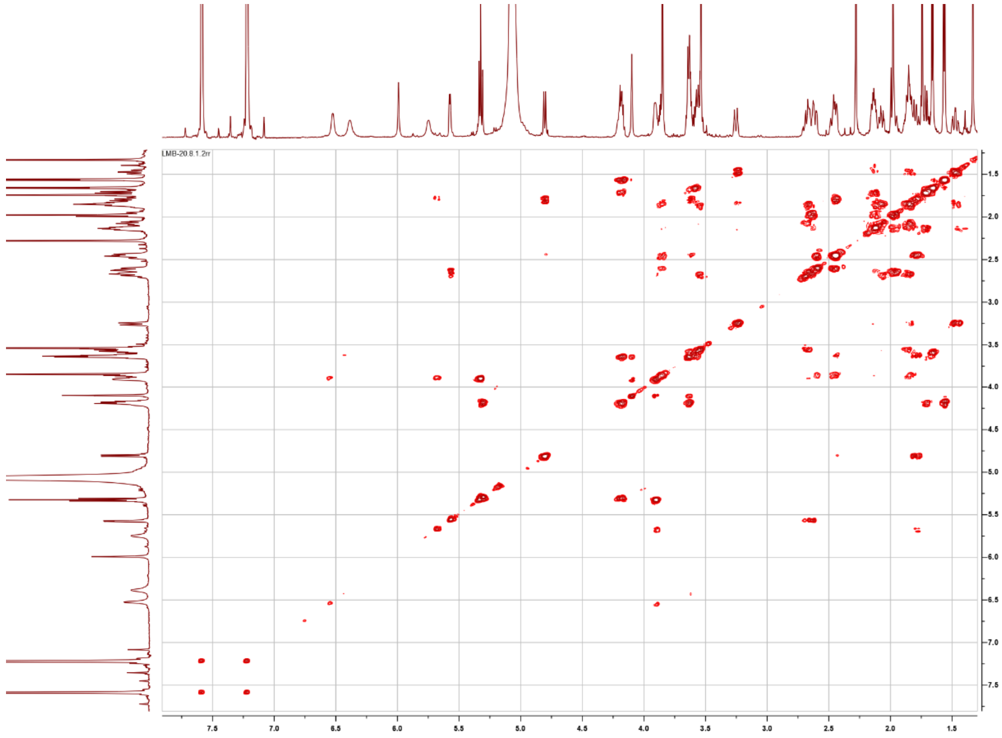

Figure S47. ^{1}H-^{1}H COSY (600/150 MHz, Pyridine-d5) spectrum of Marsdeoside G (**7**).

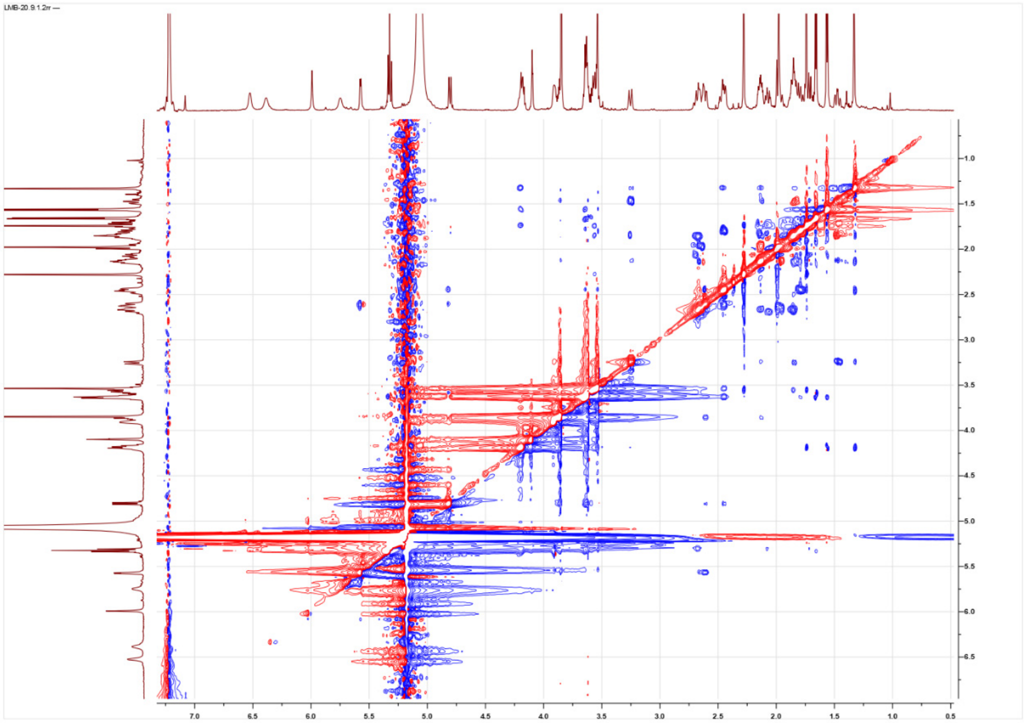

Figure S48. NOESY (600/150 MHz, Pyridine-d5) spectrum of Marsdeoside G (**7**).

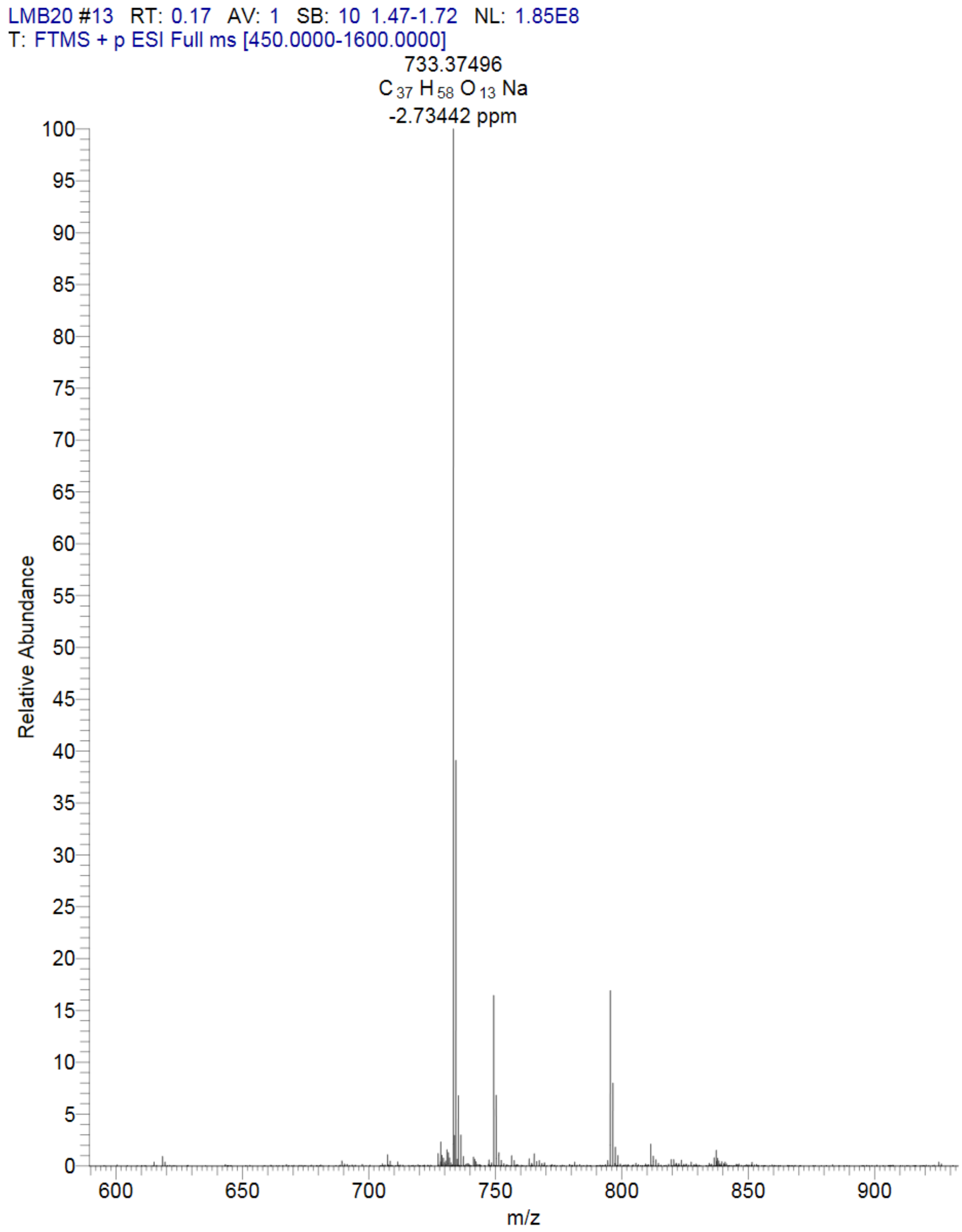

Figure S49. HRESIMS spectrum of Marsdeoside G (**7**).

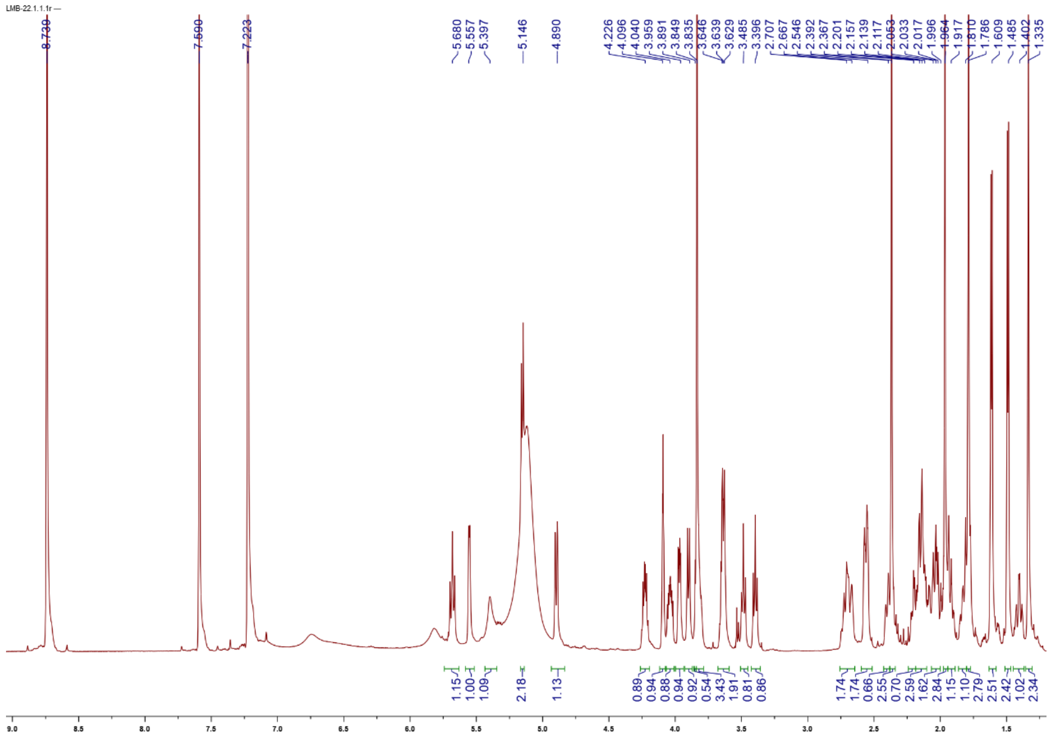

Figure S50. ^{1}H NMR (600 MHz, Pyridine-d$_5$) spectrum of Marsdeoside H (**8**).

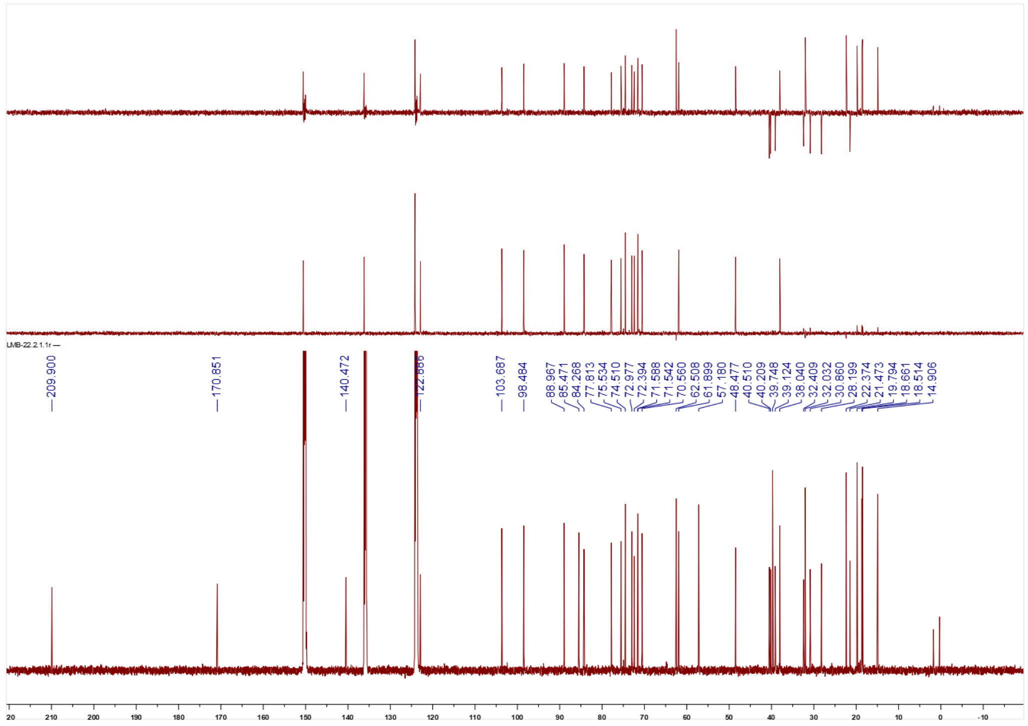

Figure S51. ^{13}C NMR (150 MHz, Pyridine-d$_5$) spectrum of Marsdeoside H (**8**).

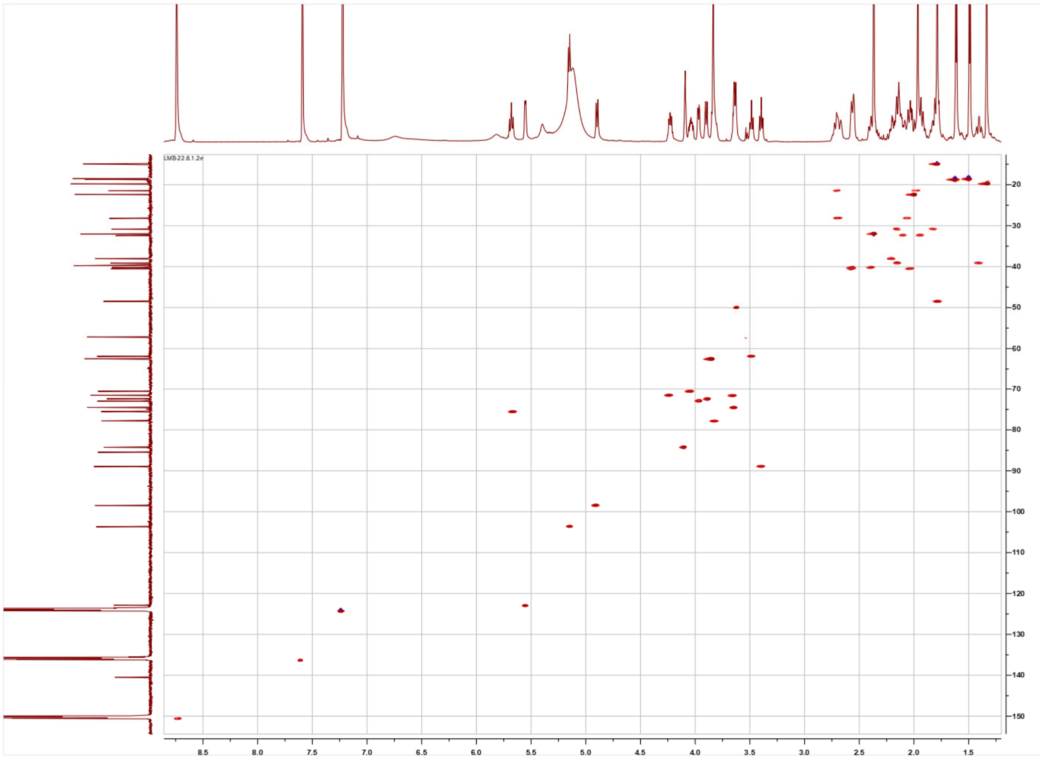

Figure S52. HSQC (600/150 MHz, Pyridine-d_5) spectrum of Marsdeoside H (**8**).

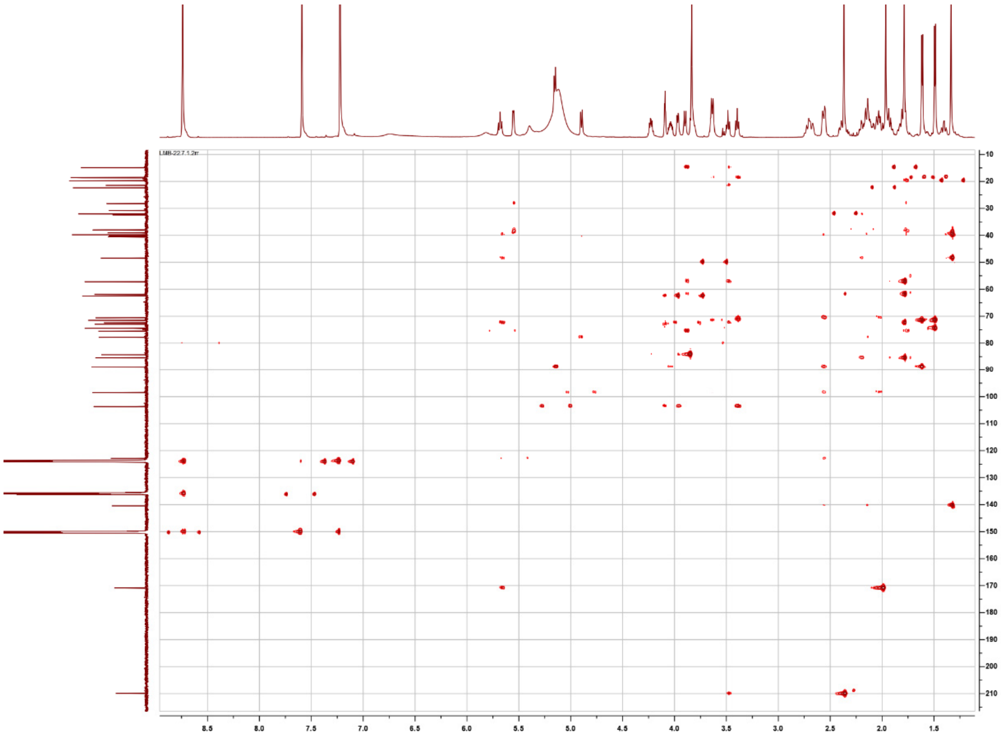

Figure S53. HMBC (600/150 MHz, Pyridine-d_5) spectrum of Marsdeoside H (**8**).

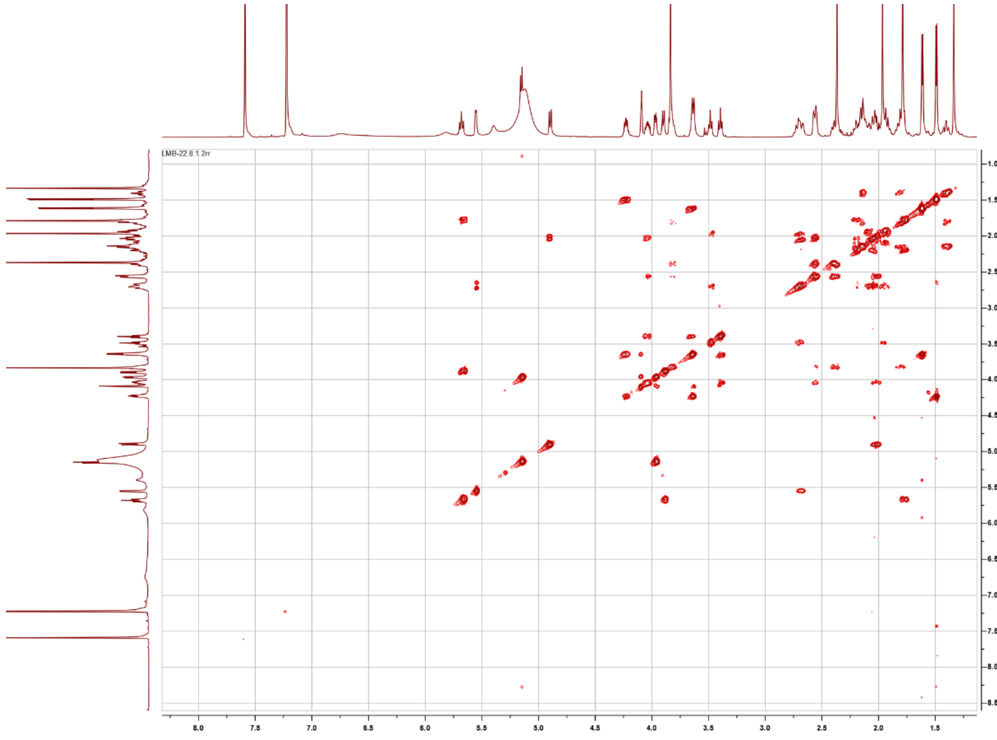

Figure S54. ^{1}H-^{1}H COSY (600/150 MHz, Pyridine-d$_5$) spectrum of Marsdeoside H (**8**).

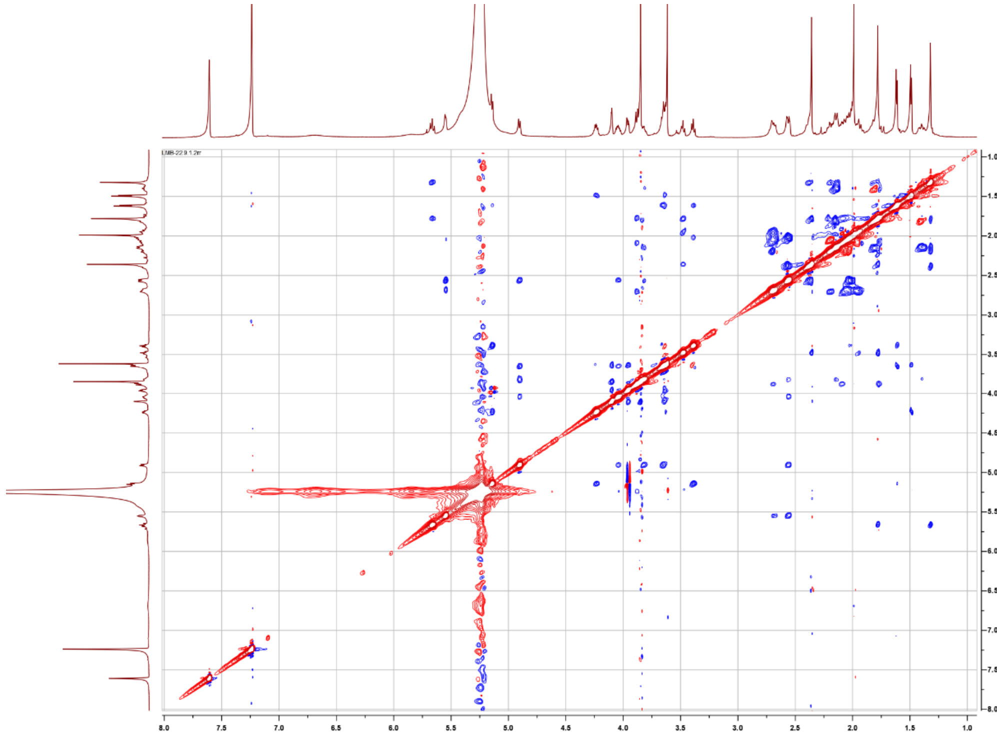

Figure S55. NOESY (600/150 MHz, Pyridine-d$_5$) spectrum of Marsdeoside H (**8**).

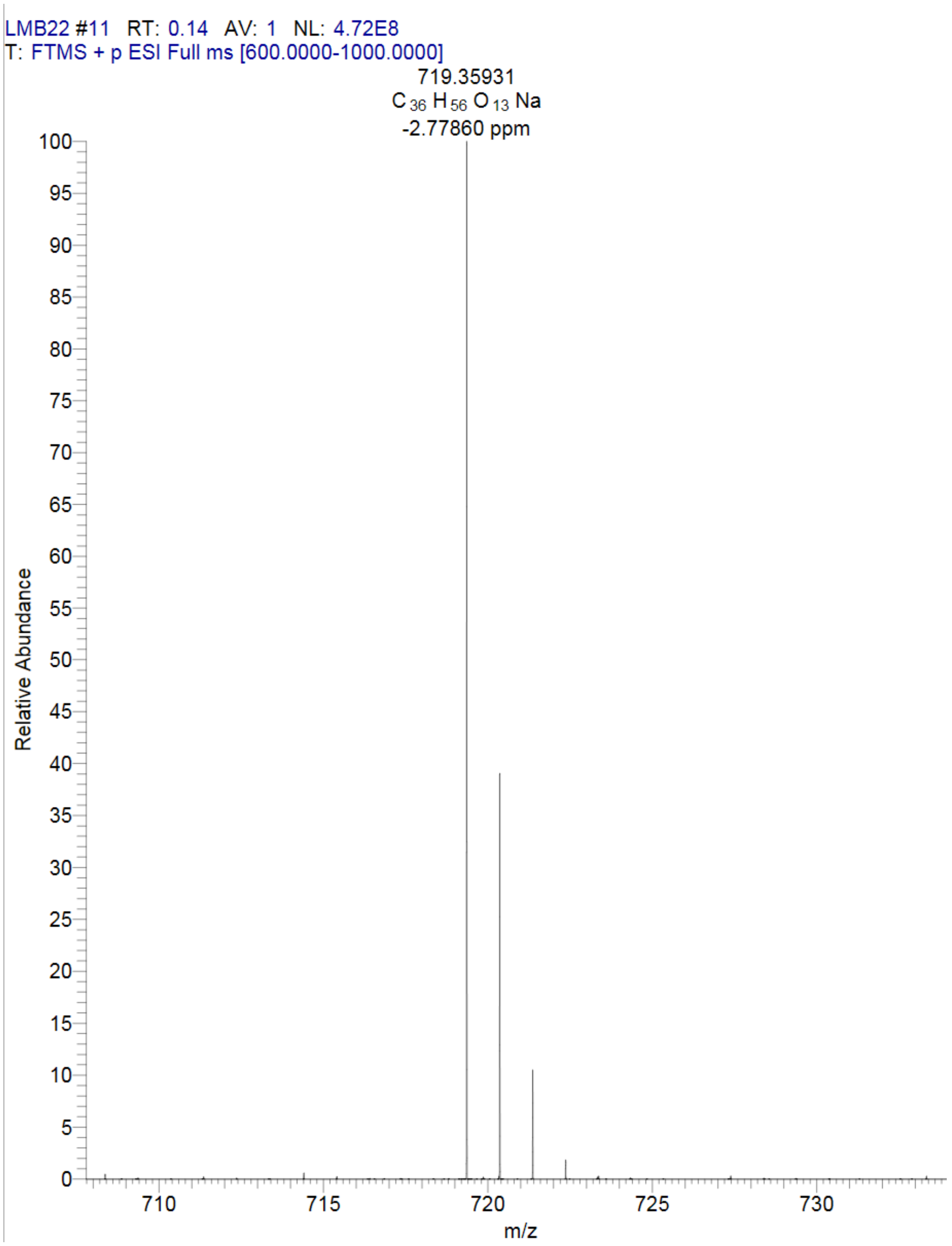

Figure S56. HRESIMS spectrum of Marsdeoside H (**8**).

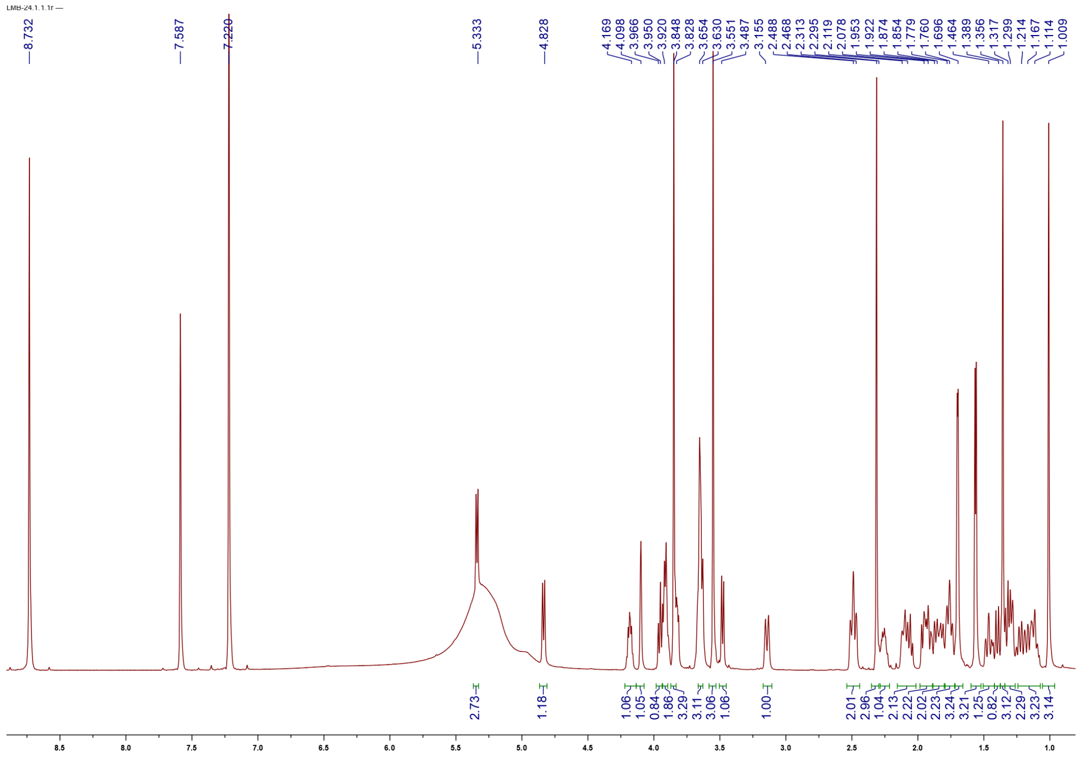

Figure S57. ^{1}H NMR (600 MHz, Pyridine-d_5) spectrum of Marsdeoside H (**9**).

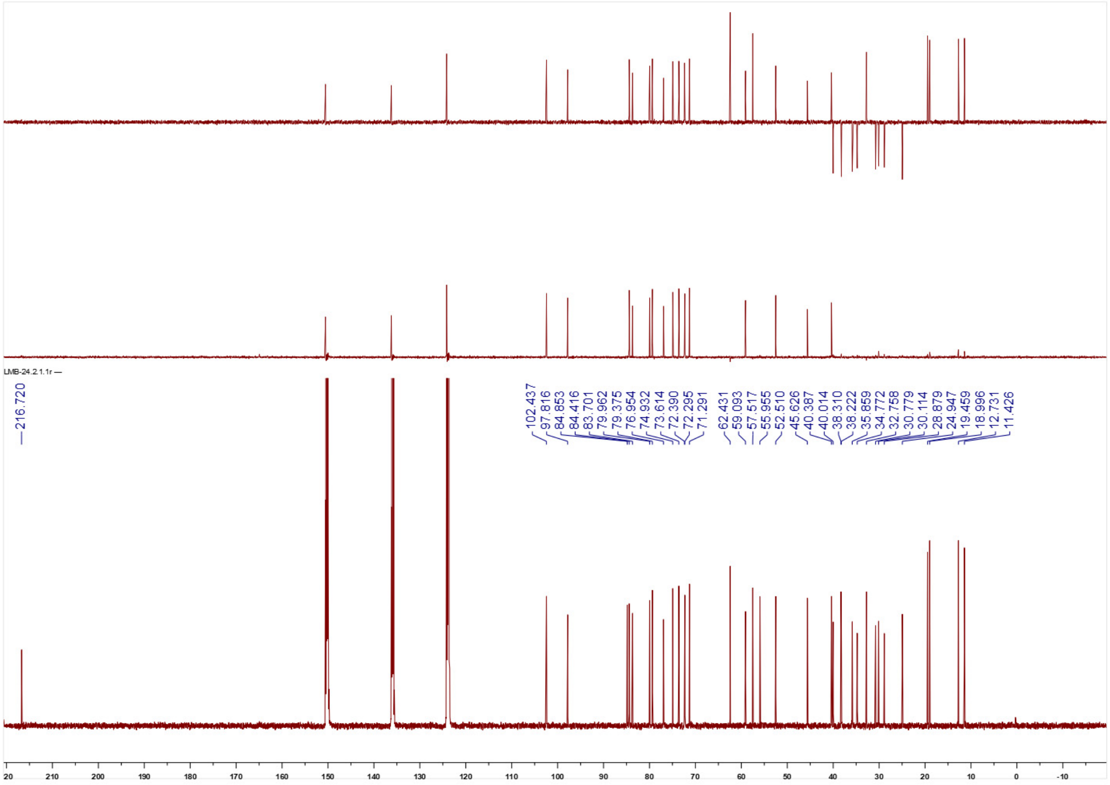

Figure S58. ^{13}C NMR (150 MHz, Pyridine-d_5) spectrum of Marsdeoside H (**9**).

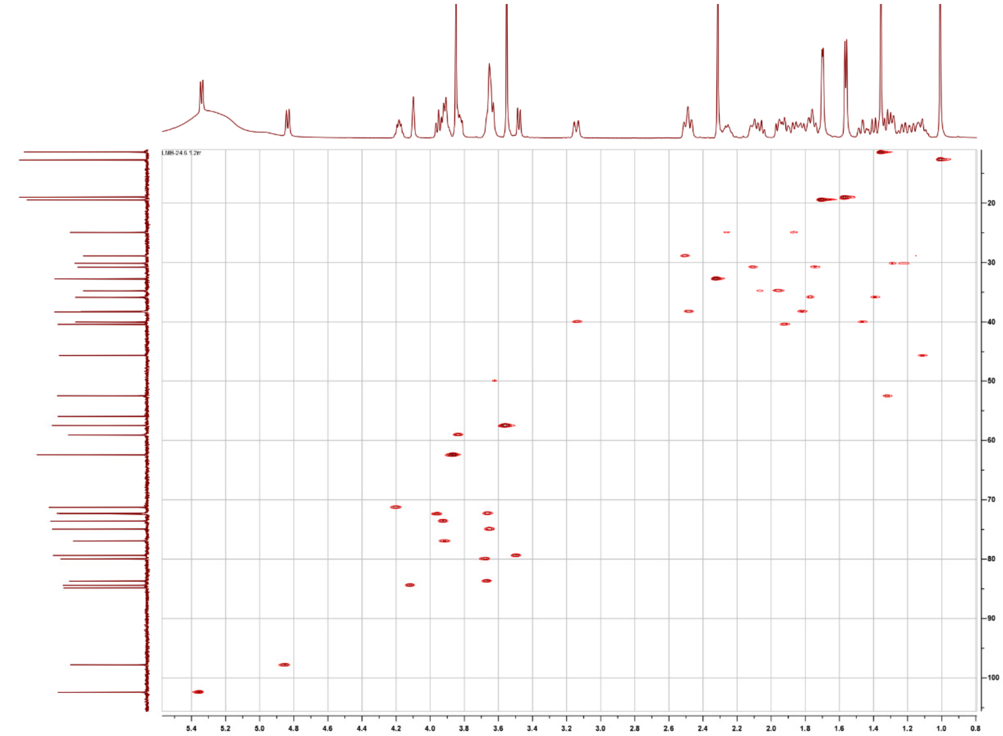

Figure S59. HSQC (600/150 MHz, Pyridine-d5) spectrum of Marsdeoside H (**9**).

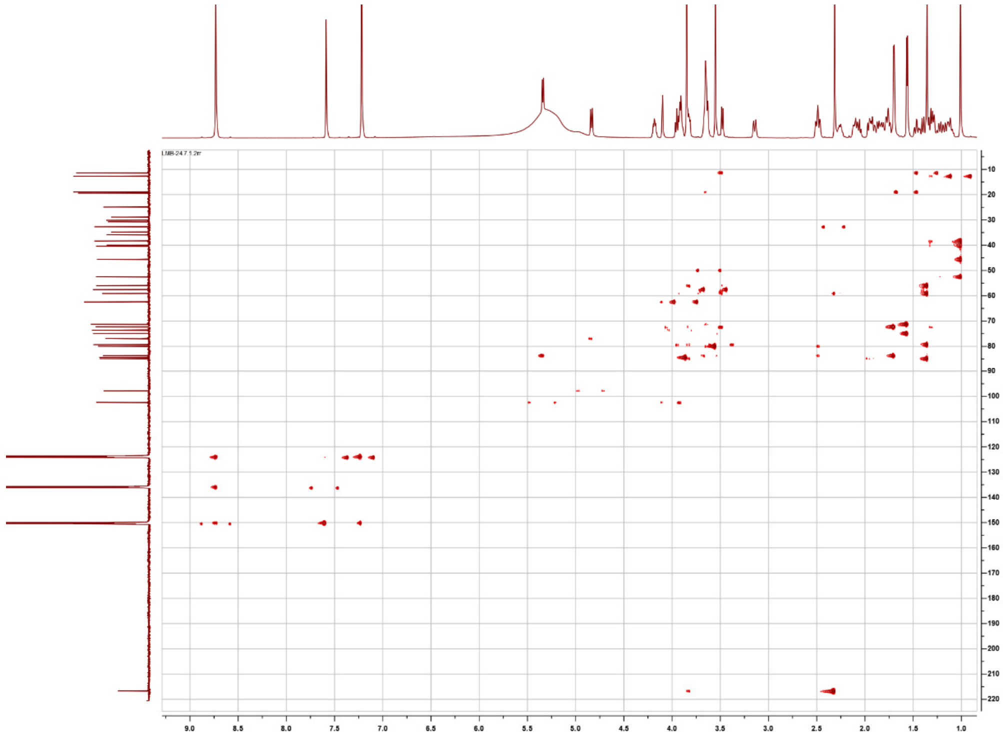

Figure S60. HMBC (600/150 MHz, Pyridine-d5) spectrum of Marsdeoside H (**9**).

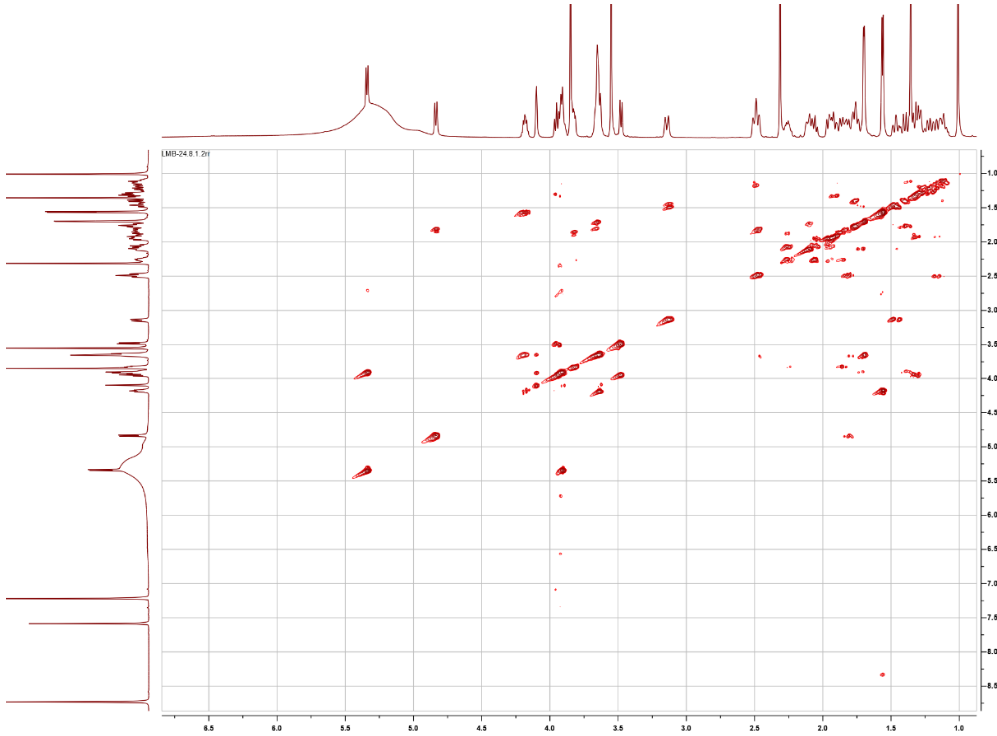

Figure S61. ^{1}H-^{1}H COSY (600/150 MHz, Pyridine-d_5) spectrum of Marsdeoside H (**8**).

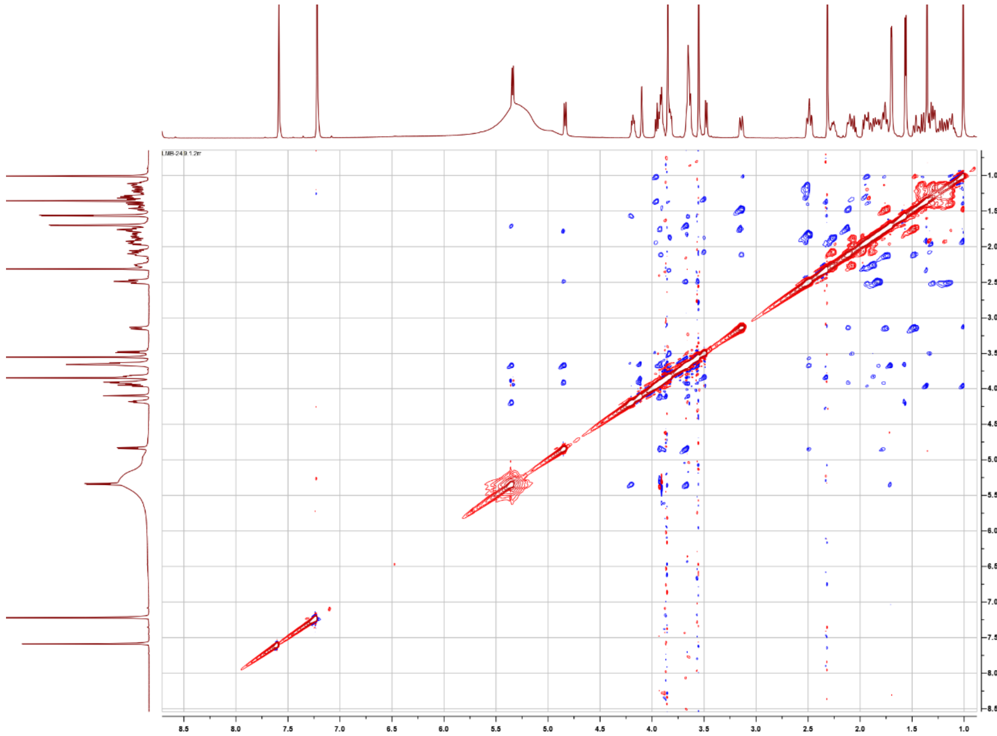

Figure S62. NOESY (600/150 MHz, Pyridine-d_5) spectrum of Marsdeoside H (**9**).

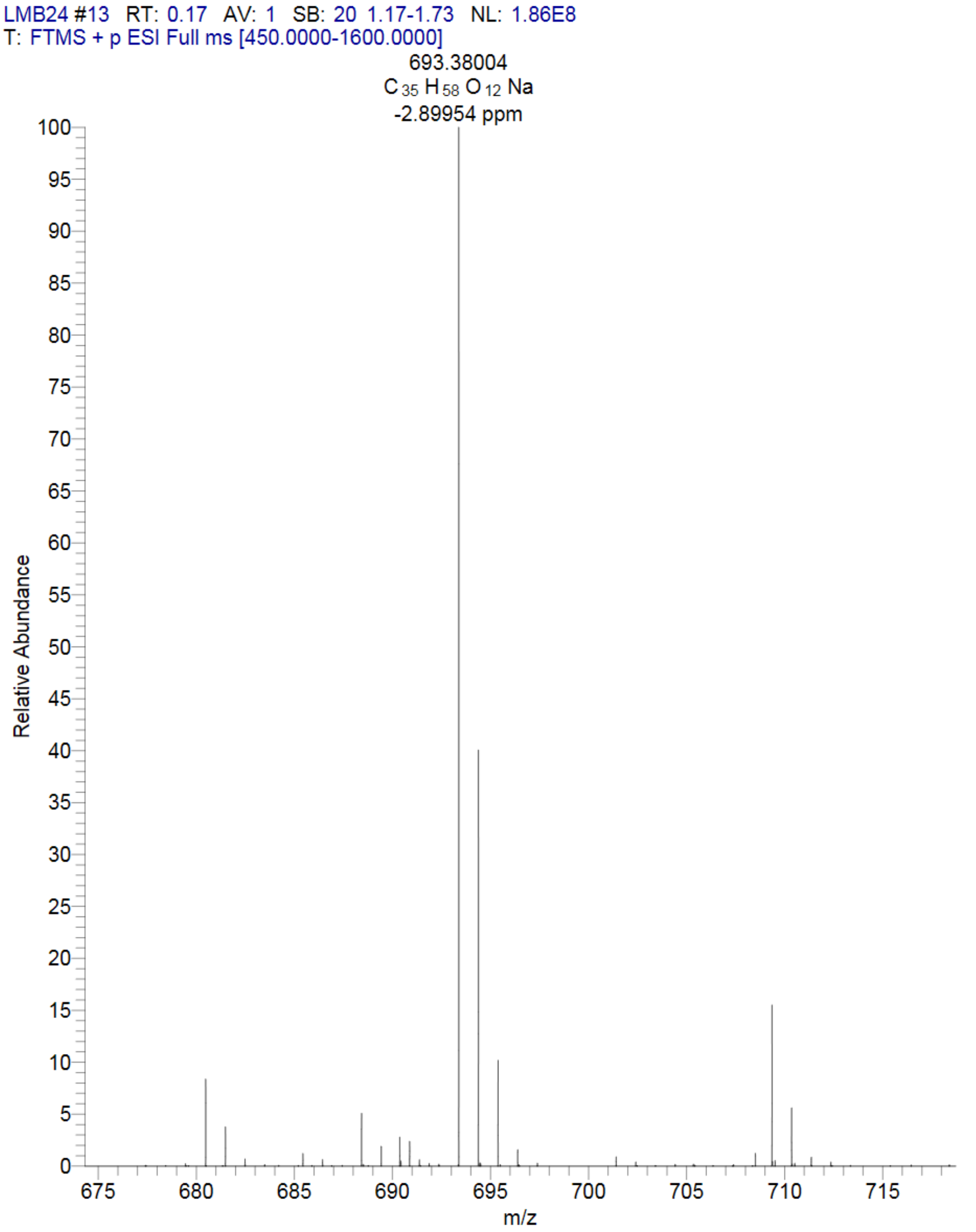

Figure S63. HRESIMS spectrum of Marsdeoside H (**9**).
